# Drug Repurposing on G Protein-Coupled Receptors Using a Computational Profiling Approach

**DOI:** 10.3389/fmolb.2021.673053

**Published:** 2021-05-07

**Authors:** Alessandra de Felice, Simone Aureli, Vittorio Limongelli

**Affiliations:** ^1^Faculty of Biomedical Sciences, Euler Institute, Università della Svizzera italiana (USI), Lugano, Switzerland; ^2^Department of Pharmacy, University of Naples “Federico II”, Naples, Italy

**Keywords:** GPCR, drug repurposing, molecular docking, drug design, drug repositioning, protein sequence profile alignment

## Abstract

G protein-coupled receptors (GPCRs) are the largest human membrane receptor family regulating a wide range of cell signaling. For this reason, GPCRs are highly desirable drug targets, with approximately 40% of prescribed medicines targeting a member of this receptor family. The structural homology of GPCRs and the broad spectrum of applications of GPCR-acting drugs suggest an investigation of the cross-activity of a drug toward different GPCR receptors with the aim of rationalizing drug side effects, designing more selective and less toxic compounds, and possibly proposing off-label therapeutic applications. Herein, we present an original *in silico* approach named “Computational Profiling for GPCRs” (CPG), which is able to represent, in a one-dimensional (1D) string, the physico-chemical properties of a ligand–GPCR binding interaction and, through a tailored alignment algorithm, repurpose the ligand for a different GPCR. We show three case studies where docking calculations and pharmacological data confirm the drug repurposing findings obtained through CPG on 5-hydroxytryptamine receptor 2B, beta-2 adrenergic receptor, and M2 muscarinic acetylcholine receptor. The CPG code is released as a user-friendly graphical user interface with numerous options that make CPG a powerful tool to assist the drug design of GPCR ligands.

## Introduction

G protein-coupled receptors (GPCRs) are integral membrane proteins involved in the transduction of a wide range of signals from outside the cell to the cellular interior. They represent the largest and most pharmacologically relevant protein family—∼4% of the protein-coding genome (Fredriksson et al., 2003; Zhang et al., 2006). From a structural point of view, in spite of low sequence homology, all GPCRs share a common barrel tertiary structure composed of seven *trans*-membrane α-helices (TM1-7). Furthermore, some GPCRs have an additional α-helix (H8) at the C-terminal (Yeagle and Albert, 2007). The orthosteric binding site of endogenous ligands is typically located in the upper, extracellular part of the receptor, underneath the extracellular loop 2 (ECL2). At the intracellular level, GPCRs interact with the G-protein heterotrimer complex (Gαβγ) through a process allosterically modulated by ligand-induced conformational changes that activate a specific signal cascade based on the type of the interacting Gα-protein (Gs, Gi, Go, Gq/11, G12/13) (Zhang et al., 2006; Katritch et al., 2013). Through such mechanisms, GPCRs respond to a large variety of stimuli, regulating relevant processes including pain, immune response, inflammation, mood regulation, blood pressure regulation, neurotransmission, and many others (Katritch et al., 2013; Venkatakrishnan et al., 2013; Gacasan et al., 2017). As a consequence, GPCRs are the most prominent molecular targets in drug design, targeted by ∼40% of prescribed drugs (25 of the 100 top-selling) (Thomsen et al., 2005; Rask-Andersen et al., 2011).

In this framework, elucidating the cross-activity of a drug toward diverse GPCRs aids in rationalizing its side effects, proposing off-label therapeutic applications (clinical use for a disease different from that the drug was designed for), and designing novel, more selective GPCRs ligands. With this in mind, we have developed an original *in silico* approach named “Computational Profiling for GPCRs” (CPG), which takes into account both the GPCR sequence and the ligand-GPCR binding interactions to repurpose compounds meant to target one specific GPCR as novel ligands for a different GPCR receptor. Drug repurposing is a fast and safe drug discovery approach that has been successfully employed to identify drugs on the market—therefore considered safe—as new ligands for a molecular target different from the original one (Pushpakom et al., 2019). Our approach is made possible due to the conservative nature of the GPCR tertiary structure and the orthosteric binding site location. In particular, our method (i) “translates” the ligand–protein interaction patterns into a one-dimensional (1D) profile; (ii) aligns the 1D strings coming from different GPCR–ligand complexes; and, finally, (iii) selects the most similar ones to identify drug candidates for drug repurposing. The CPG is designed as a graphical user interface (GUI), integrated into the worldwide-used Visual Molecular Dynamics (VMD) software (Humphrey et al., 1996).

Using CPG, the user is able to process ligand–GPCR complexes obtained from the Protein Data Bank (PDB, Berman et al., 2007) or molecular binding simulations and achieve a fast determination of ligand-GPCR binding similarities. While the workflow of CPG can be applied to any ligand in the identification of potential off-targets, it reveals its potency when employed with market-approved drugs. Indeed, it repurposes a drug for a GPCR different from its original one, thus paving the way to possible off-label therapeutic applications, alternative from that originally intended. At the same time, by identifying a novel GPCR target for the drug, CPG may help to rationalize the unexplained side effects of the drug. Finally, data regarding the similarity between different drug–GPCR complexes generated by CPG are useful to guide the development of novel, more selective GPCR ligands. As proof of concept, three case studies are presented.

## Materials and Methods

### Computational Profiling for GPCRs Alignment Tool

**FIGURE 1:**
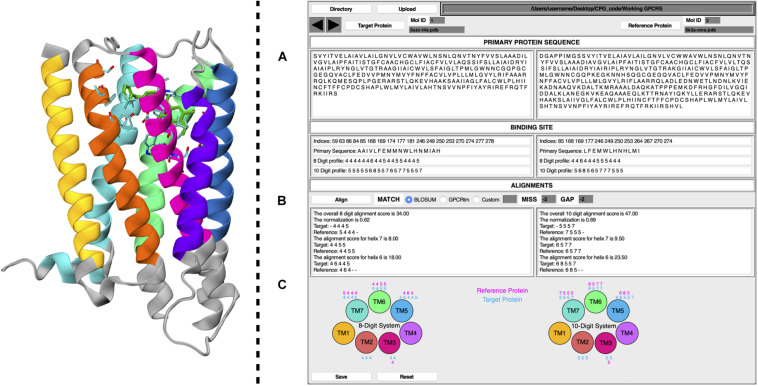
Schematic representation of CPG GUI. On the left, a GPCR is displayed as coded by CPG, where each TM helix is colored according to the following the scheme; TM1, yellow; TM2, orange; TM3, magenta; TM4, purple; TM5, blue; TM6, green; TM7, cyan. Residues unused for the profiling calculation have been colored in gray. The ligand has been colored in dark green, whereas its surrounding amino acids (cut-off 4 Å) are colored according to their atom types. On the right, the main window of the CPG is displayed, where **(A)** includes the primary protein sequence and binding site information, **(B)** the alignment results, and **(C)** the 8 and 10 Digit Profile scores assigned to the binding site residues for each helix of the target and reference proteins.

The CPG tool is a user-friendly GUI, written in the Tcl/Tk coding language. To improve its ease of use, the software has been released as a plug-in for VMD. CPG has been designed to extract information from PDB files of ligand–GPCR binary complexes. Details of the CPG tool are reported below, where points (A), (B), and (C) refer to the labels given in Figure 1.

(A) By taking the primary protein sequence and identifying the ligand-interacting residues (in a range of 4 Å from the ligand), information regarding the binding site of the protein is obtained. The binding site information generated by the CPG tool includes which residues are part of the binding site and their positions in the GPCR primary sequence (in the form of their resID number). A fundamental feature of CPG is its ability to convert the aforementioned data into a protein profile. That is to say, the residue list of the protein can be mutated into two available profiling systems, i.e., the “8 Digit Profile” (8DP) and the “10 Digit Profile” (10DP). Both are based on the physio-chemical properties of the amino acids, grouping them following different approaches. In the 8DP system, the amino acids are divided into four groups, i.e., “hydrophobic,” “hydrophilic,” “negatively charged,” and “positively charged,” to which we assigned an integer number (0, 1, 2, and 3, respectively). Consequently, we obtained a 1D array representing the GPCR primary sequence. At the same time, the integer number is increased by a value of 4 for the residues involved in the ligand-binding site (4 for the hydrophobic group, 5 for the hydrophilic, 6 for negatively charged, and 7 for positively charged). As a result, by exploiting only eight symbols, we can easily distinguish the ligand-interacting amino acids and their physio-chemical properties from the rest of the residues not interacting with the ligand. The 10DP system follows a similar fashion; however, we further split the hydrophobic group into “aliphatic” and “aromatic” subgroups, resulting in values ranging from 0 to 4 and 5 to 9 for general and binding site residues, respectively.
(B) The second important property of CPG is its ability to map the protein profile onto the GPCR topology, dividing it according to which helix of the GPCR each profiled residue belongs to. The data can be generated for a “target” and “reference” protein as chosen by the user, obtaining seven 1D arrays for each macromolecule. Once the desired pair of proteins has been selected, a local pairwise Levenshtein algorithm-based alignment is performed, in order to find the best matches between each corresponding helix. As shown in Figure 1, it is possible to choose different alignment scoring methods employing values extracted from the BLOSUM62 (Pietrokovski et al., 1996; Choudhuri, 2014) or the GPCRtm (Rios et al., 2015) substitution matrix, both of which have been adapted for the 8DP and 10DP groups. Furthermore, user-defined custom values may also be employed. Finally, the “MISS” and the “GAP” fields should be filled with non-positive values.
  Alignments based on 8DP and 10DP can be visualized in Figure 1B, where three different outputs are reported for both the target and the reference proteins. In detail, it displays the alignment score for each helix, the total score based on the sum of the scores of the individual helices, and, lastly, a normalized score which is expressed as:

N=T/R

where *T* is the target protein total score (the alignment score with the target protein against the reference protein), and *R* is the reference protein total score (a self-alignment score value of the reference protein). The normalization of the alignment score is important inasmuch as it considers the length of the aligned strings, thus taking into account different volumes in the binding pocket occupied by the ligands. The value of the normalized alignment score of the pairwise alignment between the target and the reference GPCR indicates a likelihood of repositioning one or both ligands in the reciprocal receptors.
(C) As described in point (B), the profiled binding site residues are divided according to each helix. The CPG tool provides a graphical visualization of the 8DP and 10DP scores attributed to each of the binding site residues of the 7 α-helices of the target and reference GPCRs.

A more detailed explanation of the CGP methodology including the alignment procedure and the scoring functions is provided in Supplementary Information, where we also report a tutorial for the use of CPG.

### Docking Calculation

We investigated the binding of ligands to repurposed GPCRs by means of molecular docking calculations. This computational technique is widely used to elucidate the ligand-binding mode in various molecular targets, including GPCRs (Anzini et al., 2008, 2011; Nuti et al., 2010; Limongelli, 2020). In particular, we performed cross-docking calculations by docking two ligands in their reciprocal receptor. These calculations were performed on selected pairs of ligand–GPCR complexes that have a CPG score higher than 0.5 and involve seemly pharmacologically unrelated GPCRs.

Molecular docking calculations were carried out using the AutoDock4.2.6 software package (AD4, Morris et al., 2009; Forli et al., 2016). Protonation states of protein residues and ligands were set at pH 7.0. Ligand and receptor structures were prepared and converted to AutoDock format files using AutoDockTools, and the Gesteiger-Marsili partial charges were then assigned. Grid points of 40 × 40 × 40 with a 0.375 Å spacing were calculated around the binding cavity using AD4. Thus, 100 separate docking calculations were performed for each run. Each docking run consisted of 2.5 million energy evaluations using the Lamarckian genetic algorithm local search (GALS) method. Otherwise, default docking parameters were applied. Docking conformations were clustered on the basis of their RMSD (tolerance = 1.5 Å). The analysis on the best binding poses was performed employing the “Drug Discovery Tool” (DDT, Aureli et al., 2019), a GUI recently developed in our group that enables a fast, yet accurate analysis of the docking calculation.

## Results

The CPG algorithm is based on protein profiling, a powerful bioinformatics technique that applies a dimensionality reduction process in which multiple properties of amino acid sequences are described by a mono-dimensional information string. By exploiting such a representation, it is possible to perform fast alignments between diverse proteins based on the chemical similarities of their amino acids. In such a way, it is possible to employ a scoring method based on the conservation of protein residues. For the present study, we set up two scoring functions, namely, 8DP and 10DP, that exploit two well-known scoring matrices: (i) BLOSUM62 (Pietrokovski et al., 1996), which is a generalized scoring method for all proteins; and (ii) GPCRtm (Rios et al., 2015), which has been specifically developed for Class A GPCRs. In detail, we used CPG to generate alignment score tables based on the pairwise alignments of the 55 GPCR pdb files available in the PDB databank. Each score was computed by employing a specific scoring function, reporting a final normalized value. In particular, the 8DP algorithm converts each amino acid into an integer number, following the scheme hydrophobic = 0, hydrophilic = 1, negatively charged = 2, and positively charged = 3. CPG then discriminates the residues interacting with the ligand by increasing their numerical value by 4. 10DP follows a similar rationale, further dividing the hydrophobic group into two subgroups, “aliphatic” and “aromatic.” In 10DP, the profiling scheme is aliphatic = 0, aromatic = 1, hydrophilic = 2, negatively charged = 3, and positively charged = 4, while the score of the ligand-interacting residues is increased by 5. Exploiting two different profiling systems allows us to take into account the impact of π–π interactions, which is explicitly accounted for in the 10DP scheme (see “Materials and methods” section and Supplementary Information for details). A step-by-step tutorial to guide the reader in the use of CPG is provided in the Supplementary Information.

The scoring matrices reported in Supplementary Tables 1, 2 were employed to determine the likelihood of drug repositioning considering the alignment between two different ligand–GPCR complexes, where a threshold value of 0.5 for the normalized alignment score was considered. In particular, we found ∼600 complexes that fulfill this condition, most of them obtained from different pdb complexes of the same GPCR, as expected. However, ∼10% of the top-ranked hits regarded complexes of different GPCRs. Among these, three pairs of drug–GPCR complexes, for a total of six systems, were further investigated with the aim of assessing the CPG prediction. In detail, we evaluated the GPCR cross-activity of the drug by means of cross-docking calculations in the newly identified GPCR target and by analyzing the available data on its pharmacological activity. The investigated pairs of complexes are (i) the 5-hydroxytryptamine receptor 2B with the ligand alprenolol and the beta-2 adrenergic receptor with the ligand lisuride; (ii) the 5-hydroxytryptamine receptor 2B with the ligand timolol and the beta-2 adrenergic receptor with the ligand lysergic acid diethylamide (LSD); and (iii) the M2 muscarinic acetylcholine receptor with the ligand ICI-118,551 and the beta-2 adrenergic receptor with the ligand quinuclidinyl benzilate (QNB). The results are discussed in detail in the following paragraphs.

### Lisuride–5HT2B and Alprenolol–ADRB2

**FIGURE 2:**
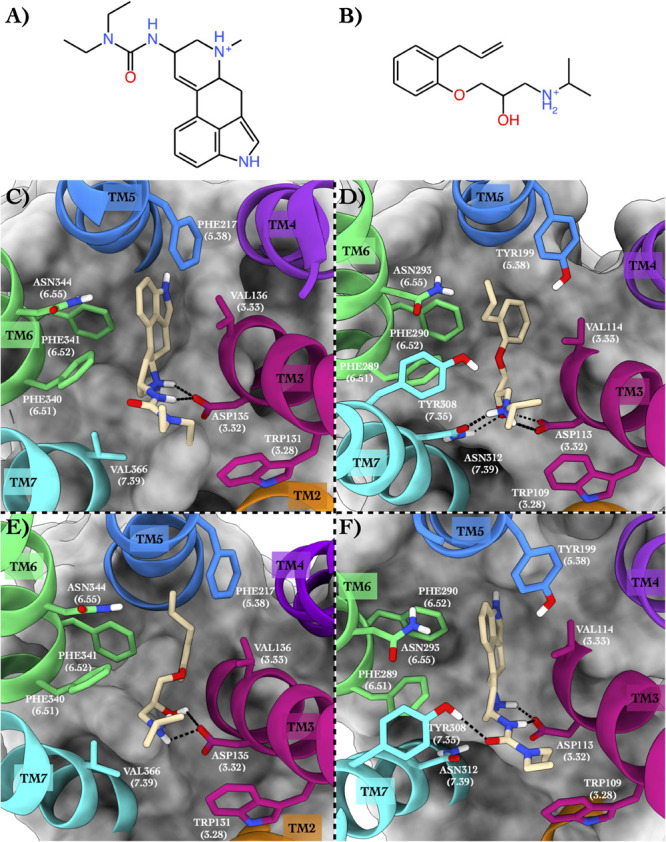
Schematic depictions of lisuride and alprenolol and their respective binding sites inside 5HT2B and ADRB2. **(A)** Chemical structure of lisuride at physiological pH. **(B)** Chemical structure of alprenolol at physiological pH. **(C)** Lisuride–5HT2B crystallographic binding mode. **(D)** Alprenolol–ADRB2 crystallographic binding mode. **(E)** Centroid of the most populated cluster family coming from the docking calculation between 5HT2B 3D structure and alprenolol. **(F)** Centroid of the most populated cluster family coming from the flexible docking calculation between ADRB2 and lisuride. Lisuride and alprenolol have been colored in tan. The surrounding residues are labeled using both primary sequence and Ballesteros–Weinstein numbering. The helices and the marked residues have been depicted according to the CGP color scheme, with TM2 in orange, TM3 in magenta, TM4 in purple, TM5 in blue, TM6 in green, and the TM7 in cyan.

The first case study regards the 5-hydroxytryptamine receptor 2B (also known as the serotonin receptor 2B, hereafter 5HT2B, Hensler, 2012) bound to lisuride (Figure 2A), one of its marketed antagonists, and the beta-2 adrenergic receptor (hereafter ADRB2, Rascol et al., 2007) in complex with its antagonist alprenolol (Figure 2B).

#### Binding Mode in the Native GPCR

In the x-ray structure of the complex 5HT2B–lisuride (PDB ID 6DRX, McCorvy et al., 2018), the ligand forms a salt bridge and a H-bond with Asp135, while its indole ring is placed in a pocket shaped by several hydrophobic/aromatic residues (Figure 2C). Here, the ligand engages π–π stacking interactions with Phe217, Phe340, and Phe341, and van der Waals interactions with Val136 and Val366. Finally, the indole ring of the lisuride can also form a π-mediated H-bond with Asn344. In the second complex formed by ADRB2 bound to alprenolol (PDB ID 3NYA, Wacker et al., 2010), protonated amine function of the ligand H-bonds with Asn312, also engaging a salt bridge interaction with Asp113 (Figure 2D). In addition, the hydroxy group of the ligand forms H-bonds with Asp113 and Asn312. On the contrary, the aromatic ring of the alprenolol interacts with Val114, Tyr199, Phe289, and Phe290 through π–π stacking and van der Waals interactions. Finally, the ortho-allyl group of the alprenolol is placed in a competent position to form a π-mediated electron transfer interaction with Asn293.

Employing CPG, we found that the lisuride–5HT2B and alprenolol–ADRB2 complexes show a 10DP alignment score of 0.54, higher than the threshold value 0.5 (Table 1), suggesting that lisuride and alprenolol could be repurposed as novel ligands for their reciprocal GPCRs.

**TABLE 1:** The alignment scores computed for the PDB sequence 6DRX and 3NYA using a miss and gap score of −2.

Target protein	Reference protein	8DP BLOSUM min:−0.84 max: 0.73	8DP GPCRtm min:−0.83 max: 0.75	10DP BLOSUM min:−0.77 max: 0.66	10P GPCRtm min: −0.97 max: 0.66
5HT2B/H8G (PDB ID: 6DRX)	ADRB2/JTZ (PDB ID: 3NYA)	0.39	0.4	0.54	0.46
ADRB2/JTZ (PDB ID: 3NYA)	5HT2B/H8G (PDB ID: 6DRX)	0.36	0.37	0.52	0.43

*The highest and lowest values for each profiling scoring function obtained by aligning all the diverse GPCRs available in the PDB databank are also reported.*

#### Binding Mode in the Repurposed GPCR

In order to assess the CPG prediction and validate this hypothesis, we performed cross-docking calculations in which lisuride was studied in the ADRB2 structure, while alprenolol was studied in the 5HT2B structure (see “Materials and methods” section for docking details). The docking results confirmed the ability of these two ligands to cross-bind their reciprocal GPCR, showing binding modes stabilized by a series of favorable interactions (see Figure 2E and Table 2). In particular, in the most populated binding pose of alprenolol in 5HT2B, the ligand forms a salt bridge interaction with Asp135, which resembles that established with Asp113 in ADRB2. An additional H-bond is formed between the hydroxyl group of the alprenolol and Asp113, while the isopropyl moiety of the ligand engages hydrophobic contacts with Val366 and Trp131. On the contrary, the aromatic ring of the alprenolol is located in a hydrophobic pocket remarkably similar to that present in ADRB2 (Figures 2D,E). Here, the ligand forms π–π stacking interactions with Phe217, Phe340, and Phe341, and van der Waals contact with Val136. In addition, the allyl π-electrons of the alprenolol are involved in electron transfer interaction with Asn344 as similarly engaged with Asn293 in ADRB2. In the case of the binding of lisuride in ADRB2, considering the bulkiness of the ligand we performed a flexible docking calculation to allow conformational flexibility of the Asp113 side chain.

**TABLE 2:** The cross-docking calculation scores of the 5HT2B receptor with alprenolol and the ADRB2 receptor with lisuride.

Protein	Ligand	Mean binding energy (docking score)	Runs in cluster	Number of clusters
5HT2B	JTZ	−6.58	62/100	3
ADRB2	H8G	−10.78	100/100	1

In the most populated binding pose, the ligand forms strong interactions with the receptor like the salt bridge and the H-bond with Asp113, reproducing the same interactions established with Asp135 in 5HT2B. A further H-bond formed by the ligand’s urea oxygen with Tyr308 stabilizes the binding mode. In addition, while the two ethyl groups form hydrophobic contacts with Trp109, the aromatic moiety engages π-π stacking and Van der Waals interactions with Tyr199, Phe289, Phe290, and Val114. Finally, the indole ring of the lisuride forms a π-mediated H-bond with Asn293 as similarly done with Asn344 in ADRB2. A detailed list of the interactions established by lisuride and alprenolol with 5HT2B and ADRB2 is reported in Supplementary Table 4.

#### Lisuride and Alprenolol Pharmacology

In order to assess the repurposing of lisuride and alprenolol as ligands of ADRB2 and 5HT2B, respectively, we thoroughly studied their pharmacological profiles. Lisuride is an ergot derivative, administered for the treatment of Parkinson’s disease, depression, and migraines (Gopinathan et al., 1981; Egan et al., 1998; Hofmann et al., 2006). The mechanism of action of lisuride is due to its agonist activity on several serotonin receptor subtypes (5HT1A, 5HT1B, 5HT1D, 5HT2A, 5HT2B, and 5HT2C) (Egan et al., 1998), as well as on the dopamine receptors D1, D2, D3, D4, and D5 (Hildebrand et al., 1987). It should be underlined that lisuride has already undergone a drug repositioning process where it has been repurposed for the suppression of lactation as it lowers serum prolactin levels (Van Dam and Rolland, 1981).

Alprenolol is a beta-adrenergic antagonist with anti-arrhythmic effects, being able to bind ADRB1, ADRB2, and ADRB3 (Himori et al., 1977). The activity of alprenolol is given by the inhibition of the activity of the beta-adrenergic receptor’s natural ligands epinephrine and norepinephrine. As a consequence, alprenolol induces a reduction in heart rate (Wasserman et al., 1970). Alprenolol also has an anti-hypertensive effect by inhibiting the production of renin, thus acting on the renin–angiotensin–aldosterone system (RAAS) by lowering angiotensin II and aldosterone production, which leads to the reduction of vasoconstriction and water retention (Himori et al., 1977). While it has been reported that alprenolol can also bind to the 5HT1A receptor, so far there is no evidence that it is also able to bind the 5HT2B receptor. In particular, the pharmacological activity of alprenolol on the 5-hydroxytryptamine (5-HT)-induced hyperactivity response has been studied as early as 1978 (Costain and Green, 1978); however, the spectrum of its molecular targets is still unexplored. The activity of alprenolol toward 5HT2B might explain the relevant side effects of this drug, such as the gastrointestinal ones (Amjad et al., 2017). This might be due to the presence of adrenergic receptors in the gastrointestinal tract, as well as 5HT2B, which is a ubiquitous GPCR also expressed in the liver and the intestine (Papadimas et al., 2012). On the contrary, 5HT1A is poorly expressed in the gastrointestinal tract, being mostly located on the lymph nodes, the thymus, and the spleen. Elucidating the different GPCRs targeted by alprenolol might lead to a better understanding of the adsorption of the body and the toxicity of this drug. To this end, the results of our study highlight a potential activity of alprenolol on 5HT2B and lisuride on ADRB2, suggesting to further investigate the molecular interaction of these drugs with the two receptors with the scope to rationalize toxicity and propose novel, repurposed clinical applications for these two drugs.

### Lysergic Acid Diethylamide–5HT2B and Timolol–ADRB2

**FIGURE 3:**
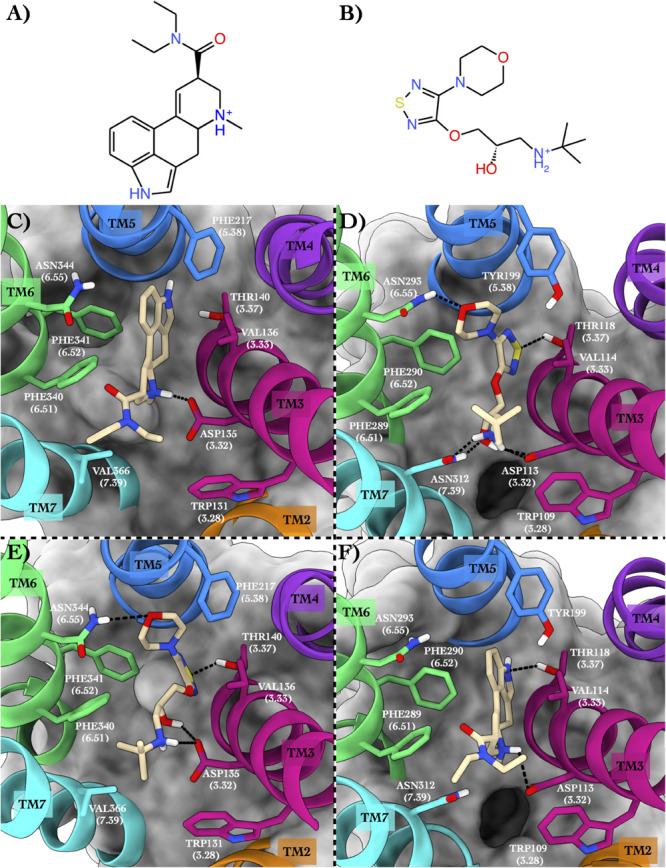
Schematic depictions of LSD and timolol and their binding modes inside the 5HT2B and ADRB2 receptors. **(A)** Chemical structure of LSD at physiological pH. **(B)** Chemical structure of timolol at physiological pH. **(C)** LSD–5HT2B crystallographic binding mode. **(D)** Timolol–ADRB2 crystallographic binding mode. **(E)** Centroid of the most populated cluster family coming from the docking calculation between 5HT2B and timolol. **(F)** Centroid of the most populated cluster family coming from the docking calculation of the ADRB2 3D structure and LSD. LSD and timolol have been colored in tan. The surrounding residues are labeled using both primary sequence and Ballesteros–Weinstein numbering. The helices and the marked residues have been depicted according to the CGP color scheme, with TM2 in orange, TM3 in magenta, TM4 in purple, TM5 in blue, TM6 in green, and TM7 in cyan.

The second case study regards 5HT2B and ADRB2 in complex with lysergic acid diethylamide (hereafter LSD) (Figure 3A) and timolol (Figure 3B), respectively.

#### Binding Mode in the Native GPCR

The 5HT2B x-ray structure (PDB ID 5TVN, Wacker et al., 2017) in complex with LSD (Figure 3C) shows a salt bridge interaction between the charged amine of the LSD and Asp135 and π–π stacking–hydrophobic interactions between the aromatic moiety of the ligand and Phe217, Phe340, Phe341, and Val136. The diethylamide function of the LSD forms additional van der Waals contacts with Trp131 and Val366 that further stabilize the binding pose. The second system is ADRB2 in complex with timolol (PDB ID 3D4S, Hanson et al., 2008; Figure 3D). Here, a network of H-bonds stabilizes the binding mode. In detail, the sulphur atom of the timolol’s thiadiazole H-bonds with Thr118, while the oxygen of timolol’s morpholine ring engages a H-bond with Asn293. On the contrary, the protonated amine group of the LSD forms a salt bridge interaction with Asp113 and a H-bond with Asn312. The same residues also establish H-bonds with the hydroxyl group of the ligand. Finally, π–π stacking and hydrophobic interactions are made by the thiadiazole moiety of the timolol and the terminal tert-butyl group with Phe290, Phe289, and Trp109.

The above two systems have high CPG alignment scores, especially in the case of the 8DP scoring function (Table 3). This scoring function weighs the hydrophilic interactions between the ligand and the GPCR more than the 10DP one, thus assigning a higher score to binding modes characterized by polar contacts—H-bonds and salt bridges—like those present in these two complexes. In order to assess the CPG prediction of cross-affinity of LSD and timolol in their reciprocal GPCR, cross-docking calculations of timolol in 5HT2B and LSD in ADRB2 were performed, and the results are discussed as follows (Table 4).

**TABLE 3:** The alignment scores computed for the PDB sequence 5TVN and 3D4S using a miss and gap score of −2.

Target protein	Reference protein	8DP BLOSUM min:−0.84 max: 0.73	8DP GPCRtm min:−0.83 max: 0.75	10DP BLOSUM min:−0.77 max: 0.66	10P GPCRtm min: −0.97 max: 0.66
5HT2B/7LD (PDB ID: 5TVN)	ADRB2/TIM (PDB ID: 3D4S)	0.63	0.64	0.47	0.43
ADRB2/TIM (PDB ID: 3D4S)	5HT2B/7LD (PDB ID: 5TVN)	0.61	0.61	0.52	0.43

*The highest and lowest values for each profiling scoring function obtained by aligning all the diverse GPCRs available in the PDB databank are also reported.*

**TABLE 4:** The cross-docking calculation scores of the 5HT2B receptor with timolol and the ADRB2 receptor with LSD.

Protein	Ligand	Mean binding energy (docking score)	Runs in cluster	Number of clusters
5HT2B	TIM	−7.96	58/100	5
ADRB2	7LD	−10.55	100/100	1

#### Binding Mode in the Repurposed GPCR

In the most recurring docking pose of timolol in 5HT2B, the charged amine of the ligand forms a salt bridge with Asp135 as similarly done with Asp113 in ADRB2 (Figure 3E). Three additional H-bonds further stabilize the timolol binding mode such as those formed by its hydroxyl group with Asp135, its morpholine ring with Asn344, and its thiadiazole sulfur atom with Thr140. Finally, π–π stacking and hydrophobic interactions are formed by the thiadiazole moiety with Phe340 and by the terminal tert-butyl group of the ligand with Phe341 and Val366, respectively.

In Figure 3F, we show the cross-docking result of LSD in ADRB2. Here, the ligand occupies the binding pocket establishing π–π stacking and van der Waals interactions with the surrounding residues Phe290 and Val114. The anchor point of the ligand binding is the typical salt bridge made by the charged amine of the LSD with Asp113, whereas the amine of the indole ring of the ligand can form a H-bond with Thr118. Finally, hydrophobic contacts are engaged by the diethylamide group of the ligand with Trp109 and Phe289. It is worth noting that most of these interactions are also present in the timolol-binding mode, showing remarkable strength and similarity in the interaction with ADRB2 for these two drugs. This finding fully agrees with the high binding affinity of LSD to ADRB2 resulted from the docking calculations and reported in Table 4. As before, we report the full list of the interactions formed by LSD and timolol with 5HT2B and ADRB2 in Supplementary Table 5.

#### Timolol Pharmacology

When evaluating the possibility of repositioning timolol, it should be noted that timolol is a drug used as eye drops that targets the beta-1 and beta-2 adrenergic receptors which results in a decrease in eye pressure (e.g., caused by glaucoma, Sambhara and Aref, 2014). Furthermore, timolol has also been used for the treatment of hypertension. From a pharmacological point of view, timolol is an antagonist for the beta-adrenergic receptor. One of the most common side effects of timolol is the onset of depression; however, the understanding of such a side effect is yet unknown (Nolan, 1982). Prompted by the CPG results and supported by the cross-docking calculations, we propose timolol as the ligand of the serotonin receptor 5-HT2B similar to LSD. The activity of timolol on 5HT2B, working as off-target, might explain the neurological disorders caused by the use of this drug. This represents an example of how to use CPG in the investigation of drug side effects by evaluating drug off-target activity through its repositioning toward a novel GPCR. This step is valuable, especially in the early stages of drug development, to assess whether the newly designed drug can bind off-targets that might cause undesirable side effects.

### Quinuclidinyl Benzilate–ACM2 and ICI-118,551–ADRB2

**FIGURE 4:**
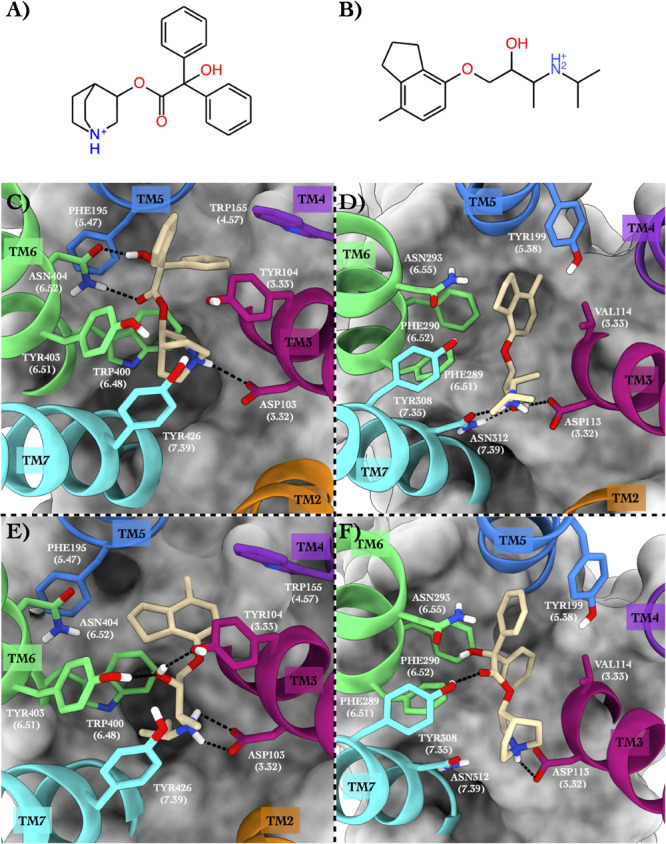
Schematic depictions of the chemical structures of quinuclidinyl benzilate and ICI-118,551 and their binding modes inside the ACM2 and ADRB2 receptors, respectively. **(A)** Chemical structure of quinuclidinyl benzilate at physiological pH. **(B)** Chemical structure of ICI-118,551 at physiological pH. **(C)** Quinuclidinyl benzilate–ACM2 crystallographic binding mode. **(D)** ICI-118,551–ADRB2 crystallographic binding mode. **(E)** Centroid of the most populated cluster family coming from the docking calculation of ICI-118,551 in the ACM2 receptor. **(F)** Centroid of the most populated cluster family coming from the docking calculation between ADRB2 and quinuclidinyl benzilate. Quinuclidinyl benzilate and ICI-118,551 have been colored in tan. The surrounding residues are labeled using both primary sequence and Ballesteros–Weinstein numbering. The helices and the marked residues have been depicted according to the CGP color scheme, with TM2 in orange, TM3 in magenta, TM4 in purple, TM5 in blue, TM6 in green, and TM7 in cyan.

As the third case study, we investigated the M2 muscarinic acetylcholine receptor (hereafter ACM2) bound to the antagonist quinuclidinyl benzilate (QNB, Figure 4A, Shirakawa et al., 1987) and the ADRB2 receptor in complex with the antagonist ICI-118,551 (JRZ, Figure 4B; see Table 5).

**TABLE 5:** The alignment scores computed for the PDB sequence 3UON against 3NY8 using a miss and gap score of −2.

Target protein	Reference protein	8DP BLOSUM min:−0.84 max: 0.73	8DP GPCRtm min:−0.83 max: 0.75	10DP BLOSUM min:−0.77 max: 0.66	10P GPCRtm min: −0.97 max: 0.66
ACM2/QNB (PDB ID: 3UON)	ADRB2/JRZ (PDB ID: 3NY8)	0.61	0.62	0.59	0.54
ADRB2/JRZ (PDB ID: 3NY8)	ACM2/QNB (PDB ID: 3UON)	0.47	0.49	0.43	0.42

*The highest and lowest values for each profiling scoring function obtained by aligning all the diverse GPCRs available in the PDB databank are also reported.*

#### Binding Mode in the Native GPCR

In the x-ray structure of the QNB–ACM2 complex (PDB ID 3UON, Haga et al., 2012; Figure 4C), the ligand engages a salt bridge through the charged amine group with Asp103, whereas its carbonyl and hydroxyl groups form two H-bonds with Asn404. The azabicyclooctan moiety of the ligand is placed in a hydrophobic pocket surrounded by aromatic residues like Trp400, Tyr403, and Tyr426, while one of its two aromatic rings forms π–π stacking interactions with Tyr104 and Trp155. In the crystallographic ADRB2 in complex with JRZ (PDB ID 3NY8, Wacker et al., 2010; Figure 4D), the charged amine and hydroxyl groups of the ligand form three H-bonds with Asn312 and Asp113, while the indanyl moiety engages hydrophobic interactions with Val114, Tyr199, and Phe290. The high CPG alignment score for the above two drug–GPCR complexes prompted us to further assess through docking calculations the capability of JRZ and QNB to interact with their reciprocal receptors ACM2 and ADRB2, respectively (Table 5).

#### Binding Mode in the Repurposed GPCR

The ligand JRZ shows a strong affinity for ACM2 with a remarkable docking score of −9.43 for the most populated binding mode (Table 6). In this pose (Figure 4E), the charged amine group of JRZ forms a salt bridge with Asp103, mimicking that made by QNB (Figure 4C). The hydroxyl group of the ligand engages two H-bonds with Tyr104 and Tyr403, while the indanyl moiety establishes π–π interactions with aromatic residues like Trp155, Phe195, and Trp400.

**TABLE 6:** The cross-docking calculation scores of the ACM2 receptor with JRZ and the ADRB2 receptor with QNB.

Protein	Ligand	Mean binding energy (docking score)	Runs in cluster	Number of clusters
ACM2	JRZ	−9.43	50/100	3
ADRB2	QNB	−9.91	72/100	3

Regarding QNB in ADRB2 (Figure 4F), docking calculations show a strong interaction between the ligand and this GPCR with a low docking score (Table 6). The best and most populated docking pose shows the ligand interacting with the typical salt bridge interaction with Asp113, as seen in the case of the JRZ–ADRB2 binary complex (Figure 4D). In addition, while the ligand interaction with Asn312 is lost if compared with JRZ, a new H-bond is formed between the hydroxyl group of the QNB and Tyr308. Interestingly, the two aromatic rings of QNB contribute to further stabilize the binding mode through hydrophobic and π–π stacking interactions with Val114, Tyr199, Phe289, Phe290, and *via* a π-mediated H-bond with Asn293. As done for the previously discussed systems, the full list of the interactions established by QNB and JRZ with ACM2 and ADRB2 is reported in Supplementary Table 6.

#### ICI-118,551 Pharmacology

ICI-118,551 is an ADRB2 antagonist widely used in research for its 100-fold higher selective inhibition of ADRB2 with respect to ADRB1 and ADRB3. A recent work (Kashihara et al., 2014) has reported that administrating to mice an ADRB agonist, isoproterenol, together with ICI-118,551 gives similar pharmacological effects compared to mice administrated with a combination of isoproterenol and atropine, a well-known ACM2 antagonist. The authors explained this finding based on the common intracellular pathways shared by adrenergic and cholinergic signaling. However, our results pave the way to a new hypothesis that the similar pharmacological outcome of ICI-118,551 and atropine might be due to their common affinity toward the ACM2 receptor, an intriguing perspective that is worthy of further investigations.

## Discussion

The pharmacological relevance of GPCRs is highlighted by the fact that almost 40% of prescribed drugs target this receptor family (Rask-Andersen et al., 2011). The structural conservation in these membrane proteins allows for the relatively systematic profiling of their binding sites because the helices of GPCRs form a “barrel” structure composed of seven helices connecting the extracellular and intracellular spaces. The GPCRs do this by binding to a variety of ligands (small molecules, peptides, and even other proteins), which can be either exogenous or endogenous. A profiling methodology called Computational Profiling GPCRs (CPG) has been proposed here, which combines the primary structure of a GPCR with three-dimensional (3D) information when the receptor is complexed with a ligand, thus making the extraction of valuable data relating to the ligand–GPCR binding affinity possible. In particular, by converting the protein sequence into a 1D string of values representing the chemico-physical properties of the amino acids and the ligand–receptor binding interactions, a pairwise alignment of the GPCR-binding sites can be done in a simplified manner. A proper alignment driven by scoring methods based on the conservation of protein residues enables the detection of possible drug repositioning with important consequences in our understanding of drug pharmacology and side effects.

The profiling and aligning of ligand–GPCRs complexes were carried out using CPG on the available crystal structures. Our results show that there are promiscuous ligands that might be able to bind different GPCRs. As proof of concept, we have reported and discussed in detail three case studies that are: (i) lisuride–5HT2B and alprenolol–ADRB2; (ii) LSD–5HT2B and timolol–ADRB2; and (iii) quinuclidinyl benzilate–ACM2 and ICI-118,551–ADRB2. The CPG algorithm reported these systems among the top-scored ones, thus suggesting the repurposing of these drugs for their reciprocal receptor. We validated the CPG results by molecular docking calculations and provided a pharmacological basis with the data available in the literature. We showed that CPG can be useful to propose novel, repurposed clinical applications for the investigated drugs and for the rationalization of drug side effects by evaluating their off-target activity through repositioning toward a novel GPCR. The latter is a valuable process, especially in the early stages of drug development, to assess whether the newly designed drug can bind off-targets that might cause undesirable side effects. Certainly, further investigations, for instance, using binding free-energy calculations (Limongelli et al., 2013; Comitani et al., 2016; Moraca et al., 2017; Brotzakis et al., 2018; Yuan et al., 2018; D’Annessa et al., 2019; Raniolo and Limongelli, 2020) and *in vitro* experiments are necessary to properly assess the binding affinity and the pharmacological activities of the investigated ligands. Particularly, in GPCRs where ligand binding involves parts of the receptor endowed with conformational flexibility like the extracellular loops, molecular-binding simulations should be performed using methodologies as molecular dynamics that are more efficacious than docking in taking into account receptor flexibility and ligand-induced fit effects, thus providing a reliable validation of the CPG predictions. We point out that when preparing the ligand–GPCR complex for the validation simulations, receptor and ligand properties like the protonation state of specific residues or ligand functional groups, might be not immediately apparent from the sequence and structural data and need to be carefully considered by the investigator.

We note that CPG results rely on the type and quality of input data including the class of the GPCR, the chemical structure of the ligand, the similarity of the ligand-binding site, and the resolution of the ligand–GPCR complex structure. In this regard, one might observe that aminergic class A GPCRs are typically reported among the top-scored systems. This finding is not surprising considering that they are the most representative GPCR subgroup in the PDB databank, which is used as data source of ligand–GPCR complexes in CPG. Furthermore, one of the substitution matrices used in our study, GPCRtm, was developed based on sequences of class A GPCRs, thus performing better in scoring alignments of this GPCR subgroup. However, CPG is designed to work with any GPCR, and we expect that it will provide useful results even for GPCRs of the other classes as more receptor structures will be resolved, and alignment scoring functions optimized for the other classes of GPCRs will be available.

In addition, there is still room for improvement of the methodology. Namely, due to the employment of aligning procedures based only on generalized physio-chemical properties, the spatial information on the ligand–receptor interaction is lost as well as the binding cavity accessible volume. This means that in some instances, the alignment score may seem promising; however, the residues at the binding site might not be in a proper position to allow ligand binding. In such a case, a practical solution is to compute how the alignment score changes as a function of the gap penalty applied. Based on our experience, the less the score changes using different gap penalty values, the more the size of the ligands under examination are similar. Therefore, by looking at the global alignment of all the helices, not merely at each individual helix separately, a greater understanding of binding site similarities is achieved. This procedure can improve the accuracy and the specificity of the methodology (fewer false positives).

## Conclusion

In conclusion, CPG has proved to be an appealing tool to rapidly investigate drug repurposing for GPCRs. Our tool performs particularly well with aminergic class A GPCRs since they are the most representative GPCR structures in the PDB databank, and they were also employed to develop one of the alignment scoring functions used in our study. We report the full list of GPCRs repurposed ligand candidates identified in our study in Supplementary Table 3. This represents a useful data source for investigations on the pharmacological activities of these compounds. A future extension of our methodology, including profiling of binding sites for apo GPCRs, is desirable as it would pave the way for applications of CPG not only in GPCR drug repurposing but also in *de novo* drug discovery pipelines. More than 800 GPCRs have been identified by sequence analysis on the human genome; however, only a comparatively low number of them have been targeted (Sriram and Insel, 2018). Due to their pharmacological relevance, there is clearly the urgency of finding methods that are able to speed up the identification of lead compounds, which then can finally undergo a lead optimization process. In addition, having a reliable dimensionality-reduced description of the drug–GPCR molecular interaction, especially in 1D string, represents a precious tool in the employment of machine learning approaches in drug development as expected in the near future. Our CPG is a promising methodology that points exactly in this direction.

## Data Availability Statement

The code of CPG is available at this link https://sites.google.com/site/vittoriolimongelli/downloads, together with a directory containing the GPCRs employed in the present manuscript. An easy-to-use tutorial has been reported in Supplementary Material.

## Author Contributions

VL designed the research. SA and VL conceived the algorithm. AD and SA wrote the code and performed the docking calculations. AD, SA, and VL analyzed the results and wrote the manuscript. All authors contributed to the article and approved the submitted version.

## Conflict of Interest

The authors declare that the research was conducted in the absence of any commercial or financial relationships that could be construed as a potential conflict of interest.
