# Supplementary material for: Drug Repurposing on G Protein-Coupled Receptors Using a Computational Profiling Approach
Source: Front Mol Biosci. 2021 May 7;8:673053. doi: 10.3389/fmolb.2021.673053 (PMC8138314; doi:10.3389/fmolb.2021.673053)
Supplement: Supplementary file 1 [file Data_Sheet_1.PDF]

SUPPLEMENTARY MATERIAL

The supplementary material is composed by the following sections:

- 8 and 10 Digit profiles
- Scoring Matrices
- Alignment Matrices
- Most promising CPG alignments
- Supplementary results tables
- CPG user guide

8 AND 10 DIGIT PROFILES

We begin with a description of the protein profiling used in the CPG tool. The profiling was based on the transformation of the 20 proteinogenic residues, that the primary sequence of a protein is composed of, into two profiles containing 8 and 10 digits (8DP and 10DP, respectively). The digits of these profiles cluster the protein's amino acids according to the physico-chemical properties that they possess. In this way, the protein primary structure is simplified to allow for less stringent conditions for the alignment. Profiling the protein was also used as a means to distinguish the binding site of the protein from the rest of its primary sequence. The protein profile can be seen in 2 parts. The amino acids that are not involved in binding are given a value from 0-3 or 0-4 for 8DP and 10DP, respectively. The amino acids that are involved in ligand-protein binding interactions are given a value from 4-7 and 5-9 for 8DP and 10DP, respectively. To identify which amino acids are in the binding site, the VMD *atomselect* command *"(same residue as within 4 of (not protein))*" was used, which identifies any protein residue that is within 4 Å of the ligand. To understand the profiling more

Profile value	Residues	Residue Property	Binding site Residue Value
0	PRO, VAL, ALA, GLY, LEU, ILE, PHE, MET, TYR, TRP	Hydrophobic	4
1	SER, ASN, THR, CYS, GLN, HIS	Hydrophilic Uncharged	5
2	GLU, ASP	Negatively Charged	6
3	ARG, LYS	Positively Charged	7

Table S1: Table with 8 Digit Profile System Values

Profile value	Residues	Residue Property	Binding site Residue Value
0	PRO, VAL, ALA, GLY, LEU, ILE, MET	Hydrophobic Aliphatic	5
1	PHE, TYR, TRP	Hydrophobic Aromatic	6
2	SER, ASN, THR, CYS, GLN, HIS	Hydrophilic Uncharged	7
3	GLU, ASP	Negatively Charged	8
4	ARG, LYS	Positively Charged	9

Table S2: Table with 10 Digit Profile System Values

clearly, please refer to Tab. S1 for the 8DP and Tab. S2 for the 10DP. By assigning a unique value to the binding site residues, it is simpler to extract and use only these residues for the alignment with other GPCRs.

SCORING MATRICES

In order to generate an effective scoring function for CPG, match scores derived from the BLOSUM62 and GPCRtm scoring matrices were established for the 8DP and 10DP systems. Descriptions of the matrices, the rationale behind their implementation, and how they were integrated into CPG are presented below.

BLOSUM62

BLOSUM (blocks substitution matrices) scoring matrices are long-odds matrices that are used for scoring of sequence alignments. They have been integrated into a variety of sequence alignment algorithms that have been used for genomic and proteomic sequence alignments in a number of bioinformatics analysis tools. BLOSUM matrices are based on the conserved amino acid sequences of 500 related protein sequences.[1] These scoring matrices were developed in 1992 by using the BLOCKS alignment database.[2] The BLOCKS alignment database carried out alignments of multiple sequences which generated clusters of sequences that had a relatively high similarity percentage,

BLOSUM62

**A**	4																			
**R**	-1	5																		
**N**	-2	0	6																	
**D**	-2	-2	1	6																
**C**	0	-3	-3	-3	9															
**Q**	-1	1	0	0	-3	5														
**E**	-1	0	0	2	-4	2	5													
**G**	0	-2	0	-1	-3	-2	-2	6												
**H**	-2	0	1	-1	-3	0	0	-2	8											
**I**	-1	-3	-3	-3	-1	-3	-3	-4	-3	4										
**L**	-1	-2	-3	-4	-1	-2	-3	-4	-3	2	4									
**K**	-1	2	0	-1	-3	1	1	-2	-1	-3	-2	5								
**M**	-1	-1	-2	-3	-1	0	-2	-3	-2	1	2	-1	5							
**F**	-2	-3	-3	-3	-2	-3	-3	-3	-1	0	0	-3	0	6						
**P**	-1	-2	-2	-1	-3	-1	-1	-2	-2	-3	-3	-1	-2	-4	7					
**S**	1	-1	1	0	-1	0	0	0	-1	-2	-2	0	-1	-2	-1	4				
**T**	0	-1	0	-1	-1	-1	-1	-2	-2	-1	-1	-1	-1	-2	-1	1	5			
**W**	-3	-3	-4	-4	-2	-2	-3	-2	-2	-3	-2	-3	-1	1	-4	-3	-2	11		
**Y**	-2	-2	-2	-3	-2	-1	-2	-3	2	-1	-1	-2	-1	3	-3	-2	-2	2	7	
**V**	0	-3	-3	-3	-1	-2	-2	-3	-3	3	1	-2	1	-1	-2	-2	0	-3	-1	4
	**A**	**R**	**N**	**D**	**C**	**Q**	**E**	**G**	**H**	**I**	**L**	**K**	**M**	**F**	**P**	**S**	**T**	**W**	**Y**	**V**

Figure S1: The Blosum62 Matrix which shows the scoring values for the 20 proteinogenic amino acids (represented with their 1-letter code) according to their 62% similarity score.

which was then averaged. By clustering the various sequences, the substitution frequencies of the 20 amino acids could be determined for development of the BLOSUM matrices by looking at blocks of ungapped multiple sequence alignments by exploiting the fact that proteins are evolutionarily related. There are several BLOSUM matrices which have been derived based on the percentage of sequence similarities used to define a cluster and determine the blocks. The percentage similarity is indicated in the name of the matrix with a number and in the case of CPG, the BLOSUM62 matrix was used. For CPG, this matrix was chosen because of the low similarities between GPRC primary sequences.[1] Since BLOSUM matrices score alignments between evolutionarily divergent protein sequences, the likelihood of their presence determines how valuable the residue is. BLOSUM looks at the conserved regions of the protein and the relative frequencies of residues with their substitution probabilities. Figure S1 shows the BLOSUM62 scoring matrix and its values.

A crucial step was assigning a match score to each residues' class. To integrate the BLOSUM62 matrix within CPG, the BLOSUM62 matrix was first normalized so that the minimum score was equal to 0 and it was then divided into 4 and 5 smaller matrices for the 8 and 10 Digit profiling systems, respectively (Fig. S2 & S3).

4 - HYDROPHOBIC

	A	G	I	L	M	F	P	W	Y	V
A	8									
G	4	10								
I	3	0	8							
L	3	0	6	8						
M	3	1	5	6	9					
F	2	1	4	4	4	10				
P	3	2	1	1	2	0	11			
W	1	2	1	2	3	5	0	15		
Y	2	1	3	3	3	7	1	6	11	
V	4	1	7	5	5	3	2	1	3	8

Score: 4

5 - HYDROPHILIC

	N	C	Q	H	S	T
N	10					
C	1	13				
Q	4	1	9			
H	5	1	4	12		
S	5	3	4	3	8	
T	4	3	3	2	5	9

Score: 5

6 - NEGATIVE

	D	E
D	10	
E	6	9

Score: 8

7 - POSITIVE

	R	K
R	9	
K	6	9

Score: 8

Figure S2: Scoring Matrices employed in the 8 Digit profile, as derived from the normalized BLOSUM62 matrix, where the score is the rounded mean score $\bar{x}$. Each matrix has been labeled with both its 8DP's binding site value and the main phisico-chemical of its components. The amino acids in each matrix are represented with their 1-letter code.

5 - ALIPHATIC

	A	G	I	L	M	P	V
A	8						
G	4	10					
I	3	0	8				
L	3	0	6	8			
M	3	1	5	6	9		
P	3	2	1	1	2	11	
V	4	1	7	5	5	2	8

Score: 4.5

6 - AROMATIC

	F	W	Y
F	10		
W	5	15	
Y	7	6	11

Score: 9

7 - HYDROPHILIC

	N	C	Q	H	S	T
N	10					
C	1	13				
Q	4	1	9			
H	5	1	4	12		
S	5	3	4	3	8	
T	4	3	3	2	5	9

Score: 5

8 - NEGATIVE

	D	E
D	10	
E	6	9

Score: 8

9 - POSITIVE

	R	K
R	9	
K	6	9

Score: 8

Figure S3: Scoring Matrices for the 10 Digit profile derived from the BLOSUM62 matrix, where the score is the rounded mean score $\bar{x}$. Each matrix has been labeled with both its 10DP's binding site value and the main phisico-chemical of its components. The amino acids in each matrix are represented with their 1-letter code.

The score for each value of the profile was then calculated by getting the mean score, $\bar{x}$

$$\overline{x} = \frac{1}{n}(\sum_{i=1}^{n} x_i)$$

Where n is the total number of values in the matrix, and x_i is the normalised BLOSUM62 value assigned to each amino acid substitution.

GPCRtm

GPCRtm is an amino acid substitution matrix calculated for the membrane-spanning segments of GPCRs. It is specifically based on the rhodopsin family (Class A GPCRs).[3] By aligning more than one thousand membrane-spanning sequences of class A GPCRs from different organisms, the GPCRtm matrix was generated (Figure S4). Higher values indicate greater interchangeability of residues and therefore the GPCRtm matrix reveals different residue replacement frequencies compared to standard substitution matrices, such as BLOSUM62, where a larger score value for the positively (Lys, Arg, and His) and negatively (Asp, Glu) charged residues have a greater degree of interchangeable with one another.[3] The reason that an additional scoring matrix was integrated into the CPG tool is due to the fact that GPCRs should not be treated in the same way as other proteins. While there is a great degree of variability between their primary sequences, many instances have

GPCRtm

	A	C	D	E	F	G	H	I	K	L	M	N	P	Q	R	S	T	V	W	Y
**A**	2																			
**C**	0	5																		
**D**	-3	-3	9																	
**E**	-1	-3	5	6																
**F**	-1	0	-2	-2	2															
**G**	1	0	-1	1	0	4														
**H**	-1	-1	1	1	-1	-1	5													
**I**	-1	-1	-4	-2	0	-1	-1	2												
**K**	-1	-2	0	3	-2	-1	2	-2	6											
**L**	-1	-1	-4	-2	0	-1	-1	1	-2	2										
**M**	-1	0	-3	-1	0	0	0	1	-1	1	3									
**N**	-2	-3	2	0	-2	-2	2	-3	0	-3	-2	8								
**P**	-1	-3	0	0	-3	-2	0	-3	-3	-3	-2	-2	8							
**Q**	-1	-2	1	3	-1	-1	3	-2	3	-1	-2	1	0	5						
**R**	-1	-1	-1	2	-2	-1	2	-3	4	-2	-2	0	-2	3	7					
**S**	1	0	-2	1	-1	1	0	-1	0	-1	-1	0	0	0	-1	2				
**T**	1	0	-2	-1	-1	1	0	0	-1	0	0	-1	-1	-1	-1	1	2			
**V**	0	0	-3	-2	0	-1	-1	1	-2	0	0	-3	-3	-1	-2	-1	0	1		
**W**	-2	-1	-3	-1	0	-1	0	-2	-1	-1	-1	-2	-4	1	0	-2	-2	-1	8	
**Y**	-2	-1	-2	-1	1	-1	1	-2	-1	-1	-1	-1	-4	0	-2	-2	-2	-1	2	6

Figure S4: The GPCRtm matrix as extrapolated from [3]. All amino acids have been represented with their 1-letter code.

underlined that there are robust conservations in certain regions of GPCR protein sequences. By exploiting more conserved residues and giving scores according to the interchangeability of GPCRs

in particular, the goal was to achieve a more accurate scoring scheme. To integrate the GPCTtm matrix inside the 8DP and 10DP profiling schemes, the same procedure employed for the BLOSUM62 matrix was followed. In the 8DP case, the matching scores were calculated to be to 4, 5, 10, and 9 for the hydrophobic, hydrophilic, negative, and positive residues, respectively. For the case of 10DP, the GPCRtm procedure for the hydrophobic residues divided into aliphatic and aromatic residues, resulting in the scores of 4 and 5, respectively (refer to Fig S5 & S6).

4 - HYDROPHOBIC

	A	G	I	L	M	F	P	W	Y	V
A	6									
G	5	8								
I	3	3	6							
L	3	4	4	6						
M	3	4	4	5	7					
F	3	5	4	4	4	6				
P	3	2	1	1	1	1	12			
W	2	3	2	3	3	4	0	12		
Y	2	3	2	3	3	5	0	6	10	
V	4	3	5	4	4	4	1	3	3	5

Score: 4

5 - HYDROPHILIC

	N	C	Q	H	S	T
N	12					
C	1	9				
Q	5	2	5			
H	6	3	5	9		
S	4	2	5	4	6	
T	3	2	5	4	5	6

Score: 5

6 - NEGATIVE

	D	E
D	13	
E	9	10

Score: 10

7 - POSITIVE

	R	K
R	11	
K	8	10

Score: 9

Figure S5: Scoring Matrices for the 8 Digit profile derived from the GPCRtm matrix, where the score is the rounded mean score $\overline{x}$. Each matrix is labelled with its binding residues' profile value and its representative physico-chemical property. The amino acids in each matrix are represented with their 1-letter code.

5 - ALIPHATIC

	A	G	I	L	M	P	V
A	6						
G	5	8					
I	3	3	6				
L	3	4	4	6			
M	3	4	4	5	7		
P	3	2	1	1	1	12	
V	4	3	5	4	4	1	5

Score: 4

6 - AROMATIC

	F	W	Y
F	6		
W	4	12	
Y	4	3	3

Score: 5

7 - HYDROPHILIC

	N	C	Q	H	S	T
N	12					
C	1	9				
Q	5	2	5			
H	6	3	5	9		
S	4	2	5	4	6	
T	3	2	5	4	5	6

Score: 5

8 - NEGATIVE

	D	E
D	13	
E	9	10

Score: 10

9 - POSITIVE

	R	K
R	11	
K	8	10

Score: 9

Figure S6: Scoring Matrices for the 10 Digit profile derived from the GPCRtm matrix, where the score is the rounded mean score $\overline{x}$. Each matrix is labelled with its binding residues' profile value and its representative *physico-chemical property. The amino acids in each matrix are represented with their 1-letter code.*

ALIGNMENT MATRICES

To assess the potency of our profiling methods, we tested them on a pool of 55 GPCR pdb files, which we aligned one against the other. Through the generation of alignment tables and the measurement of normalized alignment scores, it was possible to evaluate the goodness of each pairwise comparison. The normalized score is expressed as

$$N=T/R$$

where T = Target protein score (the alignment score of the target protein against the reference protein) and R = Reference protein score (the alignment score value of the reference protein with itself). Self-alignment will give as outcome a value of N=1. We generated 4 alignment tables (composed by 55 × 55 elements), one for each combination of profiling scheme (8DP and 10DP) and substitution matrix

(Blosum62 and GPCRtm). For the sake of clarity, pdb files presenting the same GPCR have been clustered togheter.

The alignment tables were coloured with a “RdYlGn” colour palette, ranging from red (the lowest score present in the table) to dark green (the highest possible score). Because the normalized alignment scores have been grouped according to which protein they are present, it is possible to identify that alignments between the same GPCR have greater scores than alignments between different GPCRs, which is something that was expected (see Fig. S7). Conversely, results of great interest are the portions of the tables with high scores, born from the intersection of unreleated GPCRs. To point out the most promising alignments, we coloured in azure the squares containing a CPG value higher or equal to 0.5 (see Fig. S8-S11). This value indicates that there is, at least, a 50% similarity in profile alignment between the target and reference protein.

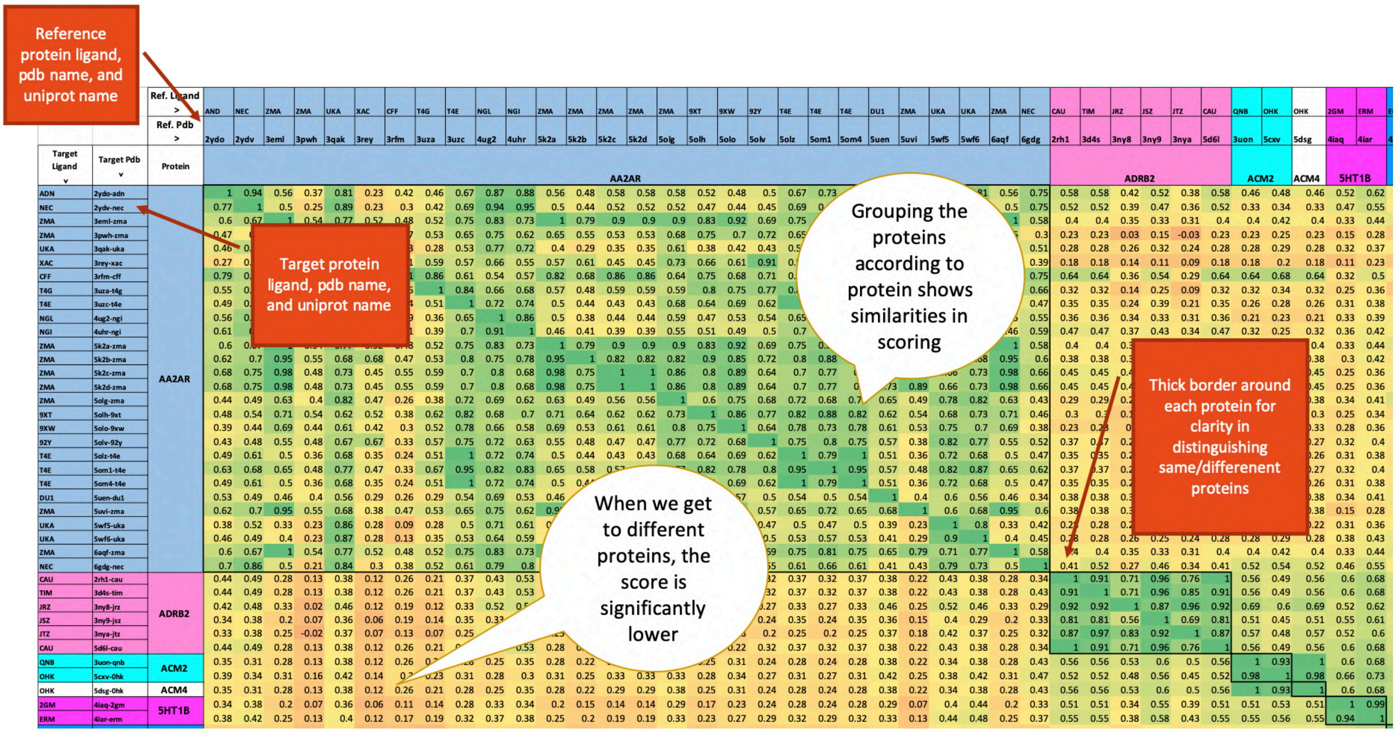

Figure S7: Annotated section of a generated alignment table. The cells representing the normalized alignment scores between the target and reference proteins were coloured in tones ranging from dark green (the highest possible score - which is 1) and red (the lowest score present in the table).

		Ref. Ligand >	ADN	NEC	ZMA	ZMA	UKA	XAC	CFF	T4G	T4E	NGL	NGI	ZMA	ZMA	ZMA	ZMA	ZMA	9XT	9XW	92Y	T4E	T4E	T4E	DU1	ZMA	UKA	UKA	ZMA	NEC	CAU	TIM	JRZ	JSZ	JTZ	CAU	QNB	OHK	OHK	2GM	ERM	ERM	ERM	ERM	7LD	H8G	H8D	H8J	H8M	AZJ	6AD	1Q5	73R	8ES	8EM	AQD	RET
		Ref. Pdb >	2ydo	2ydv	3eml	3pwh	3qak	3rey	3rfm	3uza	3uzc	4ug2	4uhr	5k2a	5k2b	5k2c	5k2d	5olg	5olh	5olo	5olv	5olz	5om1	5om4	5uen	5uvi	5wf5	5wf6	6aqf	6gdg	2rh1	3d4s	3ny8	3ny9	3nya	5d6l	3uon	5cxv	5dsg	4iaq	4iar	4ib4	4nc3	5tud	5tvn	6drx	6dry	6drz	6ds0	4ntj	4pxz	4z9g	5t1a	5unf	5unh	5wiv	6fuf
Target Ligand v	Target Pdb v	Protein	AA2AR																												ADRB2						ACM2		ACM4	5HT1B		5HT2B								P2Y12		CRF1	CCR2	AGTR2		DRD4	GNAO
ADN	2ydo-adn	AA2AR	1	0.73	0.53	0.37	0.38	0.11	0.59	0.38	0.4	0.5	0.55	0.53	0.54	0.57	0.57	0.39	0.4	0.29	0.33	0.4	0.57	0.4	0.42	0.49	0.29	0.38	0.53	0.61	0.32	0.32	0.34	0.23	0.24	0.32	0.24	0.26	0.24	0.22	0.26	0.07	0.11	0.3	0.28	0.29	0.01	0.24	0.15	-0.03	-0.19	0	-0.38	0.1	0.01	0.03	-0.08
NEC	2ydv-nec		0.9	1	0.58	0.24	0.57	0.17	0.38	0.45	0.55	0.72	0.78	0.58	0.61	0.63	0.63	0.45	0.45	0.36	0.38	0.55	0.61	0.55	0.38	0.55	0.46	0.4	0.58	0.79	0.35	0.36	0.33	0.26	0.23	0.35	0.18	0.23	0.18	0.28	0.32	0.13	0.17	0.28	0.33	0.34	0.08	0.21	0.2	0.04	-0.15	0.02	-0.34	0.15	0.03	0.06	0
ZMA	3eml-zma		0.48	0.43	1	0.54	0.31	0.43	0.66	0.47	0.42	0.42	0.37	1	0.91	0.96	0.96	0.6	0.68	0.65	0.44	0.42	0.6	0.42	0.35	0.91	0.23	0.31	1	0.39	0.1	0.1	0.2	0.04	0.11	0.1	0.13	0.14	0.13	0.04	0.09	0.08	0.13	0.33	0.09	0.09	0.12	0.23	0.15	-0.13	-0.25	-0.1	-0.3	-0.07	-0.08	-0.05	-0.26
ZMA	3pwh-zma		0.28	0.15	0.45	1	0.1	0.51	0.52	0.4	0.29	0.28	0.22	0.45	0.46	0.37	0.37	0.31	0.5	0.38	0.38	0.29	0.43	0.29	0.3	0.45	0.12	0.12	0.45	0.05	-0.06	-0.06	-0.15	-0.1	-0.19	-0.06	-0.04	-0.04	-0.04	-0.11	-0.05	-0.11	-0.08	0.04	-0.06	-0.07	-0.05	0.07	0.34	-0.26	-0.29	-0.36	-0.38	-0.07	-0.08	-0.09	-0.37
UKA	3qak-uka		0.67	0.81	0.6	0.24	1	0.3	-0.1	0.32	0.57	0.84	0.8	0.6	0.43	0.53	0.53	0.76	0.45	0.49	0.48	0.57	0.64	0.57	0.41	0.45	0.84	0.85	0.6	0.71	0.17	0.21	0.2	0.2	0.14	0.17	0.21	0.22	0.21	0.24	0.28	0.21	0.25	0.4	0.25	0.37	0.29	0.31	0.31	-0.14	-0.23	-0.07	-0.3	0.08	0.03	0.13	-0.37
XAC	3rey-xac		0.08	0.11	0.36	0.52	0.13	1	0.45	0.55	0.25	0.25	0.18	0.36	0.52	0.27	0.27	0.35	0.42	0.31	0.62	0.25	0.39	0.25	0.12	0.17	0.14	0.15	0.36	0.19	-0.09	-0.09	-0.11	-0.14	-0.16	-0.09	-0.09	-0.07	-0.09	-0.11	-0.05	-0.11	-0.08	0.04	-0.09	-0.09	-0.08	0.04	0.31	-0.21	-0.27	-0.4	-0.28	-0.09	-0.04	-0.11	-0.26
CFF	3rfm-cff		0.28	0.15	0.35	0.33	-0.03	0.28	1	0.45	0.09	0.03	0.06	0.35	0.37	0.41	0.41	0.12	0.24	0.15	0.21	0.09	0.19	0.09	0.11	0.32	-0.08	-0.03	0.35	0.24	0.12	0.12	0.05	0.03	-0.01	0.12	0.12	0.15	0.12	-0.04	0.01	-0.06	-0.03	0.11	0.11	0.12	0.03	0.15	0.18	-0.1	-0.19	-0.16	-0.28	-0.02	-0.13	-0.12	0
T4G	3uza-t4g		0.48	0.28	0.4	0.41	0.14	0.55	0.72	1	0.41	0.25	0.26	0.4	0.43	0.43	0.43	0.27	0.55	0.42	0.51	0.41	0.58	0.41	0.14	0.34	0.14	0.22	0.4	0.4	0.03	0.03	-0.07	-0.04	-0.11	0.03	0.03	0.05	0.03	-0.01	0.04	-0.09	-0.06	0.06	0.01	0.01	-0.05	0.07	0.24	-0.18	-0.25	-0.26	-0.23	0.07	-0.02	-0.04	-0.21
T4E	3uzc-t4e		0.53	0.59	0.62	0.5	0.44	0.43	0.24	0.7	1	0.59	0.62	0.62	0.65	0.55	0.55	0.67	0.74	0.69	0.7	1	0.91	1	0.41	0.47	0.42	0.42	0.62	0.44	0.17	0.17	0.08	0.18	0.01	0.17	0.13	0.11	0.13	0.13	0.17	0.05	0.09	0.16	0.15	0.26	0.09	0.23	0.48	-0.12	-0.25	-0.03	-0.45	0.19	0.13	0.13	-0.34
NGL	4ug2-ngi		0.77	0.89	0.71	0.57	0.73	0.49	0.1	0.49	0.68	1	0.87	0.71	0.57	0.65	0.65	0.65	0.58	0.6	0.62	0.68	0.76	0.68	0.6	0.57	0.68	0.58	0.71	0.66	0.24	0.26	0.3	0.17	0.2	0.24	0.08	0.12	0.08	0.22	0.26	0.19	0.24	0.36	0.23	0.24	0.17	0.19	0.41	-0.06	-0.21	-0.12	-0.2	0.17	0.12	0.19	-0.21
NGI	4uhr-ngi		0.8	0.92	0.58	0.41	0.67	0.34	0.17	0.49	0.68	0.83	1	0.58	0.61	0.51	0.51	0.56	0.58	0.47	0.51	0.68	0.76	0.68	0.4	0.43	0.54	0.51	0.58	0.69	0.38	0.4	0.38	0.29	0.27	0.38	0.22	0.15	0.22	0.26	0.3	0.14	0.19	0.28	0.28	0.29	0.11	0.24	0.31	0.08	-0.12	-0.05	-0.31	0.17	0.12	0.16	-0.16
ZMA	5k2a-zma		0.48	0.43	1	0.54	0.31	0.43	0.66	0.47	0.42	0.42	0.37	1	0.91	0.96	0.96	0.6	0.68	0.65	0.44	0.42	0.6	0.42	0.35	0.91	0.23	0.31	1	0.39	0.1	0.1	0.2	0.04	0.11	0.1	0.13	0.14	0.13	0.04	0.09	0.08	0.13	0.33	0.09	0.09	0.12	0.23	0.15	-0.13	-0.25	-0.1	-0.3	-0.07	-0.08	-0.05	-0.26
ZMA	5k2b-zma		0.42	0.38	0.76	0.46	0.19	0.51	0.59	0.43	0.38	0.28	0.32	0.76	1	0.71	0.71	0.43	0.61	0.47	0.41	0.38	0.54	0.38	0.19	0.64	0.12	0.19	0.76	0.35	0.06	0.06	0.08	-0.01	0.01	0.06	0.06	0.09	0.06	0.02	0.07	-0.01	0.03	0.19	0.06	0.07	-0.03	0.2	0.13	-0.1	-0.24	-0.17	-0.38	-0.09	-0.04	-0.15	-0.16
ZMA	5k2c-zma		0.48	0.43	0.89	0.41	0.26	0.3	0.72	0.47	0.35	0.36	0.3	0.89	0.78	1	1	0.52	0.58	0.57	0.35	0.35	0.51	0.35	0.38	0.96	0.18	0.26	0.89	0.39	0.13	0.13	0.23	0.07	0.14	0.13	0.15	0.16	0.15	-0.02	0.03	0.03	0.07	0.25	0.11	0.12	0.04	0.15	0.07	-0.1	-0.24	-0.07	-0.27	-0.05	-0.15	-0.04	-0.21
ZMA	5k2d-zma		0.48	0.43	0.89	0.41	0.26	0.3	0.72	0.47	0.35	0.36	0.3	0.89	0.78	1	1	0.52	0.58	0.57	0.35	0.35	0.51	0.35	0.38	0.96	0.18	0.26	0.89	0.39	0.13	0.13	0.23	0.07	0.14	0.13	0.15	0.16	0.15	-0.02	0.03	0.03	0.07	0.25	0.11	0.12	0.04	0.15	0.07	-0.1	-0.24	-0.07	-0.27	-0.05	-0.15	-0.04	-0.21
ZMA	5olg-zma		0.48	0.46	0.82	0.5	0.54	0.55	0.31	0.43	0.62	0.53	0.48	0.82	0.7	0.76	0.76	1	0.65	0.74	0.67	0.62	0.72	0.62	0.53	0.7	0.43	0.52	0.82	0.39	0.15	0.13	0.16	0.11	0.09	0.15	0.21	0.14	0.21	0.13	0.17	0.22	0.27	0.3	0.19	0.28	0.22	0.33	0.38	-0.13	-0.27	-0.03	-0.33	0.02	-0.02	0.05	-0.47
9XT	5olh-9xt		0.42	0.38	0.76	0.67	0.27	0.55	0.52	0.72	0.57	0.39	0.41	0.76	0.83	0.71	0.71	0.53	1	0.69	0.63	0.57	0.78	0.57	0.41	0.64	0.25	0.34	0.76	0.32	0.04	0.04	-0.02	-0.01	-0.07	0.04	0.06	0.07	0.06	0	0.05	-0.03	0.01	0.16	0.04	0.04	-0.05	0.17	0.24	-0.18	-0.29	-0.17	-0.25	0.16	0.13	-0.03	-0.37
9XW	5olo-9xw		0.35	0.35	0.85	0.59	0.33	0.47	0.38	0.64	0.62	0.47	0.39	0.85	0.74	0.8	0.8	0.71	0.81	1	0.57	0.62	0.72	0.62	0.47	0.74	0.39	0.39	0.85	0.26	0.03	0.03	0.07	0.04	0	0.03	0.13	0.14	0.13	0.04	0.09	0.08	0.13	0.23	0.09	0.09	0.12	0.23	0.3	-0.18	-0.32	-0.09	-0.3	0.05	0	0.04	-0.47
92Y	5olv-92y		0.35	0.32	0.51	0.52	0.29	0.83	0.45	0.68	0.55	0.42	0.37	0.51	0.57	0.43	0.43	0.56	0.65	0.5	1	0.55	0.72	0.55	0.33	0.34	0.35	0.36	0.51	0.45	0.12	0.12	0.05	0.03	-0.01	0.12	0.04	0.07	0.04	0.06	0.11	0.03	0.06	0.24	0.11	0.12	-0.05	0.17	0.42	-0.13	-0.25	-0.1	-0.41	0.16	0.13	0.11	-0.37
T4E	5olz-t4e		0.53	0.59	0.62	0.5	0.44	0.43	0.24	0.7	1	0.59	0.62	0.62	0.65	0.55	0.55	0.67	0.74	0.69	0.7	1	0.91	1	0.41	0.47	0.42	0.42	0.62	0.44	0.17	0.17	0.08	0.18	0.01	0.17	0.13	0.11	0.13	0.13	0.17	0.05	0.09	0.16	0.15	0.26	0.09	0.23	0.48	-0.12	-0.25	-0.03	-0.45	0.19	0.13	0.13	-0.34
T4E	5om1-t4e		0.63	0.55	0.73	0.63	0.41	0.55	0.45	0.83	0.76	0.55	0.59	0.73	0.78	0.67	0.67	0.64	0.84	0.67	0.76	0.76	1	0.76	0.39	0.6	0.38	0.49	0.73	0.53	0.13	0.13	0.1	0.07	0.03	0.13	0.08	0.08	0.08	0.1	0.14	0.03	0.06	0.24	0.11	0.12	-0.05	0.17	0.32	-0.1	-0.24	-0.07	-0.36	0.14	0.11	0.04	-0.37
T4E	5om4-t4e		0.53	0.59	0.62	0.5	0.44	0.43	0.24	0.7	1	0.59	0.62	0.62	0.65	0.55	0.55	0.67	0.74	0.69	0.7	1	0.91	1	0.41	0.47	0.42	0.42	0.62	0.44	0.17	0.17	0.08	0.18	0.01	0.17	0.13	0.11	0.13	0.13	0.17	0.05	0.09	0.16	0.15	0.26	0.09	0.23	0.48	-0.12	-0.25	-0.03	-0.45	0.19	0.13	0.13	-0.34
DU1	5uen-du1		0.57	0.41	0.51	0.52	0.31	0.21	0.31	0.23	0.41	0.52	0.37	0.51	0.33	0.59	0.59	0.56	0.53	0.53	0.41	0.41	0.46	0.41	1	0.51	0.31	0.31	0.51	0.19	0.22	0.22	0.31	0.21	0.21	0.22	0.22	0.23	0.22	0.1	0.14	0.13	0.17	0.29	0.28	0.29	0.11	0.21	0.37	-0.1	-0.25	0	-0.16	0.24	0.09	0.18	-0.29
ZMA	5uvi-zma		0.38	0.35	0.78	0.46	0.2	0.17	0.52	0.34	0.28	0.29	0.23	0.78	0.65	0.88	0.88	0.44	0.48	0.49	0.25	0.28	0.42	0.28	0.3	1	0.13	0.2	0.78	0.29	0.05	0.05	0.13	0	0.06	0.05	0.08	0.08	0.08	-0.09	-0.03	-0.02	0.02	0.17	0.04	0.04	-0.04	0.07	-0.01	-0.18	-0.29	-0.17	-0.36	-0.12	-0.21	-0.09	-0.37
UKA	5wf5-uka		0.57	0.73	0.49	0.3	0.94	0.36	-0.31	0.36	0.62	0.87	0.74	0.49	0.3	0.41	0.41	0.68	0.48	0.64	0.65	0.62	0.67	0.62	0.45	0.32	1	0.87	0.49	0.61	0.19	0.23	0.23	0.22	0.17	0.19	0.13	0.14	0.13	0.24	0.3	0.21	0.25	0.4	0.28	0.29	0.21	0.23	0.35	-0.22	-0.28	-0.07	-0.39	0.13	0.07	0.3	-0.53
UKA	5wf6-uka		0.67	0.61	0.6	0.28	0.84	0.34	-0.1	0.49	0.56	0.7	0.63	0.6	0.43	0.53	0.53	0.73	0.58	0.57	0.6	0.56	0.76	0.56	0.41	0.45	0.76	1	0.6	0.58	0.21	0.21	0.23	0.09	0.14	0.21	0.19	0.22	0.19	0.32	0.36	0.28	0.32	0.4	0.35	0.39	0.32	0.28	0.21	-0.17	-0.28	-0.12	-0.39	0.1	0.05	0.22	-0.37
ZMA	6aqf-zma		0.48	0.43	1	0.54	0.31	0.43	0.66	0.47	0.42	0.42	0.37	1	0.91	0.96	0.96	0.6	0.68	0.65	0.44	0.42	0.6	0.42	0.35	0.91	0.23	0.31	1	0.39	0.1	0.1	0.2	0.04	0.11	0.1	0.13	0.14	0.13	0.04	0.09	0.08	0.13	0.33	0.09	0.09	0.12	0.23	0.15	-0.13	-0.25	-0.1	-0.3	-0.07	-0.08	-0.05	-0.26
NEC	6gdg-nec		0.62	0.66	0.44	0.07	0.42	0.26	0.52	0.53	0.34	0.45	0.49	0.44	0.48	0.47	0.47	0.32	0.32	0.22	0.44	0.34	0.49	0.34	0.14	0.38	0.32	0.34	0.44	1	0.21	0.32	0.13	0.21	0.21	0.21	0.31	0.35	0.31	0.23	0.27	0.08	0.12	0.31	0.26	0.28	0.11	0.33	0.15	-0.1	-0.14	-0.07	-0.28	0.08	-0.01	0.02	-0.08
CAU	2rh1-cau	ADRB2	0.37	0.34	0.15	-0.11	0.11	-0.15	0.31	0.02	0.15	0.2	0.32	0.15	0.11	0.2	0.2	0.16	0.05	0.03	0.14	0.15	0.13	0.15	0.21	0.09	0.12	0.13	0.15	0.19	1	0.88	0.87	0.78	0.81	1	0.5	0.45	0.5	0.44	0.47	0.27	0.25	0.34	0.59	0.49	0.2	0.37	0.34	0.27	-0.05	0.19	-0.44	0.03	-0.09	0.42	-0.11
TIM	3d4s-tim		0.37	0.34	0.15	-0.11	0.11	-0.15	0.31	0.02	0.15	0.2	0.32	0.15	0.11	0.2	0.2	0.13	0.05	0.03	0.14	0.15	0.13	0.15	0.21	0.09	0.12	0.13	0.15	0.34	0.88	1	0.87	0.78	0.94	0.88	0.47	0.42	0.47	0.46	0.49	0.25	0.24	0.34	0.61	0.51	0.2	0.35	0.34	0.16	-0.03	0.16	-0.31	0.13	-0.01	0.36	0.21
JRZ	3ny8-jrz		0.3	0.23	0.22	-0.2	0.11	-0.15	0.1	-0.11	0.05	0.2	0.23	0.22	0.11	0.27	0.27	0.13	-0.02	0.06	0.05	0.05	0.07	0.05	0.24	0.17	0.12	0.13	0.22	0.1	0.68	0.68	1	0.51	0.81	0.68	0.47	0.42	0.47	0.23	0.27	0.19	0.16	0.26	0.31	0.22	0.2	0.21	0.08	0.13	-0.06	0.16	-0.44	-0.03	-0.09	0.24	0.11
JSZ	3ny9-jsz		0.3	0.28	0.07	-0.2	0.13	-0.28	0.1	-0.11	0.2	0.15	0.28	0.07	-0.02	0.12	0.12	0.13	-0.02	0.06	0.05	0.2	0.07	0.2	0.24	0	0.14	0.1	0.07	0.24	0.92	0.92	0.77	1	0.86	0.92	0.51	0.46	0.51	0.49	0.52	0.3	0.28	0.26	0.54	0.54	0.22	0.37	0.37	0.19	0.01	0.29	-0.41	0.06	-0.07	0.47	0.05
JTZ	3nya-jtz		0.23	0.18	0.15	-0.28	0.08	-0.23	-0.03	-0.19	0	0.15	0.18	0.15	0.02	0.2	0.2	0.08	-0.08	0	-0.02	0	0.01	0	0.19	0.09	0.08	0.1	0.15	0.18	0.73	0.85	0.93	0.65	1	0.73	0.42	0.36	0.42	0.33	0.37	0.22	0.2	0.31	0.44	0.36	0.14	0.16	0.14	0.18	0	0.22	-0.38	0.01	-0.05	0.33	0.32
CAU	5d6l-cau		0.37	0.34	0.15	-0.11	0.11	-0.15	0.31	0.02	0.15	0.2	0.32	0.15	0.11	0.2	0.2	0.16	0.05	0.03	0.14	0.15	0.13	0.15	0.21	0.09	0.12	0.13	0.15	0.19	1	0.88	0.87	0.78	0.81	1	0.5	0.45	0.5	0.44	0.47	0.27	0.25	0.34	0.59	0.49	0.2	0.37	0.34	0.27	-0.05	0.19	-0.44	0.03	-0.09	0.42	-0.11
QNB	3uon-qnb	ACM2	0.27	0.19	0.18	-0.07	0.13	-0.15	0.31	0.02	0.11	0.08	0.16	0.18	0.11	0.24	0.24	0.21	0.08	0.14	0.05	0.11	0.07	0.11	0.21	0.13	0.07	0.12	0.18	0.34	0.5	0.47	0.61	0.43	0.47	0.5	1	0.97	1	0.43	0.46	0.33	0.39	0.35	0.47	0.57	0.38	0.63	0.24	0.1	-0.01	0.02	-0.38	-0.01	-0.14	0.24	-0.05
OHK	5cxv-0hk		0.27	0.19	0.18	-0.07	0.13	-0.11	0.38	0.06	0.09	0.08	0.09	0.18	0.15	0.24	0.24	0.13	0.08	0.14	0.08	0.09	0.07	0.09	0.21	0.13	0.07	0.13	0.18	0.37	0.4	0.4	0.51	0.37	0.39	0.4	0.92	1	0.92	0.45	0.48	0.34	0.4	0.38	0.49	0.49	0.3	0.55	0.24	0.03	-0.01	-0.09	-0.34	0.01	-0.12	0.26	-0.18
OHK	5dsg-0hk	ACM4	0.27	0.19	0.18	-0.07	0.13	-0.15	0.31	0.02	0.11	0.08	0.16	0.18	0.11	0.24	0.24	0.21	0.08	0.14	0.05	0.11	0.07	0.11	0.21	0.13	0.07	0.12	0.18	0.34	0.5	0.47	0.61	0.43	0.47	0.5	1	0.97	1	0.43	0.46	0.33	0.39	0.35	0.47	0.57	0.38	0.63	0.24	0.1	-0.01	0.02	-0.38	-0.01	-0.14	0.24	-0.05
2GM	4iaq-2gm	5HT1B	0.33	0.36	0.07	-0.22	0.17	-0.21	-0.14	-0.02	0.1	0.17	0.23	0.07	0.04	-0.02	-0.02	0.16	0	0.06	0.1	0.1	0.09	0.1	0.12	-0.15	0.15	0.24	0.07	0.34	0.53	0.55	0.41	0.5	0.44	0.53	0.51	0.57	0.51	1	0.93	0.49	0.49	0.6	0.84	0.62	0.46	0.48	0.28	-0.01	-0.16	0.26	-0.31	-0.01	-0.05	0.4	-0.21
ERM	4iar-erm		0.42	0.43	0.16	-0.11	0.22	-0.11	0.03	0.09	0.16	0.23	0.29	0.16	0.15	0.08	0.08	0.23	0.08	0.1	0.17	0.16	0.16	0.16	0.19	-0.04	0.19	0.29	0.16	0.42	0.59	0.62	0.49	0.55	0.51	0.59	0.58	0.64	0.58	0.98	1	0.55	0.55	0.59	0.9	0.68	0.43	0.55	0.35	-0.04	-0.12	0.22	-0.34	-0.03	-0.07	0.44	-0.26
ERM	4ib4-erm	5HT2B	0.13	0.2	0.18	-0.28	0.27	-0.28	-0.24	-0.23	0.09	0.24	0.2	0.18	-0.02	0.08	0.08	0.35	-0.05	0.14	0.05	0.09	0.04	0.09	0.19	-0.04	0.24	0.31	0.18	0.15	0.41	0.38	0.36	0.37	0.37	0.41	0.5	0.54	0.5	0.62	0.66	1	0.96	0.8	0.7	0.57	0.64	0.76	0.38	-0.19	-0.2	0.26	-0.47	-0.01	-0.05	0.35	-0.47
ERM	4nc3-erm		0.2	0.26	0.25	-0.2	0.29	-0.19	-0.1	-0.15	0.14	0.28	0.24	0.25	0.07	0.16	0.16	0.4	0.02	0.17	0.11	0.14	0.1	0.14	0.24	0.04	0.25	0.33	0.25	0.21	0.35	0.32	0.28	0.32	0.3	0.35	0.55	0.59	0.55	0.58	0.62	0.9	1	0.75	0.64	0.62	0.7	0.81	0.44	-0.14	-0.16	0.05	-0.41	0.03	-0.01	0.3	-0.37
ERM	5tud-erm		0.4	0.3	0.47	0.07	0.3	0.06	0.31	0.11	0.16	0.32	0.25	0.47	0.33	0.39	0.39	0.32	0.21	0.25	0.3	0.16	0.28	0.16	0.29	0.3	0.27	0.3	0.47	0.4	0.36	0.36	0.36	0.24	0.37	0.36	0.36	0.41	0.36	0.52	0.48	0.54	0.55	1	0.6	0.46	0.54	0.65	0.46	-0.13	-0.25	0.1	-0.22	0.03	0.01	0.2	-0.24
7LD	5tvn-7ld		0.37	0.36	0.13	-0.11	0.2	-0.15	0.31	0.02	0.14	0.21	0.26	0.13	0.11	0.18	0.18	0.2	0.05	0.1	0.14	0.14	0.13	0.14	0.28	0.06	0.19	0.27	0.13	0.34	0.6	0.63	0.48	0.45	0.5	0.6	0.49	0.53	0.49	0.72	0.73	0.47	0.47	0.59	1	0.79	0.54	0.65	0.46	0.06	-0.13	0.26	-0.36	0.06	-0.07	0.35	-0.05
H8G	6drx-h8g		0.37	0.36	0.13	-0.11	0.27	-0.15	0.31	0.02	0.25	0.21	0.26	0.13	0.11	0.18	0.18	0.28	0.05	0.1	0.14	0.25	0.13	0.25	0.28	0.06	0.19	0.29	0.13	0.34	0.47	0.5	0.34	0.45	0.39	0.47	0.55	0.5	0.55	0.51	0.53	0.36	0.43	0.44	0.75	1	0.59	0.71	0.42	0.06	-0.13	0.12	-0.55	0.06	-0.07	0.28	-0.05
H8D	6dry-h8d		0.02	0.08	0.16	-0.09	0.21	-0.13	0.07	-0.09	0.09	0.14	0.09	0.16	-0.04	0.06	0.06	0.23	-0.06	0.12	-0.06	0.09	-0.06	0.09	0.1	-0.06	0.14	0.23	0.16	0.13	0.19	0.19	0.25	0.18	0.16	0.19	0.37	0.31	0.37	0.38	0.34	0.42	0.48	0.51	0.51	0.59	1	0.77	0.49	-0.19	-0.24	-0.26	-0.19	-0.1	-0.14	0.09	0
H8J	6drz-h8j		0.3	0.22	0.31	0.11	0.22	0.06	0.38	0.11	0.21	0.15	0.21	0.31	0.33	0.22	0.22	0.33	0.21	0.24	0.21	0.21	0.19	0.21	0.2	0.11	0.14	0.2	0.31	0.4	0.36	0.33	0.26	0.3	0.17	0.36	0.6	0.55	0.6	0.39	0.42	0.48	0.55	0.61	0.61	0.7	0.76	1	0.52	-0.16	-0.22	-0.1	-0.33	-0.01	-0.05	0.12	-0.18
H8M	6ds0-h8m		0.18	0.19	0.2	0.52	0.21	0.47	0.45	0.36	0.42	0.32	0.25	0.2	0.2	0.1	0.1	0.36	0.27	0.29	0.48	0.42	0.34	0.42	0.33	-0.02	0.21	0.17	0.2	0.18	0.31	0.31	0.1	0.28	0.14	0.31	0.22	0.23	0.22	0.23	0.27	0.22	0.27	0.41	0.42	0.39	0.46	0.49	1	-0.12	-0.2	-0.16	-0.22	0.02	-0.02	0.15	0.08
AZJ	4ntj-azj	P2Y12	-0.03	0.04	-0.18	-0.43	-0.1	-0.34	-0.28	-0.3	-0.11	-0.05	0.07	-0.18	-0.17	-0.16	-0.16	-0.13	-0.23	-0.19	-0.16	-0.11	-0.12	-0.11	-0.1	-0.3	-0.14	-0.12	-0.18	-0.13	0.27	0.15	0.16	0.16	0.2	0.27	0.1	0.03	0.1	-0.01	-0.03	-0.13	-0.1	-0.12	0.06	0.07	-0.2	-0.16	-0.13	1	0.34	0.16	-0.52	0.13	0.13	0.04	0.08
6AD	4pxz-6ad		-0.38	-0.24	-0.56	-0.76	-0.27	-0.7	-0.79	-0.66	-0.38	-0.28	-0.17	-0.56	-0.63	-0.57	-0.57	-0.44	-0.56	-0.54	-0.49	-0.38	-0.43	-0.38	-0.39	-0.74	-0.29	-0.32	-0.56	-0.27	-0.12	-0.05	-0.11	0.01	0	-0.12	-0.09	-0.01	-0.09	-0.22	-0.15	-0.25	-0.23	-0.39	-0.2	-0.21	-0.38	-0.36	-0.34	0.49	1	-0.24	-0.72	-0.2	-0.15	-0.02	-0.29
1Q5	4z9g-1q5	CRF1	0	0.05	-0.11	-0.43	-0.04	-0.47	-0.28	-0.3	-0.03	-0.04	-0.02	-0.11	-0.22	-0.08	-0.08	-0.03	-0.16	-0.07	-0.1	-0.03	-0.06	-0.03	0	-0.21	-0.03	-0.07	-0.11	-0.06	0.1	0.1	0.15	0.18	0.19	0.1	0.01	-0.07	0.01	0.16	0.13	0.13	0.03	0.07	0.19	0.08	-0.21	-0.08	-0.11	0.12	-0.07	1	-0.75	0	-0.13	0.01	-0.13
73R	5t1a-73r	CCR2	-0.5	-0.36	-0.35	-0.52	-0.23	-0.38	-0.62	-0.38	-0.4	-0.2	-0.29	-0.35	-0.52	-0.33	-0.33	-0.28	-0.26	-0.26	-0.41	-0.4	-0.39	-0.4	-0.14	-0.49	-0.25	-0.31	-0.35	-0.37	-0.37	-0.26	-0.44	-0.28	-0.34	-0.37	-0.32	-0.31	-0.32	-0.25	-0.26	-0.28	-0.26	-0.21	-0.33	-0.5	-0.17	-0.29	-0.2	-0.43	-0.38	-0.83	1	-0.09	-0.13	-0.38	-0.84
8ES	5unf-8es	AGTR2	0.15	0.18	-0.11	-0.13	0.07	-0.17	-0.07	0.13	0.2	0.16	0.17	-0.11	-0.17	-0.08	-0.08	0.03	0.23	0.06	0.22	0.2	0.18	0.2	0.26	-0.21	0.09	0.09	-0.11	0.11	0.05	0.14	-0.05	0.05	0.01	0.05	-0.01	0.01	-0.01	0	-0.02	-0.01	0.03	0.04	0.07	0.08	-0.12	-0.01	0.03	0.14	-0.14	0	-0.12	1	0.78	0.14	-0.34
8EM	5unh-8em		0.02	0.04	-0.15	-0.17	0.03	-0.09	-0.41	-0.04	0.15	0.12	0.13	-0.15	-0.09	-0.27	-0.27	-0.03	0.19	0	0.19	0.15	0.15	0.15	0.11	-0.43	0.06	0.05	-0.15	-0.02	-0.1	-0.01	-0.15	-0.08	-0.07	-0.1	-0.17	-0.15	-0.17	-0.04	-0.06	-0.04	-0.01	0.01	-0.07	-0.08	-0.17	-0.07	-0.03	0.16	-0.1	-0.21	-0.19	0.86	1	0.07	-0.45
AQD	5wiv-aqd	DRD4	0	0.07	-0.11	-0.22	0.14	-0.26	-0.45	-0.11	0.17	0.25	0.18	-0.11	-0.37	-0.08	-0.08	0.08	-0.05	0.06	0.19	0.17	0.04	0.17	0.25	-0.21	0.29	0.24	-0.11	-0.02	0.62	0.53	0.51	0.58	0.53	0.62	0.33	0.39	0.33	0.48	0.51	0.31	0.29	0.28	0.5	0.42	0.13	0.17	0.24	0.06	-0.02	0.02	-0.62	0.17	0.03	1	-0.42
RET	6fuf-ret	GNAO	-0.05	0	-0.18	-0.3	-0.13	-0.21	0	-0.17	-0.16	-0.09	-0.07	-0.18	-0.13	-0.16	-0.16	-0.24	-0.23	-0.25	-0.22	-0.16	-0.21	-0.16	-0.14	-0.3	-0.17	-0.13	-0.18	-0.05	-0.01	0.1	0.07	0.02	0.17	-0.01	-0.03	-0.11	-0.03	-0.05	-0.07	-0.14	-0.12	-0.1	0.01	0.01	0	-0.09	0.04	0.04	-0.08	-0.09	-0.45	-0.15	-0.18	-0.12	1

Blosum62 score with -2.0 miss - 8 digit profile

Figure S8: Matrix of the normalized scores for the 8 digit profiling scheme using BLOSUM62 Scoring with a miss and gap penalty of -2. Each element has been coloured following a RdYlGn palette, where higher scores are green in colour, while the lower scores are red. The elements coloured in azure are all values that are >0.5 as these are scores that are of interest.

Blosum62 score with -2.0 miss - 10 digit profile

Target Ligand v	Target Pdb v	Protein	ADN	NEC	ZMA	ZMA	UKA	XAC	CFF	T4G	T4E	NGL	NGI	ZMA	ZMA	ZMA	ZMA	ZMA	9XT	9XW	92Y	T4E	T4E	T4E	DU1	ZMA	UKA	UKA	ZMA	NEC
	Ref. Pdb >		2ydo	2ydv	3eml	3pwh	3qak	3rey	3rfm	3uza	3uzc	4ug2	4uhr	5k2a	5k2b	5k2c	5k2d	5olg	5olh	5olo	5olv	5olz	5om1	5om4	5uen	5uvi	5wf5	5wf6	6aqf	6gdg
		Protein	AA2AR																											
ADN	2ydo-adn	AA2AR	1	0.77	0.61	0.38	0.42	0.14	0.67	0.46	0.47	0.55	0.6	0.61	0.63	0.65	0.65	0.44	0.42	0.25	0.41	0.47	0.63	0.47	0.4	0.59	0.34	0.42	0.61	0.67
NEC	2ydv-nec	AA2AR	0.92	1	0.65	0.27	0.59	0.19	0.51	0.51	0.6	0.75	0.8	0.65	0.68	0.7	0.7	0.49	0.45	0.31	0.45	0.6	0.66	0.6	0.36	0.64	0.49	0.47	0.65	0.83
ZMA	3eml-zma	AA2AR	0.57	0.51	1	0.41	0.37	0.41	0.73	0.53	0.49	0.49	0.44	1	0.93	0.97	0.97	0.61	0.54	0.57	0.5	0.49	0.65	0.49	0.31	0.93	0.29	0.37	1	0.49
ZMA	3pwh-zma	AA2AR	0.3	0.18	0.35	1	0.19	0.57	0.6	0.44	0.24	0.24	0.18	0.35	0.33	0.41	0.41	0.37	0.51	0.32	0.44	0.24	0.35	0.24	0.37	0.47	0.19	0.2	0.35	0.11
UKA	3qak-uka	AA2AR	0.73	0.84	0.68	0.4	1	0.41	0.12	0.41	0.63	0.86	0.83	0.68	0.56	0.62	0.62	0.81	0.54	0.54	0.59	0.63	0.7	0.63	0.49	0.56	0.86	0.86	0.68	0.76
XAC	3rey-xac	AA2AR	0.1	0.12	0.34	0.54	0.18	1	0.42	0.52	0.23	0.23	0.17	0.34	0.35	0.26	0.26	0.36	0.4	0.31	0.58	0.23	0.34	0.23	0.19	0.17	0.18	0.2	0.34	0.19
CFF	3rfm-cff	AA2AR	0.34	0.21	0.39	0.38	0.04	0.28	1	0.51	0.15	0.1	0.12	0.39	0.41	0.45	0.45	0.17	0.26	0.23	0.25	0.15	0.25	0.15	0.08	0.37	-0.01	0.04	0.39	0.3
T4G	3uza-t4g	AA2AR	0.35	0.33	0.43	0.42	0.18	0.52	0.78	1	0.43	0.29	0.31	0.43	0.46	0.46	0.46	0.29	0.47	0.47	0.49	0.43	0.58	0.43	0.11	0.39	0.18	0.25	0.43	0.43
T4E	3uzc-t4e	AA2AR	0.62	0.65	0.69	0.38	0.48	0.39	0.4	0.75	1	0.64	0.67	0.69	0.73	0.64	0.64	0.66	0.6	0.63	0.68	1	0.93	1	0.38	0.58	0.46	0.48	0.69	0.53
NGL	4ug2-ngi	AA2AR	0.81	0.91	0.76	0.44	0.73	0.44	0.29	0.56	0.71	1	0.89	0.76	0.66	0.72	0.72	0.64	0.48	0.53	0.62	0.71	0.8	0.71	0.51	0.66	0.68	0.63	0.76	0.72
NGI	4uhr-ngi	AA2AR	0.84	0.93	0.66	0.32	0.68	0.32	0.34	0.56	0.71	0.85	1	0.66	0.69	0.61	0.61	0.57	0.48	0.42	0.53	0.71	0.8	0.71	0.38	0.54	0.56	0.57	0.66	0.75
ZMA	5k2a-zma	AA2AR	0.57	0.51	1	0.41	0.37	0.41	0.73	0.53	0.49	0.49	0.44	1	0.93	0.97	0.97	0.61	0.54	0.57	0.5	0.49	0.65	0.49	0.14	0.93	0.29	0.37	1	0.49
ZMA	5k2b-zma	AA2AR	0.51	0.46	0.8	0.33	0.26	0.37	0.66	0.49	0.45	0.36	0.4	0.8	1	0.76	0.76	0.47	0.49	0.41	0.47	0.45	0.6	0.45	0.18	0.7	0.19	0.26	0.8	0.45
ZMA	5k2c-zma	AA2AR	0.57	0.51	0.9	0.44	0.32	0.3	0.78	0.53	0.42	0.43	0.38	0.9	0.82	1	1	0.54	0.57	0.49	0.42	0.42	0.57	0.42	0.33	0.97	0.24	0.32	0.9	0.49
ZMA	5k2d-zma	AA2AR	0.57	0.51	0.9	0.44	0.32	0.3	0.78	0.53	0.42	0.43	0.38	0.9	0.82	1	1	0.54	0.57	0.49	0.42	0.42	0.57	0.42	0.33	0.97	0.24	0.32	0.9	0.49
ZMA	5olg-zma	AA2AR	0.58	0.54	0.85	0.61	0.61	0.62	0.45	0.5	0.65	0.57	0.53	0.85	0.76	0.81	0.81	1	0.68	0.66	0.74	0.65	0.74	0.65	0.58	0.76	0.51	0.6	0.85	0.49
9XT	5olh-9xt	AA2AR	0.49	0.44	0.68	0.74	0.37	0.61	0.62	0.73	0.54	0.38	0.4	0.68	0.72	0.76	0.76	0.61	1	0.52	0.71	0.54	0.71	0.54	0.56	0.71	0.35	0.44	0.68	0.41
9XW	5olo-9xw	AA2AR	0.28	0.29	0.69	0.45	0.36	0.46	0.51	0.69	0.54	0.41	0.34	0.69	0.57	0.64	0.64	0.57	0.5	1	0.45	0.54	0.6	0.54	0.29	0.58	0.4	0.41	0.69	0.21
92Y	5olv-92y	AA2AR	0.47	0.42	0.6	0.62	0.39	0.86	0.56	0.73	0.59	0.48	0.43	0.6	0.66	0.54	0.54	0.64	0.68	0.45	1	0.59	0.74	0.59	0.44	0.47	0.44	0.46	0.6	0.55
T4E	5olz-t4e	AA2AR	0.62	0.65	0.69	0.38	0.48	0.39	0.4	0.75	1	0.64	0.67	0.69	0.73	0.64	0.64	0.66	0.6	0.63	0.68	1	0.93	1	0.38	0.58	0.46	0.48	0.69	0.53
T4E	5om1-t4e	AA2AR	0.7	0.62	0.78	0.49	0.46	0.5	0.56	0.86	0.79	0.61	0.64	0.78	0.83	0.73	0.73	0.63	0.67	0.6	0.73	0.79	1	0.79	0.35	0.68	0.43	0.52	0.78	0.61
T4E	5om4-t4e	AA2AR	0.62	0.65	0.69	0.38	0.48	0.39	0.4	0.75	1	0.64	0.67	0.69	0.73	0.64	0.64	0.66	0.6	0.63	0.68	1	0.93	1	0.38	0.58	0.46	0.48	0.69	0.53
DU1	5uen-du1	AA2AR	0.53	0.39	0.47	0.65	0.41	0.35	0.22	0.19	0.4	0.5	0.36	0.47	0.32	0.54	0.54	0.64	0.67	0.37	0.56	0.4	0.44	0.4	1	0.47	0.39	0.41	0.47	0.21
ZMA	5uvi-zma	AA2AR	0.48	0.43	0.81	0.48	0.26	0.18	0.6	0.41	0.36	0.37	0.32	0.81	0.71	0.9	0.9	0.47	0.49	0.41	0.34	0.36	0.49	0.36	0.27	1	0.19	0.26	0.81	0.4
UKA	5wf5-uka	AA2AR	0.64	0.77	0.59	0.45	0.95	0.46	-0.04	0.46	0.68	0.89	0.77	0.59	0.45	0.53	0.53	0.74	0.57	0.68	0.73	0.68	0.73	0.68	0.52	0.46	1	0.88	0.59	0.68
UKA	5wf6-uka	AA2AR	0.73	0.66	0.68	0.44	0.86	0.44	0.12	0.57	0.62	0.73	0.68	0.68	0.56	0.62	0.62	0.78	0.64	0.62	0.7	0.62	0.8	0.62	0.49	0.56	0.79	1	0.68	0.65
ZMA	6aqf-zma	AA2AR	0.57	0.51	1	0.41	0.37	0.41	0.73	0.53	0.49	0.49	0.44	1	0.93	0.97	0.97	0.61	0.54	0.57	0.5	0.49	0.65	0.49	0.31	0.93	0.29	0.37	1	0.49
NEC	6gdg-nec	AA2AR	0.68	0.71	0.54	0.14	0.46	0.26	0.62	0.59	0.42	0.51	0.55	0.54	0.58	0.57	0.57	0.39	0.36	0.19	0.5	0.42	0.56	0.42	0.17	0.51	0.37	0.39	0.54	1
CAU	2rh1-cau	ADRB2	0.27	0.24	0.04	-0.19	0.13	-0.35	-0.26	-0.19	0.06	0.1	0.24	0.04	-0.01	0.07	0.07	0.15	0.03	-0.2	0.08	0.06	0.06	0.06	0.22	-0.03	0.07	0.14	0.04	0.1
TIM	3d4s-tim	ADRB2	0.27	0.24	0.04	-0.19	0.13	-0.35	-0.26	-0.19	0.06	0.1	0.24	0.04	-0.01	0.07	0.07	0.15	0.03	-0.2	0.08	0.06	0.06	0.06	0.22	-0.03	0.07	0.14	0.04	0.1
JRZ	3ny8-jrz	ADRB2	0.36	0.29	0.15	-0.05	0.18	-0.21	-0.04	-0.08	0.1	0.16	0.28	0.15	0.09	0.2	0.2	0.21	0.12	-0.13	0.18	0.1	0.13	0.1	0.28	0.1	0.12	0.19	0.15	0.18
JSZ	3ny9-jsz	ADRB2	0.28	0.27	0.04	-0.18	0.15	-0.34	-0.25	-0.18	0.09	0.13	0.26	0.04	-0.11	0.08	0.08	0.15	0.04	-0.2	0.09	0.09	0.07	0.09	0.23	-0.03	0.09	0.13	0.04	0.11
JTZ	3nya-jtz	ADRB2	0.3	0.24	0.1	-0.12	0.14	-0.28	-0.15	-0.15	0.06	0.12	0.24	0.1	0.03	0.13	0.13	-0.14	0.08	-0.18	0.13	0.06	0.09	0.06	0.24	0.03	0.09	0.09	0.1	0.13
CAU	5d6l-cau	ADRB2	0.27	0.24	0.04	-0.19	0.13	-0.35	-0.26	-0.19	0.06	0.1	0.24	0.04	-0.01	0.07	0.07	0.15	0.03	-0.2	0.08	0.06	0.06	0.06	0.22	-0.03	0.07	0.07	0.04	0.1
QNB	3uon-qnb	ACM2	0.18	0.13	0.04	-0.08	0.08	-0.35	-0.26	-0.19	0.03	0.01	0.11	0.04	-0.12	0.07	0.07	0.15	0.03	-0.12	0.01	0.03	-0.02	0.03	0.22	0.08	0.03	0.15	0.04	0.22
OHK	5cxv-0hk	ACM2	0.07	0.01	-0.09	-0.22	0.09	-0.32	-0.44	-0.31	-0.06	-0.08	0	-0.09	-0.12	-0.06	-0.06	0.06	-0.07	-0.23	-0.07	-0.06	-0.12	-0.06	0.14	-0.07	0.04	0.09	-0.09	0.13
OHK	5dsg-0hk	ACM4	0.04	0.04	-0.12	-0.26	0.08	-0.35	-0.49	-0.34	-0.13	-0.06	0.06	-0.12	-0.15	-0.09	-0.09	0.12	-0.09	-0.3	-0.1	-0.13	-0.15	-0.13	0.08	-0.1	0.03	0.11	-0.12	0.11
2GM	4iaq-2gm	5HT1B	0.18	0.21	-0.18	-0.3	0.25	-0.23	-0.44	-0.27	0.13	0.12	0.16	-0.18	-0.23	-0.17	-0.17	0.14	-0.05	-0.3	0.03	0.13	0.01	0.13	0.14	-0.29	0.18	0.26	-0.18	0.16
ERM	4iar-erm	5HT1B	0.16	0.19	-0.21	-0.33	0.23	-0.27	-0.49	-0.31	0.1	0.1	0.14	-0.21	-0.26	-0.2	-0.2	0.12	-0.07	-0.2	0.01	0.1	-0.02	0.1	0.12	-0.32	0.24	0.25	-0.21	0.13
ERM	4ib4-erm	5HT2B	0.19	0.14	0.11	-0.28	0.27	-0.26	-0.18	-0.23	0.06	0.12	0.08	0.11	-0.07	0.12	0.12	0.36	-0.04	0.07	0.15	0.06	0.01	0.06	0.17	0.02	0.28	0.25	0.11	0.17
ERM	4nc3-erm	5HT2B	0.22	0.16	0.14	-0.25	0.28	-0.23	-0.12	-0.19	0.08	0.14	0.1	0.14	-0.03	0.15	0.15	0.38	-0.01	0.1	0.17	0.08	0.04	0.08	0.19	0.05	0.29	0.27	0.14	0.19
ERM	5tud-erm	5HT2B	0.23	0.17	0.15	-0.23	0.28	0.03	-0.1	-0.17	0.07	0.15	0.09	0.15	0.02	0.17	0.17	0.3	0	0.11	0.18	0.07	0.05	0.07	0.2	0.07	0.28	0.28	0.15	0.21
7LD	5tvn-7ld	5HT2B	0.39	0.38	0.19	-0.04	0.21	-0.2	0.27	0.04	0.19	0.24	0.29	0.19	0.18	0.24	0.24	0.21	0.13	0.01	0.19	0.19	0.19	0.19	0.2	0.14	0.15	0.18	0.19	0.36
H8G	6drx-h8g	5HT2B	0.3	0.31	0.1	0	0.32	-0.12	0.1	-0.08	0.12	0.18	0.23	0.1	0.07	0.13	0.13	0.35	0.16	-0.07	0.24	0.12	0.11	0.12	0.26	0.03	0.24	0.33	0.1	0.27
H8D	6dry-h8d	5HT2B	0.18	0.21	0.22	-0.12	0.32	-0.09	0.08	-0.05	0.05	0.18	0.14	0.22	0.06	0.24	0.24	0.37	0.05	0.05	0.16	0.05	0.02	0.05	0.16	0.14	0.24	0.33	0.22	0.27
H8J	6drz-h8j	5HT2B	0.33	0.25	0.25	0.03	0.28	0.07	0.33	0.11	0.16	0.13	0.16	0.25	0.25	0.27	0.27	0.39	0.18	0.05	0.29	0.16	0.16	0.16	0.2	0.18	0.21	0.26	0.25	0.41
H8M	6ds0-h8m	5HT2B	0.21	0.21	0.25	0.37	0.26	0.42	0.38	0.33	0.26	0.26	0.2	0.25	0.25	0.27	0.27	0.39	0.3	0.13	0.48	0.26	-0.08	0.26	0.22	0.18	0.25	0.22	0.25	0.17
AZJ	4ntj-azj	P2Y12	-0.13	-0.05	-0.17	-0.5	-0.15	-0.37	-0.4	-0.37	-0.13	-0.12	-0.01	-0.17	-0.21	-0.15	-0.15	-0.14	-0.18	-0.35	-0.19	-0.13	-0.19	-0.13	-0.17	-0.27	-0.18	-0.13	-0.17	-0.13
6AD	4pxz-6ad	P2Y12	-0.32	-0.15	-0.37	-0.69	-0.18	-0.68	-0.77	-0.18	-0.27	-0.2	-0.1	-0.37	-0.5	-0.36	-0.36	-0.35	-0.34	-0.51	-0.38	-0.27	-0.36	-0.27	-0.28	-0.5	-0.2	-0.23	-0.37	-0.15
1Q5	4z9g-1q5	CRF1	-0.01	0	-0.13	-0.27	-0.04	-0.37	-0.04	-0.14	-0.13	0.26	-0.06	-0.13	-0.2	-0.11	-0.11	-0.02	-0.15	-0.1	-0.08	-0.13	-0.08	-0.13	-0.08	-0.23	-0.02	-0.1	-0.13	-0.06
73R	5t1a-73r	CCR2	-0.38	-0.37	-0.35	-0.46	-0.28	-0.52	-0.63	-0.52	-0.43	-0.27	-0.35	-0.35	-0.5	-0.35	-0.35	-0.32	-0.23	-0.37	-0.42	-0.43	-0.43	-0.43	-0.2	-0.37	-0.29	-0.28	-0.35	-0.37
8ES	5unf-8es	AGTR2	-0.05	0.01	-0.29	-0.34	0.06	-0.24	-0.56	-0.44	-0.07	-0.03	-0.01	-0.29	-0.34	-0.28	-0.28	0.03	0.05	-0.32	0	-0.07	-0.16	-0.07	0.17	-0.41	0.04	0.01	-0.29	-0.08
8EM	5unh-8em	AGTR2	0.01	0.06	-0.22	-0.3	0.08	-0.05	-0.49	-0.24	-0.04	0.07	0.07	-0.22	-0.26	-0.24	-0.24	0.05	0.08	-0.26	0.13	-0.04	-0.02	-0.04	0.14	-0.36	0.06	0.05	-0.22	-0.02
AQD	5wiv-aqd	DRD4	-0.01	0.05	-0.24	-0.21	0.17	-0.17	-0.77	-0.25	0.16	0.13	0.16	-0.24	-0.47	-0.22	-0.22	0.09	0.01	-0.14	0.18	0.16	0.02	0.16	0.28	-0.35	0.24	0.2	-0.24	-0.05
RET	6fuf-ret	GNAO	0.01	0.07	-0.09	-0.37	-0.06	-0.27	-0.19	-0.27	-0.07	-0.02	0	-0.09	-0.11	-0.06	-0.06	-0.13	-0.12	-0.28	-0.12	-0.07	-0.12	-0.07	-0.05	-0.18	-0.1	-0.06	-0.09	0.01

Target Ligand v	Target Pdb v	Protein	CAU	TIM	JRZ	JSZ	JTZ	CAU	QNB	OHK	OHK	2GM	ERM	ERM	ERM	ERM	7LD	H8G	H8D	H8J	H8M	AZJ	6AD	1Q5	73R	8ES	8EM	AQD	RET
	Ref. Pdb >		2rh1	3d4s	3ny8	3ny9	3nya	5d6l	3uon	5cxv	5dsg	4iaq	4iar	4ib4	4nc3	5tud	5tvn	6drx	6dry	6drz	6ds0	4ntj	4pxz	4z9g	5t1a	5unf	5unh	5wiv	6fuf
		Protein	ADRB2						ACM2		ACM4	5HT1B		5HT2B								P2Y12		CRF1	CCR2	AGTR2		DRD4	GNAO
ADN	2ydo-adn	AA2AR	0.17	0.17	0.3	0.15	0.22	0.17	0.11	0.04	-0.01	0.11	0.11	0.1	0.14	0.18	0.26	0.21	0.14	0.24	0.16	-0.09	-0.17	-0.06	-0.29	-0.03	0	0	0.02
NEC	2ydv-nec	AA2AR	0.18	0.18	0.29	0.18	0.21	0.18	0.07	-0.03	0.03	0.15	0.16	0.07	0.11	0.18	0.31	0.26	0.19	0.22	0.19	-0.04	-0.09	-0.08	-0.34	0	0.03	0.03	0.14
ZMA	3eml-zma	AA2AR	0.02	0.02	0.12	0.02	0.06	0.02	0.02	-0.06	-0.11	-0.09	-0.06	0.07	0.1	0.13	0.12	0.06	0.16	0.17	0.18	-0.11	-0.17	-0.14	-0.29	-0.15	-0.1	-0.11	-0.14
ZMA	3pwh-zma	AA2AR	-0.09	-0.09	-0.03	-0.08	-0.07	-0.09	-0.04	-0.12	-0.17	-0.14	-0.13	-0.12	-0.11	-0.16	-0.02	0	-0.1	-0.01	0.2	-0.28	-0.28	-0.23	-0.33	-0.15	-0.15	-0.08	-0.49
UKA	3qak-uka	AA2AR	0.15	0.15	0.25	0.15	0.18	0.15	0.1	0.1	0.07	0.25	0.25	0.22	0.25	0.39	0.25	0.38	0.42	0.35	0.34	-0.18	-0.15	-0.07	-0.37	0.06	0.07	0.15	-0.18
XAC	3rey-xac	AA2AR	-0.16	-0.16	-0.13	-0.14	-0.15	-0.16	-0.16	-0.16	-0.17	-0.1	-0.08	-0.08	-0.06	0.04	-0.1	-0.06	-0.05	0.04	0.25	-0.2	-0.26	-0.28	-0.35	-0.1	-0.02	-0.06	-0.34
CFF	3rfm-cff	AA2AR	-0.08	-0.08	-0.02	-0.07	-0.05	-0.08	-0.08	-0.17	-0.2	-0.13	-0.12	-0.04	-0.01	-0.03	0.09	0.03	0.03	0.12	0.15	-0.14	-0.19	-0.04	-0.28	-0.15	-0.13	-0.19	-0.16
T4G	3uza-t4g	AA2AR	-0.09	-0.09	-0.05	-0.07	-0.08	-0.09	-0.09	-0.18	-0.21	-0.12	-0.1	-0.07	-0.04	-0.07	0.02	-0.04	-0.03	0.06	0.2	-0.2	-0.25	-0.12	-0.32	-0.18	-0.09	-0.1	-0.34
T4E	3uzc-t4e	AA2AR	0.05	0.05	0.11	0.06	0.06	0.05	0.03	-0.05	-0.12	0.09	0.11	0.03	0.06	0.09	0.17	0.07	0.01	0.16	0.26	-0.12	-0.18	-0.24	-0.47	-0.05	-0.03	0.1	-0.16
NGL	4ug2-ngi	AA2AR	0.09	0.09	0.2	0.1	0.13	0.09	-0.02	-0.12	-0.06	0.1	0.11	0.08	0.11	0.19	0.24	0.18	0.2	0.14	0.29	-0.12	-0.15	-0.18	-0.3	-0.03	0.05	0.09	-0.05
NGI	4uhr-ngi	AA2AR	0.2	0.2	0.32	0.2	0.24	0.2	0.09	0	0.06	0.13	0.14	0.04	0.08	0.12	0.27	0.22	0.15	0.16	0.22	-0.01	-0.07	-0.13	-0.38	-0.01	0.05	0.11	0
ZMA	5k2a-zma	AA2AR	0.02	0.02	0.12	0.02	0.06	0.02	0.02	-0.06	-0.11	-0.09	-0.06	0.07	0.1	0.13	0.12	0.06	0.16	0.17	0.18	-0.11	-0.17	-0.14	-0.29	-0.15	-0.1	-0.11	-0.14
ZMA	5k2b-zma	AA2AR	0	0	0.06	-0.05	0.01	0	-0.06	-0.06	-0.08	-0.11	-0.08	-0.01	0.02	0.04	0.1	0.04	0.04	0.15	0.15	-0.12	-0.2	-0.17	-0.35	-0.15	-0.11	-0.19	-0.15
ZMA	5k2c-zma	AA2AR	0.04	0.04	0.14	0.04	0.08	0.04	0.04	-0.04	-0.09	-0.09	-0.06	0.07	0.1	0.13	0.14	0.08	0.16	0.17	0.18	-0.09	-0.16	-0.12	-0.27	-0.13	-0.1	-0.1	-0.09
ZMA	5k2d-zma	AA2AR	0.04	0.04	0.14	0.04	0.08	0.04	0.04	-0.04	-0.09	-0.09	-0.06	0.07	0.1	0.13	0.14	0.08	0.16	0.17	0.18	-0.09	-0.16	-0.12	-0.27	-0.13	-0.1	-0.1	-0.09
ZMA	5olg-zma	AA2AR	0.12	0.12	0.23	0.11	0.16	0.12	0.12	0.03	0.1	0.11	0.12	0.25	0.3	0.31	0.19	0.32	0.37	0.38	0.39	-0.13	-0.23	-0.05	-0.37	0.02	0.05	0.06	-0.27
9XT	5olh-9xt	AA2AR	0.02	0.02	0.12	0.02	0.06	0.02	0.02	-0.06	-0.11	-0.03	-0.01	-0.02	0.01	0	0.1	0.13	0.02	0.13	0.25	-0.15	-0.2	-0.19	-0.24	0.03	0.02	0.01	-0.23
9XW	5olo-9xw	AA2AR	-0.14	-0.14	-0.12	-0.12	-0.14	-0.14	-0.08	-0.17	-0.22	-0.19	-0.11	0.04	0.06	0.09	0	-0.06	0.05	0.05	0.11	-0.27	-0.29	-0.11	-0.37	-0.19	-0.15	-0.08	-0.52
92Y	5olv-92y	AA2AR	0.06	0.06	0.17	0.06	0.11	0.06	0	-0.08	-0.11	0.02	0.04	0.1	0.14	0.18	0.14	0.19	0.14	0.24	0.42	-0.15	-0.22	-0.11	-0.43	0	0.08	0.1	-0.23
T4E	5olz-t4e	AA2AR	0.05	0.05	0.11	0.06	0.06	0.05	0.03	-0.05	-0.12	0.09	0.11	0.03	0.06	0.09	0.17	0.07	0.01	0.16	0.26	-0.12	-0.18	-0.24	-0.47	-0.05	-0.03	0.1	-0.16
T4E	5om1-t4e	AA2AR	0.04	0.04	0.13	0.04	0.07	0.04	-0.01	-0.1	-0.14	0	0.02	0.02	0.06	0.07	0.14	0.09	0.02	0.13	0.23	-0.15	-0.2	-0.11	-0.4	-0.1	-0.01	0.01	-0.23
T4E	5om4-t4e	AA2AR	0.05	0.05	0.11	0.06	0.06	0.05	0.03	-0.05	-0.12	0.09	0.11	0.03	0.06	0.09	0.17	0.07	0.01	0.16	0.26	-0.12	-0.18	-0.24	-0.47	-0.05	-0.03	0.1	-0.16
DU1	5uen-du1	AA2AR	0.19	0.19	0.33	0.18	0.25	0.19	0.19	0.13	0.07	0.11	0.11	0.09	0.13	0.17	0.19	0.2	0.13	0.14	0.17	-0.17	-0.21	-0.16	-0.25	0.09	0.05	0.19	-0.11
ZMA	5uvi-zma	AA2AR	-0.02	-0.02	0.07	-0.01	0.02	-0.02	0.04	-0.04	-0.09	-0.14	-0.11	0.02	0.06	0.07	0.08	0.02	0.09	0.11	0.11	-0.15	-0.2	-0.2	-0.27	-0.18	-0.15	-0.14	-0.24
UKA	5wf5-uka	AA2AR	0.1	0.1	0.18	0.11	0.12	0.1	0.05	0.05	0.01	0.2	0.26	0.22	0.26	0.4	0.19	0.32	0.35	0.29	0.37	-0.24	-0.19	-0.04	-0.45	0.04	0.06	0.23	-0.32
UKA	5wf6-uka	AA2AR	0.17	0.17	0.28	0.14	0.2	0.17	0.17	0.09	0.14	0.26	0.27	0.23	0.27	0.39	0.26	0.4	0.44	0.33	0.27	-0.16	-0.2	-0.21	-0.37	0.01	0.04	0.15	-0.18
ZMA	6aqf-zma	AA2AR	0.02	0.02	0.12	0.02	0.06	0.02	0.02	-0.06	-0.11	-0.09	-0.06	0.07	0.1	0.21	0.12	0.06	0.16	0.17	0.18	-0.11	-0.17	-0.14	-0.29	-0.15	-0.1	-0.11	-0.14
NEC	6gdg-nec	AA2AR	0.06	0.1	0.15	0.09	0.13	0.06	0.14	0.07	0.03	0.09	0.11	0.1	0.14	0.18	0.25	0.2	0.21	0.31	0.1	-0.09	-0.08	-0.08	-0.29	-0.04	-0.01	-0.03	0.02
CAU	2rh1-cau	ADRB2	1	0.92	0.91	0.85	0.87	1	0.49	0.43	0.41	0.51	0.53	0.25	0.21	0.25	0.5	0.46	0.26	0.29	0.3	0.05	-0.11	-0.08	-0.51	-0.01	-0.05	0.28	-0.1
TIM	3d4s-tim	ADRB2	0.92	1	0.91	0.85	0.96	0.92	0.54	0.48	0.46	0.51	0.53	0.24	0.2	0.25	0.52	0.48	0.26	0.29	0.3	-0.03	-0.11	-0.1	-0.49	-0.01	-0.05	0.22	-0.1
JRZ	3ny8-jrz	ADRB2	0.66	0.66	1	0.55	0.82	0.66	0.43	0.36	0.35	0.35	0.38	0.28	0.24	0.31	0.42	0.42	0.32	0.39	0.25	0.04	-0.12	-0.14	-0.41	0.07	0.02	0.27	0.08
JSZ	3ny9-jsz	ADRB2	0.95	0.95	0.84	1	0.9	0.95	0.52	0.46	0.48	0.55	0.56	0.28	0.24	0.25	0.53	0.49	0.26	0.29	0.31	0.06	-0.02	0.07	-0.56	0	-0.05	0.37	-0.09
JTZ	3nya-jtz	ADRB2	0.74	0.82	0.95	0.68	1	0.74	0.46	0.4	0.38	0.44	0.46	0.27	0.23	0.29	0.52	0.52	0.28	0.35	0.35	0	-0.08	-0.05	-0.44	0.04	-0.01	0.25	-0.01
CAU	5d6l-cau	ADRB2	1	0.92	0.91	0.85	0.87	1	0.49	0.43	0.41	0.51	0.53	0.25	0.21	0.25	0.5	0.46	0.26	0.29	0.3	0.05	-0.11	-0.08	-0.51	-0.01	-0.05	0.28	-0.1
QNB	3uon-qnb	ACM2	0.48	0.54	0.59	0.46	0.54	0.48	1	0.98	0.94	0.38	0.4	0.3	0.36	0.38	0.32	0.39	0.33	0.45	0.1	0.07	-0.01	-0.16	-0.46	0.02	-0.07	0.25	-0.22
OHK	5cxv-0hk	ACM2	0.39	0.45	0.46	0.38	0.44	0.39	0.91	1	0.94	0.4	0.42	0.31	0.37	0.4	0.22	0.41	0.35	0.47	0.12	0.07	-0.01	-0.14	-0.44	0.04	-0.05	0.27	-0.17
OHK	5dsg-0hk	ACM4	0.39	0.45	0.46	0.42	0.44	0.39	0.91	0.98	1	0.38	0.4	0.3	0.36	0.38	0.28	0.39	0.33	0.45	0.1	0.13	-0.01	-0.07	-0.46	0.02	-0.07	0.25	-0.06
2GM	4iaq-2gm	5HT1B	0.54	0.54	0.51	0.52	0.55	0.54	0.4	0.45	0.41	1	0.95	0.56	0.57	0.63	0.72	0.67	0.52	0.58	0.43	-0.01	-0.14	0.01	-0.31	0.12	0.03	0.4	-0.05
ERM	4iar-erm	5HT1B	0.58	0.58	0.57	0.55	0.6	0.58	0.44	0.5	0.45	0.98	1	0.61	0.6	0.68	0.76	0.72	0.5	0.63	0.48	-0.03	-0.11	0.07	-0.34	0.11	0.01	0.44	-0.09
ERM	4ib4-erm	5HT2B	0.32	0.3	0.48	0.34	0.39	0.32	0.35	0.4	0.38	0.66	0.69	1	0.97	0.84	0.63	0.64	0.71	0.82	0.41	-0.09	-0.11	0.08	-0.35	0.12	0.09	0.4	-0.27
ERM	4nc3-erm	5HT2B	0.24	0.22	0.37	0.28	0.3	0.24	0.39	0.44	0.41	0.6	0.62	0.88	1	0.79	0.55	0.67	0.76	0.86	0.45	-0.05	-0.09	0.02	-0.3	0.15	0.11	0.34	-0.18
ERM	5tud-erm	5HT2B	0.21	0.21	0.35	0.19	0.28	0.21	0.32	0.36	0.33	0.49	0.51	0.56	0.58	1	0.56	0.5	0.57	0.68	0.42	-0.04	-0.18	0	-0.16	0.15	0.13	0.27	-0.07
7LD	5tvn-7ld	5HT2B	0.46	0.47	0.52	0.41	0.56	0.46	0.3	0.22	0.27	0.61	0.63	0.46	0.44	0.61	1	0.73	0.57	0.68	0.54	0.04	-0.11	0.06	-0.27	0.07	0.04	0.24	0.09
H8G	6drx-h8g	5HT2B	0.41	0.42	0.5	0.37	0.54	0.41	0.36	0.4	0.33	0.55	0.57	0.45	0.52	0.53	0.71	1	0.67	0.77	0.55	0.04	-0.11	0.14	-0.49	0.17	0.07	0.29	0.09
H8D	6dry-h8d	5HT2B	0.21	0.21	0.35	0.17	0.26	0.21	0.26	0.3	0.24	0.39	0.36	0.45	0.53	0.54	0.5	0.6	1	0.78	0.56	-0.08	-0.16	-0.01	-0.13	0.02	-0.01	0.15	0.14
H8J	6drz-h8j	5HT2B	0.24	0.24	0.44	0.22	0.34	0.24	0.37	0.42	0.36	0.45	0.48	0.54	0.63	0.67	0.62	0.73	0.81	1	0.58	0	-0.15	-0.06	-0.24	0.12	0.08	0.22	-0.02
H8M	6ds0-h8m	5HT2B	0.24	0.24	0.27	0.22	0.32	0.24	0.06	0.05	0.02	0.32	0.34	0.22	0.27	0.4	0.47	0.5	0.56	0.55	1	-0.1	-0.15	0.03	-0.17	0	0.02	0.17	-0.07
AZJ	4ntj-azj	P2Y12	0.08	0.01	0.05	0.08	0.05	0.08	0.06	0.06	0.12	0.01	-0.01	-0.06	-0.04	-0.05	0.06	0.07	-0.09	-0.01	-0.02	1	0.43	-0.11	-0.31	0.09	0.09	0	0.2
6AD	4pxz-6ad	P2Y12	-0.08	-0.09	-0.16	0.02	-0.06	-0.08	-0.01	-0.01	-0.07	-0.16	-0.12	-0.16	-0.14	-0.26	-0.13	-0.16	-0.25	-0.22	-0.19	0.57	1	-0.37	-0.5	-0.08	-0.06	-0.02	-0.11
1Q5	4z9g-1q5	CRF1	-0.02	-0.02	-0.06	0.07	0.02	-0.02	-0.09	-0.1	-0.03	0.02	0.04	0.04	0.01	0	0.08	0.14	-0.01	-0.01	0.07	-0.12	-0.16	1	-0.52	-0.08	-0.15	-0.12	0.02
73R	5t1a-73r	CCR2	-0.32	-0.32	-0.39	-0.33	-0.34	-0.32	-0.29	-0.29	-0.3	-0.19	-0.19	-0.2	-0.19	-0.13	-0.19	-0.37	-0.12	-0.2	-0.15	-0.25	-0.28	-0.55	1	0.01	0.03	-0.28	-0.66
8ES	5unf-8es	AGTR2	-0.01	-0.01	0.1	0	0.05	-0.01	0.03	0.05	0.03	0.13	0.11	0.11	0.15	0.21	0.09	0.22	0.03	0.16	0.05	0.11	-0.08	-0.14	0.02	1	0.85	0.2	-0.16
8EM	5unh-8em	AGTR2	-0.06	-0.06	0.03	-0.05	-0.01	-0.06	-0.08	-0.07	-0.08	0.03	0.02	0.08	0.12	0.19	0.05	0.1	-0.01	0.12	0.07	0.12	-0.05	-0.27	0.05	0.91	1	0.2	-0.25
AQD	5wiv-aqd	DRD4	0.39	0.33	0.5	0.46	0.43	0.39	0.31	0.35	0.31	0.47	0.49	0.39	0.36	0.39	0.36	0.41	0.23	0.32	0.27	0	-0.02	-0.26	-0.5	0.21	0.17	1	-0.23
RET	6fuf-ret	GNAO	-0.04	0.01	0.04	0.01	0.05	-0.04	-0.08	-0.09	-0.03	-0.01	-0.02	-0.07	-0.05	-0.02	0.05	0.05	0.06	-0.01	0.03	0.09	-0.03	0.01	-0.35	-0.05	-0.08	-0.05	1

Figure S9: Matrix of the normalized scores for the 10 digit profiling scheme using BLOSUM62 Scoring with a miss and gap penalty of -2. Each element has been coloured following a RdYlGn palette, where higher scores are green in colour, while the lower scores are red. The elements coloured in azure are all values that are >0.5 as these are scores that are of interest.

GPCRtm score with -2.0 miss - 8 digit profile

Target Ligand	Target Pdb	Protein	ADN	NEC	ZMA	ZMA	UKA	XAC	CFF	T4G	T4E	NGL	NGI	ZMA	ZMA	ZMA	ZMA	ZMA	9XT	9XW	92Y	T4E	T4E	T4E	DU1	ZMA	UKA	UKA	ZMA	NEC
	Ref. Pdb >		2ydo	2ydv	3eml	3pwh	3qak	3rey	3rfm	3uza	3uzc	4ug2	4uhr	5k2a	5k2b	5k2c	5k2d	5olg	5olh	5olo	5olv	5olz	5om1	5om4	5uen	5uvi	5wf5	5wf6	6aqf	6gdg
		Protein	AA2AR																											
ADN	2ydo-adn	AA2AR	1	0.74	0.54	0.4	0.39	0.11	0.59	0.38	0.47	0.51	0.56	0.54	0.56	0.58	0.58	0.4	0.42	0.31	0.33	0.41	0.58	0.41	0.43	0.51	0.3	0.39	0.54	0.61
NEC	2ydv-nec	AA2AR	0.9	1	0.6	0.27	0.58	0.17	0.38	0.45	0.6	0.72	0.79	0.6	0.62	0.64	0.64	0.47	0.47	0.38	0.38	0.56	0.62	0.56	0.38	0.57	0.47	0.39	0.6	0.79
ZMA	3eml-zma	AA2AR	0.5	0.45	1	0.56	0.33	0.43	0.66	0.47	0.49	0.44	0.38	1	0.92	0.96	0.96	0.61	0.59	0.66	0.44	0.44	0.61	0.44	0.36	0.92	0.24	0.33	1	0.39
ZMA	3pwh-zma	AA2AR	0.31	0.17	0.47	1	0.12	0.51	0.52	0.4	0.24	0.3	0.24	0.47	0.48	0.4	0.4	0.32	0.52	0.39	0.38	0.3	0.45	0.3	0.31	0.47	0.13	0.14	0.47	0.05
UKA	3qak-uka	AA2AR	0.68	0.82	0.61	0.4	1	0.3	-0.1	0.32	0.63	0.84	0.81	0.61	0.46	0.55	0.55	0.77	0.47	0.5	0.48	0.59	0.65	0.59	0.42	0.47	0.84	0.85	0.61	0.71
XAC	3rey-xac	AA2AR	0.08	0.11	0.35	0.5	0.13	1	0.45	0.55	0.23	0.24	0.18	0.35	0.5	0.26	0.26	0.34	0.41	0.3	0.62	0.24	0.38	0.24	0.12	0.16	0.14	0.15	0.35	0.19
CFF	3rfm-cff	AA2AR	0.27	0.14	0.33	0.31	-0.03	0.28	1	0.45	0.15	0.03	0.06	0.33	0.35	0.4	0.4	0.12	0.23	0.15	0.21	0.09	0.19	0.09	0.11	0.31	-0.07	-0.03	0.33	0.24
T4G	3uza-t4g	AA2AR	0.29	0.33	0.39	0.4	0.14	0.55	0.72	1	0.43	0.24	0.26	0.39	0.42	0.42	0.42	0.26	0.53	0.41	0.51	0.4	0.57	0.4	0.13	0.33	0.14	0.21	0.39	0.4
T4E	3uzc-t4e	AA2AR	0.55	0.61	0.63	0.52	0.45	0.43	0.24	0.7	1	0.6	0.63	0.63	0.67	0.57	0.57	0.68	0.75	0.7	0.7	1	0.91	1	0.42	0.49	0.43	0.41	0.63	0.44
NGL	4ug2-ngi	AA2AR	0.77	0.89	0.72	0.58	0.74	0.49	0.1	0.49	0.71	1	0.88	0.72	0.58	0.66	0.66	0.66	0.59	0.61	0.62	0.68	0.77	0.68	0.6	0.59	0.68	0.57	0.72	0.66
NGI	4uhr-ngi	AA2AR	0.81	0.92	0.6	0.44	0.67	0.34	0.17	0.49	0.71	0.83	1	0.6	0.62	0.53	0.53	0.57	0.59	0.49	0.51	0.68	0.77	0.68	0.4	0.45	0.55	0.5	0.6	0.69
ZMA	5k2a-zma	AA2AR	0.5	0.45	1	0.56	0.33	0.43	0.66	0.47	0.49	0.44	0.38	1	0.92	0.96	0.96	0.61	0.69	0.66	0.44	0.44	0.61	0.44	0.36	0.92	0.24	0.33	1	0.39
ZMA	5k2b-zma	AA2AR	0.44	0.39	0.77	0.48	0.21	0.51	0.59	0.43	0.45	0.3	0.34	0.77	1	0.72	0.72	0.44	0.62	0.49	0.41	0.39	0.55	0.39	0.2	0.65	0.13	0.21	0.77	0.35
ZMA	5k2c-zma	AA2AR	0.5	0.45	0.89	0.44	0.27	0.3	0.72	0.47	0.42	0.37	0.31	0.89	0.79	1	1	0.53	0.59	0.58	0.35	0.37	0.52	0.37	0.38	0.96	0.19	0.27	0.89	0.39
ZMA	5k2d-zma	AA2AR	0.5	0.45	0.89	0.44	0.27	0.3	0.72	0.47	0.42	0.37	0.31	0.89	0.79	1	1	0.53	0.59	0.58	0.35	0.37	0.52	0.37	0.38	0.96	0.19	0.27	0.89	0.39
ZMA	5olg-zma	AA2AR	0.5	0.47	0.82	0.52	0.84	0.55	0.31	0.43	0.65	0.54	0.49	0.82	0.71	0.77	0.77	1	0.66	0.74	0.67	0.63	0.72	0.63	0.52	0.71	0.44	0.53	0.82	0.39
9XT	5olh-9xt	AA2AR	0.44	0.39	0.77	0.74	0.28	0.55	0.52	0.72	0.54	0.4	0.43	0.77	0.83	0.72	0.72	0.55	1	0.7	0.63	0.59	0.78	0.59	0.42	0.65	0.27	0.36	0.77	0.32
9XW	5olo-9xw	AA2AR	0.37	0.37	0.86	0.6	0.36	0.47	0.38	0.64	0.54	0.48	0.4	0.86	0.75	0.81	0.81	0.71	0.81	1	0.57	0.63	0.72	0.63	0.48	0.76	0.4	0.4	0.86	0.26
92Y	5olv-92y	AA2AR	0.34	0.32	0.49	0.5	0.28	0.83	0.45	0.68	0.59	0.41	0.36	0.49	0.54	0.42	0.42	0.55	0.62	0.49	1	0.54	0.7	0.54	0.31	0.33	0.34	0.36	0.49	0.45
T4E	5olz-t4e	AA2AR	0.55	0.61	0.63	0.52	0.45	0.43	0.24	0.7	1	0.6	0.63	0.63	0.67	0.57	0.57	0.68	0.75	0.7	0.7	1	0.91	1	0.42	0.49	0.43	0.41	0.63	0.44
T4E	5om1-t4e	AA2AR	0.65	0.57	0.74	0.65	0.42	0.55	0.45	0.83	0.79	0.56	0.6	0.74	0.79	0.68	0.68	0.65	0.84	0.68	0.76	0.77	1	0.77	0.39	0.61	0.39	0.5	0.74	0.53
T4E	5om4-t4e	AA2AR	0.55	0.61	0.63	0.52	0.45	0.43	0.24	0.75	1	0.6	0.63	0.63	0.67	0.57	0.57	0.68	0.75	0.7	0.7	1	0.91	1	0.42	0.49	0.43	0.41	0.63	0.44
DU1	5uen-du1	AA2AR	0.53	0.42	0.53	0.54	0.33	0.21	0.31	0.23	0.4	0.53	0.38	0.53	0.35	0.6	0.6	0.57	0.55	0.54	0.41	0.43	0.48	0.43	1	0.53	0.32	0.33	0.53	0.19
ZMA	5uvi-zma	AA2AR	0.4	0.37	0.79	0.48	0.21	0.17	0.52	0.34	0.36	0.31	0.25	0.79	0.67	0.89	0.89	0.45	0.5	0.5	0.25	0.29	0.43	0.29	0.31	1	0.14	0.21	0.79	0.29
UKA	5wf5-uka	AA2AR	0.58	0.74	0.51	0.33	0.94	0.36	-0.31	0.36	0.68	0.87	0.74	0.51	0.33	0.43	0.43	0.69	0.5	0.65	0.65	0.63	0.68	0.63	0.45	0.35	1	0.87	0.51	0.61
UKA	5wf6-uka	AA2AR	0.68	0.62	0.61	0.31	0.84	0.34	-0.1	0.49	0.62	0.7	0.64	0.61	0.46	0.55	0.55	0.74	0.59	0.58	0.6	0.57	0.77	0.57	0.42	0.47	0.77	1	0.61	0.58
ZMA	6aqf-zma	AA2AR	0.5	0.45	1	0.56	0.33	0.43	0.66	0.47	0.49	0.44	0.38	1	0.92	0.96	0.96	0.61	0.69	0.66	0.44	0.44	0.61	0.44	0.36	0.92	0.24	0.33	1	0.39
NEC	6gdg-nec	AA2AR	0.6	0.64	0.54	0.06	0.41	0.26	0.52	0.53	0.42	0.44	0.48	0.42	0.46	0.45	0.45	0.31	0.31	0.22	0.44	0.33	0.48	0.33	0.13	0.37	0.32	0.34	0.42	1
CAU	2rh1-cau	ADRB2	0.35	0.33	0.14	-0.1	0.11	-0.15	0.31	0.02	0.06	0.19	0.31	0.14	0.1	0.19	0.19	0.16	0.05	0.03	0.14	0.15	0.13	0.15	0.2	0.08	0.12	0.13	0.14	0.19
TIM	3d4s-tim	ADRB2	0.35	0.33	0.14	-0.1	0.11	-0.15	0.31	0.02	0.06	0.19	0.31	0.14	0.1	0.19	0.19	0.13	0.05	0.03	0.14	0.15	0.13	0.15	0.2	0.08	0.12	0.13	0.14	0.34
JRZ	3ny8-jrz	ADRB2	0.29	0.22	0.21	-0.05	0.11	-0.15	0.1	-0.11	0.1	0.19	0.22	0.21	0.1	0.26	0.26	0.13	-0.02	0.05	0.05	0.05	0.07	0.05	0.23	0.16	0.12	0.13	0.21	0.1
JSZ	3ny9-jsz	ADRB2	0.29	0.28	0.07	-0.18	0.13	-0.28	0.1	-0.11	0.09	0.15	0.27	0.07	-0.02	0.11	0.11	0.13	-0.02	0.05	0.05	0.2	0.07	0.2	0.23	0	0.13	0.09	0.07	0.24
JTZ	3nya-jtz	ADRB2	0.23	0.17	0.14	-0.27	0.07	-0.23	-0.03	-0.19	0.06	0.15	0.18	0.14	0.02	0.19	0.19	0.08	-0.08	0	-0.02	0	0.01	0	0.18	0.08	0.08	0.09	0.14	0.18
CAU	5d6l-cau	ADRB2	0.35	0.33	0.14	-0.1	0.11	-0.15	0.31	0.02	0.06	0.19	0.31	0.14	0.1	0.19	0.19	0.16	0.05	0.03	0.14	0.15	0.13	0.15	0.2	0.08	0.12	0.13	0.14	0.19
QNB	3uon-qnb	ACM2	0.26	0.18	0.04	-0.06	0.13	-0.15	0.31	0.02	0.03	0.07	0.16	0.18	0.1	0.23	0.23	0.21	0.08	0.14	0.05	0.11	0.07	0.11	0.2	0.12	0.07	0.12	0.18	0.34
OHK	5cxv-0hk	ACM2	0.26	0.18	0.18	-0.06	0.13	-0.11	0.38	0.06	-0.06	0.07	0.09	0.18	0.15	0.23	0.23	0.13	0.08	0.14	0.08	0.09	0.07	0.09	0.2	0.12	0.07	0.13	0.18	0.37
OHK	5dsg-0hk	ACM4	0.26	0.18	0.18	-0.06	0.13	-0.15	0.31	0.02	-0.13	0.07	0.16	0.18	0.1	0.23	0.23	0.21	0.08	0.14	0.05	0.11	0.07	0.11	0.2	0.12	0.07	0.12	0.18	0.34
2GM	4iaq-2gm	5HT1B	0.32	0.36	0.07	-0.21	0.17	-0.21	-0.14	-0.02	0.13	0.17	0.22	0.07	0.04	-0.02	-0.02	0.16	0	0.05	0.1	0.1	0.09	0.1	0.12	-0.14	0.15	0.23	0.07	0.34
ERM	4iar-erm	5HT1B	0.4	0.42	0.16	-0.1	0.21	-0.11	0.03	0.09	0.1	0.22	0.28	0.16	0.15	0.08	0.08	0.22	0.08	0.09	0.17	0.16	0.16	0.16	0.18	-0.04	0.19	0.28	0.39	0.42
ERM	4ib4-erm	5HT2B	0.13	0.2	0.18	-0.27	0.26	-0.28	-0.24	-0.23	0.06	0.23	0.19	0.18	-0.02	0.08	0.08	0.34	-0.05	0.14	0.05	0.09	0.04	0.09	0.18	-0.04	0.23	0.31	0.18	0.15
ERM	4nc3-erm	5HT2B	0.19	0.25	0.25	-0.19	0.28	-0.19	-0.1	-0.15	0.08	0.28	0.24	0.25	0.06	0.15	0.15	0.39	0.02	0.16	0.11	0.13	0.1	0.13	0.23	0.04	0.25	0.33	0.25	0.21
ERM	5tud-erm	5HT2B	0.39	0.29	0.46	0.06	0.3	0.06	0.31	0.11	0.07	0.31	0.25	0.46	0.31	0.38	0.38	0.31	0.2	0.24	0.3	0.16	0.28	0.16	0.27	0.29	0.27	0.3	0.46	0.4
7LD	5tvn-7ld	5HT2B	0.35	0.36	0.12	-0.1	0.2	-0.15	0.31	0.02	0.19	0.2	0.26	0.12	0.1	0.17	0.17	0.19	0.05	0.09	0.14	0.13	0.13	0.13	0.26	0.06	0.19	0.26	0.12	0.34
H8G	6drx-h8g	5HT2B	0.35	0.36	0.12	-0.1	0.26	-0.15	0.31	0.02	0.12	0.2	0.26	0.12	0.1	0.17	0.17	0.27	0.05	0.09	0.14	0.24	0.13	0.24	0.26	0.06	0.18	0.28	0.12	0.34
H8D	6dry-h8d	5HT2B	0.02	0.08	0.16	-0.08	0.21	-0.13	0.07	-0.09	0.05	0.14	0.09	0.16	-0.04	0.06	0.06	0.22	-0.06	0.12	-0.06	0.09	-0.06	0.09	0.1	-0.06	0.13	0.22	0.16	0.13
H8J	6drz-h8j	5HT2B	0.29	0.21	0.3	0.1	0.21	0.06	0.38	0.11	0.16	0.15	0.2	0.3	0.31	0.21	0.21	0.32	0.2	0.23	0.21	0.21	0.19	0.21	0.19	0.1	0.14	0.2	0.3	0.4
H8M	6ds0-h8m	5HT2B	0.18	0.18	0.19	0.5	0.21	0.47	0.45	0.36	0.26	0.31	0.25	0.19	0.19	0.09	0.09	0.35	0.27	0.28	0.48	0.41	0.33	0.41	0.31	-0.02	0.21	0.17	0.19	0.18
AZJ	4ntj-azj	P2Y12	-0.03	0.04	-0.18	-0.42	-0.1	-0.34	-0.28	-0.3	-0.13	-0.05	0.07	-0.18	-0.17	-0.15	-0.15	-0.13	-0.22	-0.19	-0.16	-0.11	-0.12	-0.11	-0.1	-0.29	-0.14	-0.12	-0.18	-0.13
6AD	4pxz-6ad	P2Y12	-0.37	-0.24	-0.54	-0.73	-0.26	-0.7	-0.79	-0.66	-0.27	-0.28	-0.17	-0.54	-0.6	-0.55	-0.55	-0.43	-0.55	-0.53	-0.49	-0.37	-0.42	-0.37	-0.37	-0.71	-0.28	-0.32	-0.54	-0.27
1Q5	4z9g-1q5	CRF1	0	0.05	-0.11	-0.42	-0.04	-0.47	-0.28	-0.3	-0.13	-0.04	-0.02	-0.11	-0.21	-0.08	-0.08	-0.03	-0.16	-0.07	-0.1	-0.02	-0.06	-0.02	0	-0.2	-0.03	-0.07	-0.11	-0.06
73R	5t1a-73r	CCR2	-0.48	-0.36	-0.33	-0.5	-0.22	-0.38	-0.62	-0.38	-0.43	-0.19	-0.28	-0.33	-0.5	-0.32	-0.32	-0.27	-0.25	-0.26	-0.41	-0.39	-0.38	-0.39	-0.13	-0.47	-0.25	-0.31	-0.33	-0.37
8ES	5unf-8es	AGTR2	0.15	0.17	-0.11	-0.12	0.07	-0.17	-0.07	0.13	-0.07	0.16	0.17	-0.11	-0.17	-0.08	-0.08	0.03	0.22	0.05	0.22	0.2	0.17	0.2	0.25	-0.2	0.09	0.08	-0.11	0.11
8EM	5unh-8em	AGTR2	0.02	0.04	-0.14	-0.17	0.03	-0.09	-0.41	-0.04	-0.04	0.12	0.12	-0.14	-0.08	-0.26	-0.26	-0.03	0.19	0	0.19	0.15	0.14	0.15	0.11	-0.41	0.06	0.05	-0.14	-0.02
AQD	5wiv-aqd	DRD4	0	0.07	-0.11	-0.21	0.14	-0.26	-0.45	-0.11	0.16	0.24	0.18	-0.11	-0.35	-0.08	-0.08	0.08	-0.05	0.05	0.19	0.17	0.04	0.17	0.24	-0.2	0.28	0.23	-0.11	-0.02
RET	6fuf-ret	GNAO	-0.05	0	-0.09	-0.29	-0.13	-0.21	0	-0.17	-0.07	-0.09	-0.07	-0.18	-0.12	-0.15	-0.15	-0.23	-0.22	-0.24	-0.22	-0.16	-0.2	-0.16	-0.13	-0.29	-0.17	-0.13	-0.18	-0.05

Target Ligand	Target Pdb	Protein	CAU	TIM	JRZ	JSZ	JTZ	CAU	QNB	OHK	OHK	2GM	ERM	ERM	ERM	ERM	7LD	H8G	H8D	H8J	H8M	AZJ	6AD	1Q5	73R	8ES	8EM	AQD	RET
	Ref. Pdb >		2rh1	3d4s	3ny8	3ny9	3nya	5d6l	3uon	5cxv	5dsg	4iaq	4iar	4ib4	4nc3	5tud	5tvn	6drx	6dry	6drz	6ds0	4ntj	4pxz	4z9g	5t1a	5unf	5unh	5wiv	6fuf
		Protein	ADRB2						ACM2		ACM4	5HT1B		5HT2B								P2Y12		CRF1	CCR2	AGTR2		DRD4	GNAO
ADN	2ydo-adn	AA2AR	0.31	0.31	0.33	0.22	0.24	0.31	0.24	0.25	0.24	0.21	0.25	0.07	0.1	0.29	0.26	0.28	0.01	0.23	0.15	-0.03	-0.18	0	-0.36	0.1	0.01	0.03	-0.07
NEC	2ydv-nec	AA2AR	0.34	0.35	0.32	0.26	0.22	0.34	0.17	0.22	0.17	0.27	0.3	0.12	0.17	0.26	0.31	0.33	0.07	0.2	0.19	0.04	-0.14	0.02	-0.33	0.15	0.03	0.06	0
ZMA	3eml-zma	AA2AR	0.1	0.1	0.19	0.04	0.11	0.1	0.12	0.13	0.12	0.04	0.09	0.08	0.12	0.31	0.08	0.09	0.11	0.22	0.15	-0.13	-0.24	-0.1	-0.29	-0.07	-0.08	-0.05	-0.24
ZMA	3pwh-zma	AA2AR	-0.06	-0.06	-0.14	-0.1	-0.18	-0.06	-0.04	-0.04	-0.04	-0.1	-0.05	-0.11	-0.08	0.04	-0.06	-0.06	-0.05	0.06	0.32	-0.25	-0.28	-0.36	-0.36	-0.07	-0.08	-0.09	-0.34
UKA	3qak-uka	AA2AR	0.16	0.2	0.19	0.19	0.14	0.16	0.2	0.21	0.2	0.23	0.26	0.2	0.23	0.38	0.24	0.36	0.28	-0.01	0.29	-0.14	-0.22	-0.07	-0.29	0.08	0.03	0.13	-0.34
XAC	3rey-xac	AA2AR	-0.09	-0.09	-0.11	-0.14	-0.15	-0.09	-0.09	-0.07	-0.09	-0.1	-0.05	-0.11	-0.08	0.04	-0.08	-0.09	-0.07	0.04	0.29	-0.2	-0.26	-0.4	-0.27	-0.09	-0.04	-0.1	-0.24
CFF	3rfm-cff	AA2AR	0.11	0.11	0.05	0.03	-0.01	0.11	0.11	0.14	0.11	-0.04	0.01	-0.06	-0.03	0.11	0.11	0.12	0.03	0.14	0.17	-0.1	-0.18	-0.16	-0.27	-0.02	-0.12	-0.11	0
T4G	3uza-t4g	AA2AR	0.03	0.03	-0.06	-0.04	-0.11	0.03	0.03	0.05	0.03	-0.01	0.04	-0.09	-0.06	0.06	0.01	0.01	-0.05	0.06	0.23	-0.18	-0.24	-0.26	-0.23	0.07	-0.02	-0.03	-0.2
T4E	3uzc-t4e	AA2AR	0.16	0.16	0.08	0.18	0.01	0.16	0.12	0.11	0.12	0.12	0.17	0.05	0.09	0.15	0.14	0.26	0.09	0.22	0.45	-0.11	-0.24	-0.03	-0.44	0.18	0.12	0.13	-0.32
NGL	4ug2-ngi	AA2AR	0.24	0.25	0.29	0.17	0.19	0.24	0.07	0.12	0.07	0.21	0.25	0.18	0.23	0.35	0.21	0.23	0.16	0.18	0.39	-0.06	-0.2	-0.12	-0.2	0.17	0.11	0.19	-0.2
NGI	4uhr-ngi	AA2AR	0.38	0.39	0.37	0.29	0.26	0.38	0.21	0.14	0.21	0.25	0.28	0.14	0.18	0.26	0.26	0.28	0.1	0.23	0.29	0.08	-0.12	-0.05	-0.3	0.17	0.11	0.16	-0.15
ZMA	5k2a-zma	AA2AR	0.1	0.1	0.19	0.04	0.11	0.1	0.12	0.13	0.12	0.04	0.09	0.08	0.12	0.31	0.08	0.09	0.11	0.22	0.15	-0.13	-0.24	-0.1	-0.29	-0.07	-0.08	-0.05	-0.24
ZMA	5k2b-zma	AA2AR	0.06	0.06	0.08	-0.01	0.01	0.06	0.06	0.09	0.06	0.02	0.07	-0.01	0.03	0.18	0.06	0.06	-0.03	0.19	0.12	-0.1	-0.23	-0.17	-0.36	-0.09	-0.04	-0.15	-0.15
ZMA	5k2c-zma	AA2AR	0.12	0.12	0.22	0.06	0.14	0.12	0.15	0.16	0.15	-0.02	0.03	0.03	0.07	0.24	0.11	0.12	0.04	0.14	0.07	-0.1	-0.23	-0.07	-0.26	-0.05	-0.14	-0.03	-0.2
ZMA	5k2d-zma	AA2AR	0.12	0.12	0.22	0.06	0.14	0.12	0.15	0.16	0.15	-0.02	0.03	0.03	0.07	0.24	0.11	0.12	0.04	0.14	0.07	-0.1	-0.23	-0.07	-0.26	-0.05	-0.14	-0.03	-0.2
ZMA	5olg-zma	AA2AR	0.15	0.12	0.16	0.11	0.08	0.15	0.2	0.13	0.2	0.12	0.17	0.21	0.26	0.29	0.18	0.27	0.21	0.32	0.36	-0.13	-0.26	-0.03	-0.32	0.02	-0.02	0.05	-0.44
9XT	5olh-9xt	AA2AR	0.04	0.04	-0.02	-0.01	-0.07	0.04	0.06	0.07	0.06	0	0.05	-0.02	0.01	0.15	0.04	0.04	-0.05	0.16	0.23	-0.18	-0.28	-0.17	-0.24	0.16	0.12	-0.03	-0.34
9XW	5olo-9xw	AA2AR	0.03	0.03	0.06	0.04	0	0.03	0.12	0.13	0.12	0.04	0.09	0.08	0.12	0.21	0.08	0.09	0.11	0.22	0.28	-0.18	-0.31	-0.09	-0.29	0.05	0	0.03	-0.44
92Y	5olv-92y	AA2AR	0.11	0.11	0.05	0.03	-0.01	0.11	0.04	0.07	0.04	0.06	0.11	0.02	0.06	0.23	0.11	0.12	-0.05	0.16	0.4	-0.13	-0.24	-0.1	-0.39	0.16	0.12	0.1	-0.34
T4E	5olz-t4e	AA2AR	0.16	0.16	0.08	0.18	0.01	0.16	0.12	0.11	0.12	0.12	0.17	0.05	0.09	0.15	0.14	0.26	0.09	0.22	0.45	-0.11	-0.24	-0.03	-0.44	0.18	0.12	0.03	-0.32
T4E	5om1-t4e	AA2AR	0.12	0.12	0.1	0.06	0.03	0.12	0.07	0.08	0.07	0.09	0.14	0.02	0.06	0.23	0.11	0.12	-0.05	0.16	0.31	-0.1	-0.23	-0.07	-0.35	0.14	0.1	0.03	-0.34
T4E	5om4-t4e	AA2AR	0.16	0.16	0.08	0.18	0.01	0.16	0.12	0.11	0.12	0.12	0.17	0.05	0.09	0.15	0.14	0.26	0.09	0.22	0.45	-0.11	-0.24	-0.03	-0.44	0.18	0.12	0.13	-0.32
DU1	5uen-du1	AA2AR	0.21	0.21	0.3	0.2	0.21	0.21	0.21	0.22	0.21	0.09	0.14	0.12	0.17	0.27	0.26	0.28	0.1	0.2	0.35	-0.1	-0.24	0	-0.15	0.24	0.09	0.17	-0.27
ZMA	5uvi-zma	AA2AR	0.05	0.05	0.13	0	0.06	0.05	0.07	0.08	0.07	-0.08	-0.03	-0.02	0.02	0.17	0.04	0.04	-0.04	0.06	-0.01	-0.18	-0.28	-0.17	-0.35	-0.11	-0.21	-0.09	-0.34
UKA	5wf5-uka	AA2AR	0.19	0.23	0.22	0.21	0.17	0.19	0.12	0.13	0.12	0.23	0.28	0.2	0.23	0.38	0.26	0.28	0.2	0.22	0.33	-0.22	-0.27	-0.07	-0.38	0.12	0.07	0.29	-0.49
UKA	5wf6-uka	AA2AR	0.2	0.2	0.22	0.09	0.14	0.2	0.19	0.21	0.19	0.31	0.34	0.27	0.3	0.38	0.33	0.38	0.3	0.27	0.2	-0.16	-0.27	-0.12	-0.38	0.1	0.05	0.22	-0.34
ZMA	6aqf-zma	AA2AR	0.1	0.1	0.19	0.04	0.11	0.1	0.12	0.13	0.12	0.04	0.09	0.08	0.12	0.31	0.08	0.09	0.11	0.22	0.15	-0.13	-0.24	-0.1	-0.29	-0.07	-0.08	-0.05	-0.24
NEC	6gdg-nec	AA2AR	0.2	0.31	0.13	0.2	0.21	0.2	0.3	0.34	0.3	0.22	0.25	0.07	0.11	0.3	0.25	0.27	0.1	0.32	0.15	-0.1	-0.13	-0.07	-0.27	0.08	-0.01	0.02	-0.07
CAU	2rh1-cau	ADRB2	1	0.89	0.87	0.79	0.82	1	0.51	0.46	0.51	0.44	0.47	0.28	0.26	0.35	0.58	0.5	0.21	0.38	0.35	0.27	-0.05	0.19	-0.42	0.03	-0.09	0.43	-0.1
TIM	3d4s-tim	ADRB2	0.89	1	0.87	0.79	0.94	0.89	0.49	0.43	0.49	0.46	0.49	0.26	0.24	0.35	0.61	0.53	0.21	0.35	0.35	0.15	-0.03	0.16	-0.3	0.12	-0.01	0.37	0.24
JRZ	3ny8-jrz	ADRB2	0.69	0.69	1	0.52	0.82	0.69	0.49	0.43	0.49	0.22	0.25	0.2	0.17	0.27	0.3	0.22	0.21	0.23	0.11	0.13	-0.06	0.16	-0.42	-0.03	-0.09	0.23	0.1
JSZ	3ny9-jsz	ADRB2	0.93	0.93	0.78	1	0.86	0.93	0.53	0.47	0.53	0.49	0.52	0.3	0.29	0.27	0.54	0.55	0.24	0.38	0.37	0.19	0.01	0.29	-0.39	0.06	-0.07	0.47	0.1
JTZ	3nya-jtz	ADRB2	0.74	0.85	0.94	0.66	1	0.74	0.44	0.38	0.44	0.34	0.37	0.23	0.21	0.32	0.44	0.37	0.16	0.18	0.16	0.18	0	0.22	-0.36	0.01	-0.05	0.34	0.34
CAU	5d6l-cau	ADRB2	1	0.89	0.87	0.79	0.82	0.2	0.51	0.46	0.51	0.44	0.47	0.28	0.26	0.35	0.58	0.5	0.21	0.32	0.35	0.27	-0.05	0.19	-0.42	0.03	-0.09	0.43	-0.1
QNB	3uon-qnb	ACM2	0.51	0.49	0.62	0.45	0.49	0.51	1	0.97	1	0.43	0.46	0.33	0.39	0.36	0.48	0.58	0.39	0.62	0.25	0.1	-0.01	0.02	-0.36	-0.01	-0.13	0.25	-0.05
OHK	5cxv-0hk	ACM2	0.41	0.41	0.52	0.38	0.4	0.41	0.93	1	0.93	0.45	0.48	0.34	0.4	0.38	0.49	0.5	0.31	0.54	0.25	0.03	-0.01	-0.09	-0.33	0.01	-0.11	0.27	-0.17
OHK	5dsg-0hk	ACM4	0.51	0.49	0.62	0.45	0.49	0.51	1	0.97	1	0.43	0.46	0.33	0.39	0.36	0.48	0.58	0.39	0.62	0.25	0.1	-0.01	0.02	-0.36	-0.01	-0.13	0.25	-0.05
2GM	4iaq-2gm	5HT1B	0.54	0.56	0.43	0.51	0.46	0.54	0.53	0.58	0.53	1	0.93	0.5	0.5	0.62	0.85	0.63	0.49	0.51	0.32	-0.01	-0.16	0.26	-0.27	-0.01	-0.05	0.41	-0.2
ERM	4iar-erm	5HT1B	0.6	0.62	0.51	0.56	0.53	0.6	0.59	0.64	0.59	0.98	1	0.56	0.57	0.61	0.9	0.69	0.46	0.57	0.39	-0.04	-0.12	0.22	-0.3	-0.03	-0.07	0.45	-0.24
ERM	4ib4-erm	5HT2B	0.42	0.4	0.38	0.38	0.39	0.42	0.51	0.55	0.51	0.64	0.68	1	0.97	0.81	0.71	0.58	0.66	0.77	0.41	-0.19	-0.18	0.26	-0.42	0	-0.04	0.36	-0.44
ERM	4nc3-erm	5HT2B	0.36	0.34	0.3	0.33	0.32	0.36	0.56	0.61	0.56	0.6	0.64	0.9	1	0.76	0.65	0.63	0.71	0.82	0.47	-0.14	-0.15	0.05	-0.36	0.05	0	0.32	-0.34
ERM	5tud-erm	5HT2B	0.38	0.38	0.38	0.26	0.39	0.38	0.38	0.42	0.38	0.54	0.5	0.55	0.56	1	0.62	0.47	0.56	0.67	0.49	-0.13	-0.24	0.1	-0.18	0.03	0.01	0.22	-0.22
7LD	5tvn-7ld	5HT2B	0.61	0.64	0.49	0.46	0.51	0.61	0.5	0.54	0.5	0.73	0.75	0.49	0.49	0.61	1	0.79	0.56	0.67	0.49	0.06	-0.13	0.26	-0.32	0.06	-0.07	0.36	-0.05
H8G	6drx-h8g	5HT2B	0.49	0.51	0.37	0.46	0.4	0.49	0.56	0.51	0.56	0.51	0.53	0.37	0.43	0.44	0.74	1	0.59	0.7	0.43	0.06	-0.13	0.12	-0.53	0.06	-0.07	0.29	-0.05
H8D	6dry-h8d	5HT2B	0.21	0.21	0.27	0.2	0.18	0.21	0.39	0.33	0.39	0.4	0.36	0.43	0.5	0.54	0.54	0.6	1	0.78	0.52	-0.19	-0.23	-0.26	-0.15	-0.1	-0.13	0.1	0
H8J	6drz-h8j	5HT2B	0.38	0.35	0.29	0.32	0.19	0.38	0.61	0.57	0.61	0.41	0.44	0.5	0.57	0.63	0.63	0.71	0.78	1	0.55	-0.15	-0.21	-0.1	-0.29	-0.01	-0.05	0.13	-0.17
H8M	6ds0-h8m	5HT2B	0.33	0.33	0.13	0.3	0.17	0.33	0.24	0.25	0.24	0.26	0.29	0.24	0.3	0.44	0.45	0.41	0.49	0.52	1	-0.11	-0.19	-0.16	-0.18	0.02	-0.02	0.16	0.12
AZJ	4ntj-azj	P2Y12	0.26	0.15	0.16	0.16	0.19	0.26	0.1	0.03	0.1	-0.01	-0.03	-0.12	-0.1	-0.12	0.06	0.06	-0.19	-0.15	-0.12	1	0.34	0.16	-0.5	0.12	0.12	0.04	0.1
6AD	4pxz-6ad	P2Y12	-0.11	-0.05	-0.11	0.01	0	-0.11	-0.09	-0.01	-0.09	-0.21	-0.15	-0.24	-0.22	-0.37	-0.19	-0.21	-0.36	-0.34	-0.32	0.51	1	-0.24	-0.7	-0.19	-0.14	-0.01	-0.24
1Q5	4z9g-1q5	CRF1	0.1	0.1	0.14	0.18	0.18	0.1	0.01	-0.07	0.01	0.15	0.13	0.12	0.03	0.07	0.18	0.08	-0.2	-0.08	-0.11	0.11	-0.07	1	-0.73	0	-0.12	0.01	-0.12
73R	5t1a-73r	CCR2	-0.36	-0.25	-0.43	-0.28	-0.33	-0.36	-0.31	-0.3	-0.31	-0.22	-0.23	-0.25	-0.23	-0.18	-0.29	-0.49	-0.14	-0.25	-0.16	-0.42	-0.36	-0.83	1	-0.09	-0.12	-0.37	-0.78
8ES	5unf-8es	AGTR2	0.05	0.14	-0.05	0.05	0.01	0.05	-0.01	0.01	-0.01	0	-0.02	0	0.03	0.04	0.07	0.08	-0.11	-0.01	0.03	0.14	-0.13	0	-0.12	1	0.78	0.14	-0.32
8EM	5unh-8em	AGTR2	-0.1	-0.01	-0.14	-0.07	-0.07	-0.1	-0.16	-0.14	-0.16	-0.04	-0.06	-0.03	0	0.01	-0.07	-0.08	-0.16	-0.06	-0.03	0.15	-0.09	-0.21	-0.18	0.86	1	0.08	-0.41
AQD	5wiv-aqd	DRD4	0.62	0.54	0.52	0.59	0.54	0.62	0.35	0.41	0.35	0.48	0.51	0.31	0.3	0.29	0.5	0.44	0.15	0.19	0.25	0.06	-0.01	0.02	-0.61	0.17	0.03	1	-0.39
RET	6fuf-ret	GNAO	0.01	0.12	0.06	0.04	0.19	0.01	-0.03	-0.11	-0.03	-0.03	-0.05	-0.12	-0.1	-0.07	0.04	0.04	0	-0.09	0.07	0.05	-0.07	-0.09	-0.44	-0.15	-0.18	-0.09	1

Figure S10: Matrix of the normalized scores for the 8 digit profiling scheme using GPCRtm Scoring with a miss and gap penalty of -2. Each element has been coloured following a RdYlGn palette, where higher scores are green in colour, while the lower scores are red. The elements coloured in azure are all values that are >0.5 as these are scores that are of interest.

GPCRtm score with -2.0 miss - 10 digit profile

		Ref. Pdb >	2ydo	2ydv	3eml	3pwh	3qak	3rey	3rfm	3uza	3uzc	4ug2	4uhr	5k2a	5k2b	5k2c	5k2d	5olg	5olh	5olo	5olv	5olz	5om1	5om4	5uen	5uvi	5wf5	5wf6	6aqf	6gdg	2rh1	3d4s	3ny8	3ny9	3nya	5d6l	3uon	5cxv	5dsg	4iaq	4iar	4ib4	4nc3	5tud	5tvn	6drx	6dry	6drz	6ds0	4ntj	4pxz	4z9g	5t1a	5unf	5unh	5wiv	6fuf
		Ref. Ligand >	ADN	NEC	ZMA	ZMA	UKA	XAC	CFF	T4G	T4E	NGL	NGI	ZMA	ZMA	ZMA	ZMA	ZMA	9XT	9XW	92Y	T4E	T4E	T4E	DU1	ZMA	UKA	UKA	ZMA	NEC	CAU	TIM	JRZ	JSZ	JTZ	CAU	QNB	OHK	OHK	2GM	ERM	ERM	ERM	ERM	7LD	H8G	H8D	H8J	H8M	AZJ	6AD	1Q5	73R	8ES	8EM	AQD	RET
Target Ligand v	Target Pdb v	Protein	AA2AR																												ADRB2						ACM2		ACM4	5HT1B		5HT2B								P2Y12		CRF1	CCR2	AGTR2		DRD4	GNAO
ADN	2ydo-adn	AA2AR	1	0.74	0.56	0.36	0.4	0.1	0.6	0.4	0.47	0.52	0.57	0.56	0.58	0.6	0.6	0.41	0.4	0.21	0.35	0.43	0.59	0.43	0.38	0.53	0.31	0.4	0.56	0.62	0.12	0.12	0.25	0.11	0.17	0.12	0.06	0	-0.07	0.07	0.07	0.03	0.07	0.13	0.2	0.14	0.04	0.17	0.06	-0.18	-0.22	-0.16	-0.35	-0.1	-0.06	-0.05	-0.1
NEC	2ydv-nec		0.91	1	0.61	0.24	0.58	0.17	0.4	0.46	0.6	0.73	0.79	0.61	0.64	0.65	0.65	0.78	0.44	0.28	0.39	0.57	0.63	0.57	0.33	0.59	0.47	0.45	0.61	0.8	0.14	0.14	0.24	0.14	0.15	0.14	0	-0.08	-0.01	0.12	0.14	0.03	0.07	0.13	0.26	0.2	0.1	0.14	0.1	-0.12	-0.14	-0.13	-0.41	-0.06	-0.02	-0.02	0.02
ZMA	3eml-zma		0.52	0.46	1	0.4	0.34	0.42	0.67	0.48	0.49	0.45	0.4	1	0.92	0.96	0.96	0.61	0.56	0.56	0.45	0.45	0.62	0.45	0.27	0.92	0.25	0.34	1	0.41	-0.07	-0.07	0.03	-0.06	-0.03	-0.07	-0.07	-0.13	-0.2	-0.17	-0.13	0	0.03	0.08	0.03	-0.04	0.06	0.08	0.09	-0.2	-0.23	-0.26	-0.36	-0.26	-0.2	-0.17	-0.26
ZMA	3pwh-zma		0.28	0.15	0.34	1	0.14	0.52	0.53	0.38	0.24	0.22	0.15	0.34	0.32	0.4	0.4	0.34	0.51	0.29	0.39	0.21	0.34	0.21	0.34	0.47	0.15	0.15	0.34	0.03	-0.22	-0.22	-0.16	-0.19	-0.19	-0.22	-0.15	-0.22	-0.28	-0.22	-0.2	-0.19	-0.17	-0.2	-0.09	-0.11	-0.18	-0.07	0.16	-0.36	-0.31	-0.36	-0.41	-0.26	-0.23	-0.14	-0.52
UKA	3qak-uka		0.69	0.82	0.63	0.4	1	0.31	-0.07	0.33	0.63	0.84	0.81	0.63	0.48	0.56	0.56	0.78	0.49	0.52	0.5	0.6	0.66	0.6	0.43	0.49	0.85	0.85	0.63	0.72	0.06	0.06	0.13	0.07	0.06	0.06	0	0.02	-0.01	0.19	0.21	0.16	0.19	0.32	0.18	0.3	0.3	0.24	0.22	-0.29	-0.21	-0.2	-0.45	-0.03	-0.01	0.09	-0.31
XAC	3rey-xac		0.08	0.1	0.34	0.5	0.14	1	0.43	0.54	0.23	0.22	0.15	0.34	0.36	0.25	0.25	0.34	0.4	0.29	0.61	0.21	0.34	0.21	0.15	0.16	0.15	0.15	0.34	0.19	-0.27	-0.27	-0.24	-0.24	-0.26	-0.27	-0.27	-0.27	-0.28	-0.18	-0.15	-0.14	-0.12	-0.02	-0.15	-0.14	-0.13	-0.02	0.22	-0.26	-0.3	-0.38	-0.43	-0.2	-0.08	-0.12	-0.38
CFF	3rfm-cff		0.28	0.15	0.34	0.32	-0.02	0.27	1	0.46	0.15	0.04	0.07	0.34	0.36	0.4	0.4	0.12	0.24	0.16	0.21	0.1	0.2	0.1	0.02	0.31	-0.07	-0.02	0.34	0.25	-0.16	-0.16	-0.09	-0.14	-0.13	-0.16	-0.16	-0.23	-0.26	-0.17	-0.16	-0.09	-0.07	-0.06	0.04	-0.02	-0.04	0.07	0.1	-0.19	-0.22	-0.13	-0.35	-0.23	-0.19	-0.24	-0.19
T4G	3uza-t4g		0.52	0.33	0.39	0.36	0.15	0.54	0.73	1	0.43	0.25	0.26	0.39	0.42	0.42	0.42	0.26	0.49	0.41	0.5	0.4	0.56	0.4	0.06	0.33	0.15	0.22	0.39	0.41	-0.17	-0.17	-0.13	-0.15	-0.17	-0.17	-0.17	-0.24	-0.28	-0.17	-0.13	-0.12	-0.1	-0.1	-0.04	-0.12	-0.11	0	0.15	-0.26	-0.28	-0.23	-0.39	-0.27	-0.15	-0.13	-0.38
T4E	3uzc-t4e		0.56	0.62	0.64	0.36	0.45	0.38	0.27	0.71	1	0.6	0.64	0.64	0.68	0.58	0.58	0.65	0.62	0.6	0.67	1	0.92	1	0.34	0.51	0.44	0.45	0.64	0.45	-0.03	-0.03	0.01	-0.01	-0.04	-0.03	-0.07	-0.13	-0.21	0.05	0.07	-0.02	0.01	0.02	0.09	0.01	-0.07	0.06	0.18	-0.21	-0.24	-0.33	-0.57	-0.13	-0.1	0.07	-0.29
NGL	4ug2-ngl		0.78	0.9	0.73	0.42	0.74	0.44	0.13	0.5	0.71	1	0.88	0.73	0.6	0.67	0.67	0.64	0.47	0.51	0.59	0.69	0.77	0.69	0.5	0.61	0.68	0.63	0.73	0.67	0.02	0.02	0.12	0.04	0.05	0.02	-0.05	-0.2	-0.13	0.06	0.08	0.04	0.08	0.14	0.17	0.11	0.11	0.05	0.22	-0.21	-0.2	-0.26	-0.36	-0.1	0	0.06	-0.17
NGI	4uhr-ngi		0.81	0.92	0.61	0.28	0.67	0.29	0.2	0.5	0.71	0.83	1	0.61	0.64	0.55	0.55	0.55	0.47	0.39	0.48	0.69	0.77	0.69	0.35	0.47	0.55	0.56	0.61	0.7	0.17	0.17	0.28	0.17	0.19	0.17	0.03	-0.05	0.02	0.1	0.12	0	0.03	0.06	0.21	0.16	0.05	0.07	0.13	-0.08	-0.11	-0.2	-0.46	-0.08	0	0.07	-0.12
ZMA	5k2a-zma		0.52	0.46	1	0.4	0.34	0.42	0.67	0.48	0.49	0.45	0.4	1	0.92	0.96	0.96	0.61	0.56	0.56	0.45	0.45	0.62	0.45	0.27	0.92	-0.06	0.34	1	0.41	-0.07	-0.07	0.03	-0.06	-0.03	-0.07	-0.07	-0.13	-0.2	-0.17	-0.13	0	0.03	0.08	0.03	-0.04	0.06	0.08	0.09	-0.2	-0.23	-0.26	-0.36	-0.26	-0.2	-0.17	-0.26
ZMA	5k2b-zma		0.45	0.41	0.78	0.32	0.22	0.38	0.6	0.44	0.45	0.31	0.35	0.78	1	0.73	0.73	0.45	0.5	0.39	0.42	0.4	0.56	0.4	0.12	0.67	0.15	0.22	0.78	0.38	-0.1	-0.1	-0.04	-0.15	-0.09	-0.1	-0.17	-0.18	-0.2	-0.18	-0.15	-0.09	-0.06	-0.02	0.01	-0.06	-0.07	0.06	0.06	-0.2	-0.26	-0.3	-0.43	-0.26	-0.2	-0.26	-0.26
ZMA	5k2c-zma		0.52	0.46	0.9	0.44	0.28	0.29	0.73	0.48	0.42	0.39	0.33	0.9	0.8	1	1	0.54	0.59	0.48	0.36	0.38	0.54	0.38	0.3	0.96	0.2	0.28	0.9	0.41	-0.05	-0.05	0.06	-0.04	0	-0.05	-0.05	-0.11	-0.17	-0.17	-0.13	0	0.03	0.08	0.06	-0.01	0.06	0.08	0.09	-0.18	-0.21	-0.23	-0.33	-0.24	-0.2	-0.16	-0.21
ZMA	5k2d-zma		0.52	0.46	0.9	0.44	0.28	0.29	0.73	0.48	0.42	0.39	0.33	0.9	0.8	1	1	0.54	0.59	0.48	0.36	0.38	0.54	0.38	0.3	0.96	0.2	0.28	0.9	0.41	-0.05	-0.05	0.06	-0.04	0	-0.05	-0.05	-0.11	-0.17	-0.17	-0.13	0	0.03	0.08	0.06	-0.01	0.06	0.08	0.09	-0.18	-0.21	-0.23	-0.33	-0.24	-0.2	-0.16	-0.21
ZMA	5olg-zma		0.52	0.49	0.83	0.54	0.56	0.56	0.33	0.44	0.65	0.53	0.48	0.83	0.72	0.78	0.78	1	0.66	0.64	0.68	0.62	0.7	0.62	0.53	0.73	0.46	0.55	0.83	0.41	0.01	0.01	0.1	0.01	0.04	0.01	0.01	-0.07	0.02	0.02	0.05	0.16	0.21	0.23	0.1	0.22	0.24	0.27	0.28	-0.23	-0.29	-0.16	-0.45	-0.08	-0.06	-0.02	-0.4
9XT	5olh-9xt		0.42	0.38	0.64	0.74	0.3	0.56	0.53	0.69	0.54	0.33	0.35	0.64	0.68	0.73	0.73	0.56	1	0.49	0.65	0.5	0.68	0.5	0.47	0.67	0.28	0.37	0.64	0.31	-0.11	-0.11	-0.03	-0.1	-0.08	-0.11	-0.11	-0.18	-0.24	-0.14	-0.1	-0.1	-0.08	-0.07	0.01	0	-0.1	0.04	0.16	-0.25	-0.26	-0.33	-0.36	-0.09	-0.08	-0.07	-0.36
9XW	5olo-9xw		0.25	0.27	0.71	0.44	0.36	0.46	0.4	0.65	0.54	0.4	0.32	0.71	0.58	0.65	0.65	0.6	0.54	1	0.44	0.54	0.61	0.54	0.28	0.59	0.4	0.41	0.71	0.14	-0.24	-0.24	-0.22	-0.21	-0.24	-0.24	-0.17	-0.24	-0.3	-0.25	-0.16	-0.01	0.01	0.07	-0.07	-0.14	-0.02	-0.02	0.05	-0.36	-0.33	-0.23	-0.45	-0.29	-0.23	-0.11	-0.57
92Y	5olv-92y		0.36	0.33	0.51	0.52	0.3	0.83	0.47	0.69	0.59	0.41	0.35	0.51	0.56	0.44	0.44	0.56	0.63	0.39	1	0.52	0.68	0.52	0.35	0.35	0.36	0.37	0.51	0.47	-0.07	-0.07	0.03	-0.06	-0.03	-0.07	-0.14	-0.2	-0.24	-0.08	-0.05	0	0.03	0.08	0.06	0.07	-0.01	0.12	0.32	-0.25	-0.27	-0.23	-0.52	-0.1	0	0.03	-0.36
T4E	5olz-t4e		0.56	0.62	0.64	0.36	0.45	0.38	0.27	0.71	1	0.6	0.64	0.64	0.68	0.58	0.58	0.65	0.62	0.6	0.67	1	0.92	1	0.34	0.51	0.44	0.45	0.64	0.45	-0.03	-0.03	0.01	-0.01	-0.04	-0.03	-0.07	-0.13	-0.21	0.05	0.07	-0.02	0.01	0.02	0.09	0.01	-0.07	0.06	0.18	-0.21	-0.24	-0.33	-0.57	-0.13	-0.1	0.07	-0.29
T4E	5om1-t4e		0.66	0.58	0.75	0.48	0.43	0.5	0.47	0.83	0.79	0.57	0.6	0.75	0.8	0.69	0.69	0.62	0.71	0.57	0.73	0.77	1	0.77	0.32	0.63	0.4	0.5	0.75	0.55	-0.05	-0.05	0.03	-0.04	-0.03	-0.05	-0.11	-0.18	-0.24	-0.06	-0.03	-0.05	-0.03	0	0.06	-0.01	-0.1	0.04	0.14	-0.25	-0.26	-0.23	-0.48	-0.2	-0.08	-0.04	-0.36
T4E	5om4-t4e		0.56	0.62	0.64	0.36	0.45	0.38	0.27	0.75	1	0.6	0.64	0.64	0.68	0.58	0.58	0.65	0.62	0.6	0.67	1	0.92	1	0.34	0.51	0.44	0.45	0.64	0.45	-0.03	-0.03	0.01	-0.01	-0.04	-0.03	-0.07	-0.13	-0.21	0.05	0.07	-0.02	0.01	0.02	0.09	0.01	-0.07	0.06	0.18	-0.21	-0.24	-0.33	-0.57	-0.13	-0.1	0.07	-0.29
DU1	5uen-du1		0.53	0.33	0.41	0.6	0.35	0.27	0.07	0.08	0.4	0.46	0.31	0.41	0.22	0.47	0.47	0.59	0.6	0.33	0.47	0.35	0.38	0.35	1	0.39	0.33	0.35	0.41	0.09	0.11	0.11	0.24	0.1	0.15	0.11	0.11	0.06	-0.01	0.04	0.05	0.02	0.06	0.11	0.11	0.12	0.02	0.05	0.08	-0.27	-0.27	-0.3	-0.36	0.01	-0.03	0.15	-0.24
ZMA	5uvi-zma		0.42	0.38	0.8	0.48	0.23	0.17	0.53	0.35	0.36	0.32	0.26	0.8	0.68	0.89	0.89	0.89	0.5	0.4	0.27	0.31	0.45	0.31	0.23	1	0.15	0.23	0.8	0.31	-0.11	-0.11	-0.03	-0.1	-0.08	-0.11	-0.05	-0.11	-0.17	-0.22	-0.19	-0.05	-0.02	0.01	-0.01	-0.08	-0.01	0.01	0.01	-0.25	-0.26	-0.33	-0.33	-0.3	-0.25	-0.2	-0.36
UKA	5wf5-uka		0.59	0.74	0.53	0.36	0.95	0.38	-0.27	0.38	0.68	0.88	0.75	0.53	0.36	0.45	0.45	0.7	0.51	0.65	0.67	0.64	0.69	0.64	0.47	0.37	1	0.87	0.53	0.62	-0.01	-0.01	0.04	0.01	-0.01	-0.01	-0.07	-0.05	-0.08	0.14	0.21	0.16	0.19	0.32	0.11	0.23	0.23	0.17	0.25	-0.36	-0.25	-0.16	-0.54	-0.05	-0.03	0.18	-0.45
UKA	5wf6-uka		0.69	0.63	0.63	0.34	0.85	0.35	-0.07	0.5	0.62	0.71	0.65	0.63	0.48	0.56	0.56	0.75	0.6	0.6	0.62	0.58	0.77	0.58	0.43	0.49	0.77	1	0.63	0.59	0.08	0.08	0.16	0.05	0.09	0.08	0.09	0.01	0.08	0.21	0.23	0.18	0.21	0.32	0.2	0.33	0.33	0.22	0.13	-0.26	-0.25	-0.3	-0.45	-0.09	-0.05	0.09	-0.31
ZMA	6aqf-zma		0.52	0.46	1	0.4	0.34	0.42	0.67	0.48	0.49	0.45	0.4	1	0.92	0.96	0.96	0.61	0.56	0.56	0.45	0.45	0.62	0.45	0.27	0.92	0.25	0.34	1	0.41	-0.07	-0.07	0.03	-0.06	-0.03	-0.07	-0.07	-0.13	-0.2	-0.17	-0.13	0	0.03	0.08	0.03	-0.04	0.06	0.08	0.09	-0.2	-0.23	-0.26	-0.36	-0.26	-0.2	-0.17	-0.26
NEC	6gdg-nec		0.61	0.65	0.54	0.04	0.42	0.25	0.53	0.54	0.42	0.45	0.49	0.44	0.48	0.47	0.47	0.33	0.29	0.12	0.45	0.35	0.49	0.35	0.09	0.39	0.33	0.35	0.44	1	-0.02	0.03	0.06	0.03	0.06	-0.02	0.09	0.04	-0.01	0.06	0.08	0.04	0.08	0.14	0.19	0.13	0.12	0.25	0.04	-0.18	-0.12	-0.2	-0.35	-0.12	-0.08	-0.07	-0.1
CAU	2rh1-cau	ADRB2	0.17	0.15	-0.1	-0.38	0.03	-0.5	-0.47	-0.31	0.06	0.02	0.16	-0.1	-0.18	-0.07	-0.07	0.01	-0.15	-0.28	-0.09	-0.04	-0.06	-0.04	0.11	-0.2	-0.02	0.05	-0.1	-0.02	1	0.9	0.88	0.8	0.83	1	0.45	0.4	0.38	0.41	0.44	0.18	0.15	0.16	0.4	0.36	0.17	0.17	0.18	-0.04	-0.15	-0.18	-0.61	-0.14	-0.19	0.2	-0.21
TIM	3d4s-tim		0.17	0.15	-0.1	-0.38	0.03	-0.5	-0.47	-0.31	0.06	0.02	0.16	-0.1	-0.18	-0.07	-0.07	0.01	-0.15	-0.28	-0.09	-0.04	-0.06	-0.04	0.11	-0.2	-0.02	0.05	-0.1	-0.02	0.9	1	0.88	0.8	0.95	0.9	0.45	0.4	0.38	0.41	0.44	0.16	0.13	0.16	0.43	0.39	0.17	0.17	0.18	-0.14	-0.15	-0.21	-0.58	-0.14	-0.19	0.12	-0.21
JRZ	3ny8-jrz		0.27	0.21	0.03	-0.05	0.08	-0.33	-0.2	-0.19	0.1	0.08	0.21	0.03	-0.06	0.07	0.07	0.09	-0.03	-0.2	0.03	0.01	0.03	0.01	0.18	-0.04	0.02	0.1	0.03	0.08	0.68	0.68	1	0.53	0.82	0.68	0.42	0.36	0.34	0.29	0.32	0.21	0.18	0.23	0.35	0.35	0.24	0.29	0.16	-0.05	-0.16	-0.2	-0.49	-0.04	-0.09	0.19	-0.02
JSZ	3ny9-jsz		0.17	0.18	-0.1	-0.18	0.05	-0.5	-0.47	-0.31	0.09	0.04	0.19	-0.1	-0.3	-0.07	-0.07	0.01	-0.15	-0.28	-0.09	-0.01	-0.06	-0.01	0.11	-0.2	-0.01	0.03	-0.1	-0.02	0.93	0.93	0.79	1	0.87	0.93	0.43	0.37	0.4	0.45	0.47	0.21	0.18	0.16	0.43	0.39	0.17	0.17	0.18	-0.04	-0.05	-0.07	-0.67	-0.14	-0.19	0.3	-0.21
JTZ	3nya-jtz		0.2	0.15	-0.03	-0.3	0.05	-0.42	-0.33	-0.27	0.06	0.04	0.16	-0.03	-0.14	0	0	0.04	-0.09	-0.25	-0.03	-0.04	-0.03	-0.04	0.14	-0.12	-0.01	0.06	-0.03	0.02	0.74	0.84	0.94	0.67	1	0.74	0.4	0.34	0.32	0.36	0.39	0.2	0.17	0.21	0.43	0.43	0.19	0.24	0.23	-0.1	-0.12	-0.15	-0.52	-0.08	-0.13	-0.07	-0.12
CAU	5d6l-cau		0.17	0.15	-0.1	-0.38	0.03	-0.5	-0.47	-0.31	0.06	0.02	0.16	-0.1	-0.18	-0.07	-0.07	0.01	-0.15	-0.28	-0.09	-0.04	-0.06	-0.04	0.11	-0.2	-0.02	0.05	-0.1	-0.02	1	0.9	0.88	0.8	0.83	1	0.45	0.4	0.38	0.41	0.44	0.18	0.15	0.16	0.4	0.36	0.17	0.17	0.18	-0.04	-0.15	-0.18	-0.61	-0.14	-0.19	0.2	-0.21
QNB	3uon-qnb	ACM2	0.08	0.04	0.04	-0.26	-0.03	-0.5	-0.47	-0.31	0.03	-0.07	0.03	-0.1	-0.3	-0.07	-0.07	0.01	-0.15	-0.2	-0.18	-0.07	-0.14	-0.14	0.11	-0.08	-0.07	0.05	-0.1	0.12	0.38	0.45	0.54	0.33	0.45	0.38	1	0.98	0.93	0.26	0.3	0.23	0.28	0.31	0.2	0.24	0.24	0.35	-0.09	-0.06	-0.05	-0.34	-0.55	-0.1	-0.21	0.21	-0.33
OHK	5cxv-0hk		-0.05	-0.09	-0.24	-0.42	-0.01	-0.46	-0.67	-0.44	-0.06	-0.18	-0.04	-0.24	-0.3	-0.22	-0.22	-0.09	-0.26	-0.27	-0.27	-0.13	-0.25	-0.13	0.06	-0.24	-0.06	-0.01	-0.24	0.03	0.3	0.38	0.44	0.26	0.36	0.3	0.92	1	0.93	0.28	0.31	0.24	0.29	0.33	0.12	0.27	0.27	0.37	-0.06	-0.06	-0.05	-0.31	-0.52	-0.08	-0.19	0.23	-0.29
OHK	5dsg-0hk	ACM4	-0.08	-0.01	-0.27	-0.46	-0.03	-0.5	-0.73	-0.48	-0.13	-0.11	0.02	-0.27	-0.34	-0.25	-0.25	0.03	-0.29	-0.35	-0.3	-0.2	-0.28	-0.2	-0.01	-0.27	-0.07	0.05	-0.27	0	0.3	0.38	0.44	0.34	0.36	0.3	0.92	0.98	1	0.26	0.3	0.23	0.28	0.31	0.19	0.24	0.24	0.35	-0.09	0.01	-0.05	-0.23	-0.55	-0.1	-0.21	0.21	-0.17
2GM	4iaq-2gm	5HT1B	0.11	0.15	-0.31	-0.46	0.18	-0.4	-0.6	-0.35	0.13	0.06	0.11	-0.31	-0.38	-0.29	-0.29	0.03	-0.21	-0.36	-0.12	0.06	-0.08	0.06	0.05	-0.43	0.11	0.2	-0.31	0.09	0.48	0.48	0.44	0.45	0.47	0.48	0.38	0.42	0.37	1	0.94	0.53	0.55	0.59	0.71	0.64	0.52	0.57	0.38	-0.13	-0.19	-0.1	-0.35	0.02	-0.09	0.35	-0.17
ERM	4iar-erm		0.08	0.13	-0.34	-0.5	0.16	-0.44	-0.67	-0.4	0.1	0.04	0.09	-0.34	-0.42	-0.33	-0.33	0	-0.24	-0.24	-0.15	0.04	-0.11	0.04	0.02	-0.47	0.19	0.18	-0.34	0.06	0.53	0.53	0.51	0.5	0.54	0.53	0.43	0.48	0.43	0.98	1	0.59	0.59	0.64	0.76	0.7	0.49	0	0.44	-0.15	-0.15	-0.02	-0.38	0	-0.11	0.39	-0.21
ERM	4ib4-erm	5HT2B	0.05	0.01	-0.05	-0.52	0.16	-0.44	-0.43	-0.4	0.06	0.02	-0.03	-0.05	-0.28	-0.05	-0.05	0.23	-0.25	-0.01	-0.05	-0.07	-0.14	-0.07	0.03	-0.18	0.19	0.15	-0.05	0.03	0.26	0.24	0.4	0.26	0.32	0.26	0.35	0.4	0.37	0.66	0.69	1	0.97	0.82	0.64	0.59	0.67	0.78	0.35	-0.23	-0.15	-0.02	-0.41	0.03	-0.01	0.36	-0.4
ERM	4nc3-erm		0.08	0.04	-0.02	-0.48	0.18	-0.4	-0.37	-0.35	0.08	0.04	-0.01	-0.02	-0.24	-0.02	-0.02	0.25	-0.22	0.01	-0.02	-0.05	-0.11	-0.11	0.06	-0.14	0.2	0.16	-0.02	0.06	0.2	0.18	0.32	0.22	0.26	0.2	0.4	0.45	0.41	0.63	0.65	0.9	1	0.77	0.58	0.64	0.72	0.83	0.41	-0.18	-0.12	-0.08	-0.35	0.07	0.03	0.32	-0.31
ERM	5tud-erm		0.16	0.1	0.07	-0.38	0.21	-0.1	-0.2	-0.25	0.07	0.09	0.02	0.07	-0.1	0.07	0.07	0.21	-0.15	0.08	0.06	-0.01	-0.04	-0.01	0.11	-0.04	0.23	0.21	0.07	0.14	0.16	0.16	0.29	0.14	0.23	0.16	0.31	0.35	0.31	0.5	0.52	0.55	0.56	1	0.6	0.48	0.57	0.67	0.42	-0.17	-0.23	-0.05	-0.17	0.06	0.04	0.22	-0.19
7LD	5tvn-7ld		0.28	0.29	0.05	-0.16	0.15	-0.27	0.13	-0.08	0.19	0.16	0.21	0.05	0.02	0.09	0.09	0.11	0.01	-0.08	0.08	0.08	0.07	0.08	0.11	-0.02	0.08	0.14	0.05	0.27	0.41	0.43	0.46	0.35	0.49	0.41	0.28	0.22	0.28	0.61	0.63	0.45	0.44	0.61	1	0.72	0.57	0.67	0.52	-0.06	-0.16	-0.02	-0.3	-0.01	-0.05	0.19	-0.02
H8G	6drx-h8g		0.19	0.22	-0.05	-0.18	0.23	-0.25	-0.07	-0.21	0.12	0.09	0.14	-0.05	-0.1	-0.02	-0.02	0.23	0	-0.16	0.09	0.01	-0.01	0.01	0.15	-0.14	0.15	0.25	-0.05	0.17	0.34	0.36	0.43	0.29	0.46	0.34	0.32	0.35	0.31	0.51	0.54	0.38	0.45	0.46	0.67	1	0.6	0.71	0.46	-0.06	-0.16	0.02	-0.59	0.08	-0.04	0.25	-0.02
H8D	6dry-h8d		0.05	0.1	0.08	-0.32	0.23	-0.23	-0.1	-0.19	0.05	0.09	0.04	0.08	-0.12	0.09	0.09	0.25	-0.13	-0.03	-0.02	-0.07	-0.11	-0.07	0.03	-0.02	0.15	0.25	0.08	0.16	0.16	0.16	0.29	0.12	0.21	0.16	0.24	0.27	0.23	0.42	0.38	0.44	0.5	0.54	0.53	0.6	1	0.78	0.53	-0.17	-0.21	-0.16	-0.14	-0.07	-0.1	0.11	0.02
H8J	6drz-h8j		0.22	0.15	0.12	-0.14	0.18	-0.04	0.2	0	0.16	0.04	0.07	0.12	0.1	0.13	0.13	0.28	0.03	-0.03	0.15	0.06	0.04	0.06	0.08	0.02	0.11	0.16	0.12	0.33	0.16	0.16	0.35	0.14	0.26	0.16	0.33	0.37	0.34	0.46	0.48	0.51	0.58	0.64	0.63	0.71	0.78	1	0.56	-0.12	-0.2	-0.16	-0.28	0.02	-0.02	0.16	-0.14
H8M	6ds0-h8m		0.08	0.1	0.12	0.24	0.15	0.35	0.27	0.25	0.26	0.18	0.11	0.12	0.1	0.13	0.13	0.28	0.18	0.05	0.38	0.17	0.15	0.15	0.1	0.02	0.16	0.12	0.12	0.05	0.16	0.16	0.19	0.14	0.23	0.16	-0.03	-0.01	-0.05	0.29	0.32	0.2	0.25	0.38	0.46	0.43	0.51	0.53	1	-0.19	-0.21	-0.11	-0.25	-0.1	-0.08	0.14	-0.19
AZJ	4ntj-azj	P2Y12	-0.23	-0.13	-0.29	-0.6	-0.22	-0.46	-0.53	-0.46	-0.13	-0.19	-0.08	-0.29	-0.34	-0.27	-0.27	-0.24	-0.31	-0.4	-0.32	-0.21	-0.3	-0.21	-0.26	-0.41	-0.24	-0.2	-0.29	-0.23	-0.02	-0.12	-0.06	-0.02	-0.09	-0.02	-0.06	-0.06	0.01	-0.12	-0.13	-0.15	-0.13	-0.16	-0.07	-0.07	-0.17	-0.12	-0.14	1	0.36	-0.28	-0.43	-0.02	-0.01	-0.07	0.12
6AD	4pxz-6ad		-0.44	-0.23	-0.51	-0.82	-0.25	-0.81	-0.97	-0.77	-0.27	-0.27	-0.16	-0.51	-0.68	-0.51	-0.51	-0.47	-0.5	-0.57	-0.55	-0.37	-0.48	-0.37	-0.39	-0.67	-0.27	-0.3	-0.51	-0.25	-0.2	-0.17	-0.26	-0.02	-0.14	-0.2	-0.07	-0.07	-0.14	-0.24	-0.19	-0.22	-0.2	-0.34	-0.21	-0.25	-0.34	-0.31	-0.33	0.52	1	-0.54	-0.65	-0.21	-0.16	-0.05	-0.21
1Q5	4z9g-1q5	CRF1	-0.16	-0.13	-0.31	-0.42	-0.15	-0.46	-0.23	-0.27	-0.13	-0.19	-0.18	-0.31	-0.4	-0.29	-0.29	-0.15	-0.32	-0.19	-0.24	-0.26	-0.23	-0.26	-0.2	-0.43	-0.08	-0.2	-0.31	-0.22	-0.17	-0.17	-0.21	-0.04	-0.14	-0.17	-0.11	-0.23	-0.15	-0.07	-0.01	-0.01	-0.04	-0.03	-0.01	0.01	-0.12	-0.12	-0.09	-0.21	-0.21	1	-0.68	-0.18	-0.25	-0.2	-0.1
73R	5t1a-73r	CCR2	-0.45	-0.42	-0.42	-0.56	-0.33	-0.62	-0.8	-0.62	-0.43	-0.31	-0.41	-0.42	-0.6	-0.42	-0.42	-0.39	-0.37	-0.41	-0.55	-0.5	-0.51	-0.5	-0.28	-0.45	-0.34	-0.33	-0.42	-0.45	-0.48	-0.48	-0.5	-0.47	-0.49	-0.48	-0.43	-0.43	-0.44	-0.25	-0.26	-0.22	-0.2	-0.14	-0.26	-0.52	-0.12	-0.23	-0.22	-0.36	-0.34	-0.77	1	-0.11	-0.08	-0.37	-0.69
8ES	5unf-8es	AGTR2	-0.16	-0.08	-0.42	-0.5	-0.03	-0.4	-0.73	-0.54	-0.07	-0.1	-0.09	-0.42	-0.5	-0.42	-0.42	-0.1	-0.13	-0.37	-0.15	-0.15	-0.27	-0.15	0.05	-0.57	-0.04	-0.08	-0.42	-0.19	-0.16	-0.16	-0.06	-0.14	-0.1	-0.16	-0.16	-0.14	-0.16	0.02	0	0.02	0.06	0.07	-0.01	0.1	-0.08	0.02	-0.11	-0.02	-0.15	-0.28	-0.16	1	0.8	0.06	-0.29
8EM	5unh-8em		-0.09	-0.03	-0.36	-0.46	-0.01	-0.19	-0.67	-0.33	-0.04	0	0	-0.36	-0.42	-0.38	-0.38	-0.07	-0.1	-0.32	0	-0.13	-0.13	-0.13	0	-0.53	-0.02	-0.05	-0.36	-0.12	-0.23	-0.23	-0.15	-0.2	-0.18	-0.23	-0.3	-0.29	-0.3	-0.1	-0.11	-0.01	0.03	0.05	-0.06	-0.05	-0.13	-0.02	-0.09	-0.01	-0.11	-0.44	-0.13	0.88	1	0.07	-0.38
AQD	5wiv-aqd	DRD4	-0.09	-0.01	-0.36	-0.34	0.1	-0.31	-0.97	-0.33	0.16	0.08	0.11	-0.36	-0.64	-0.35	-0.35	-0.03	-0.13	-0.19	0.06	0.1	-0.07	0.1	0.2	-0.49	0.18	0.14	-0.36	-0.14	0.36	0.28	0.43	0.46	0.37	0.36	0.3	0.34	0.29	0.42	0.44	0.33	0.31	0.31	0.3	0.37	0.17	0.24	0.22	-0.11	-0.05	-0.36	-0.65	0.06	0.02	1	-0.36
RET	6fuf-ret	GNAO	-0.06	0.01	-0.09	-0.44	-0.12	-0.33	-0.27	-0.33	-0.07	-0.07	-0.05	-0.19	-0.22	-0.16	-0.16	-0.21	-0.22	-0.32	-0.23	-0.14	-0.21	-0.14	-0.11	-0.29	-0.15	-0.12	-0.19	-0.06	-0.1	-0.01	-0.01	-0.01	0.04	-0.1	-0.14	-0.14	-0.07	-0.04	-0.06	-0.11	-0.08	-0.06	0.02	0.02	0.01	-0.07	0	0.06	-0.06	-0.07	-0.42	-0.12	-0.15	-0.08	1

Figure S11: Matrix of the normalized scores for the 10 digit profiling scheme using GPCRtm Scoring with a miss and gap penalty of -2. Each element has been coloured following a RdYlGn palette, where higher scores are green in colour, while the lower scores are red. The elements coloured in azure are all values that are >0.5 as these are scores that are of interest.

MOST PROMISING CPG ALIGNMENTS

In this section, we reported the most promising results we achieved across the matrices reported in Fig. S8-S11. To compose Tab. S3, we selected the ligand/GPCR pairs with a CPG score higher than 0.5 and formed by unrelated GPCRs.

A)

TARGET PROTEIN	REFERENCE PROTEIN	SCORE
5tvn-7ld	4iar-erm	0.73
5tvn-7ld	4iaq-2gm	0.72
4iar-erm	5cxv-ohk	0.64
5tvn-7ld	3d4s-tim	0.63
5wiv-aqd	2rh1-cau	0.62
5wiv-aqd	5d6l-cau	0.62
4iar-erm	3d4s-tim	0.62
4ib4-erm	4iaq-2gm	0.62
3uon-qnb	3ny8-jrz	0.61
5dsg-0hk	3ny8-jrz	0.61
5tvn-7ld	2rh1-cau	0.6
5tvn-7ld	5d6l-cau	0.6
6drz-h8j	3uon-qnb	0.6
6drz-h8j	5dsg-ohk	0.6
4iar-erm	2rh1-cau	0.59
4iar-erm	5d6l-cau	0.59
4nc3-erm	5cxv-ohk	0.59
5wiv-aqd	3ny9-jsz	0.58
4iar-erm	3uon-qnb	0.58
4iar-erm	5dsg-ohk	0.58
4nc3-erm	4iaq-2gm	0.58
4iaq-2gm	5cxv-ohk	0.57
4iaq-2gm	3d4s-tim	0.55
4iar-erm	3ny9-jsz	0.55

B)

TARGET PROTEIN	REFERENCE PROTEIN	SCORE
4ib4-erm	4iaq-2gm	0.66
4iar-erm	6drz-h8j	0.63
5tvn-7ld	4iar-erm	0.63
5tvn-7ld	4iaq-2gm	0.61
4iar-erm	3nya-jtz	0.6
4nc3-erm	4iaq-2gm	0.6
3uon-qnb	3ny8-jrz	0.59
4iaq-2gm	6drz-h8j	0.58
4iar-erm	2rh1-cau	0.58
4iar-erm	3d4s-tim	0.58
4iar-erm	5d6l-cau	0.58
4iar-erm	3ny8-jrz	0.57
6drx-h8g	4iar-erm	0.57
5tvn-7ld	3nya-jtz	0.56
4iaq-2gm	3nya-jtz	0.55
4iar-erm	3ny9-jsz	0.55
6drx-h8g	4iaq-2gm	0.55
6drx-h8g	3nya-jtz	0.54
3uon-qnb	3nya-jtz	0.54
4iaq-2gm	2rh1-cau	0.54
4iaq-2gm	3d4s-tim	0.54
4iaq-2gm	5d6l-cau	0.54
3ny9-jsz	5tvn-7ld	0.53
3d4s-tim	5tvn-7ld	0.52

C)

TARGET PROTEIN	REFERENCE PROTEIN	SCORE
5tvn-7ld	4iar-erm	0.75
5tvn-7ld	4iaq-2gm	0.73
5tvn-7ld	3d4s-tim	0.64
4iar-erm	5cxv-0hk	0.64
4ib4-erm	4iaq-2gm	0.64
3uon-qnb	3ny8-jrz	0.62
5dsg-0hk	3ny8-jrz	0.62
4iar-erm	3d4s-tim	0.62
5wiv-aqd	2rh1-cau	0.62
5wiv-aqd	5d6l-cau	0.62
4nc3-erm	5cxv-0hk	0.61
5tvn-7ld	2rh1-cau	0.61
5tvn-7ld	5d6l-cau	0.61
6drz-h8j	3uon-qnb	0.61
6drz-h8j	5dsg-0hk	0.61
4iar-erm	2rh1-cau	0.6
4iar-erm	5d6l-cau	0.6
4nc3-erm	4iaq-2gm	0.6
4iar-erm	3uon-qnb	0.59
4iar-erm	5dsg-0hk	0.59
5wiv-aqd	3ny9-jsz	0.59
4iaq-2gm	5cxv-0hk	0.58
4iar-erm	6drz-h8j	0.57
6drz-h8j	5cxv-0hk	0.57

D)

TARGET PROTEIN	REFERENCE PROTEIN	SCORE
4ib4-erm	4iaq-2gm	0.66
4nc3-erm	4iaq-2gm	0.63
5tvn-7ld	4iar-erm	0.63
5tvn-7ld	4iaq-2gm	0.61
4iaq-2gm	6drz-h8j	0.57
3uon-qnb	3ny8-jrz	0.54
4iar-erm	3nya-jtz	0.54
6drx-h8g	4iar-erm	0.54
4iar-erm	2rh1-cau	0.53
4iar-erm	3d4s-tim	0.53
4iar-erm	5d6l-cau	0.53
4iaq-2gm	6dry-h8d	0.52
4iar-erm	3ny8-jrz	0.51
6drx-h8g	4iaq-2gm	0.51
4iar-erm	3ny9-jsz	0.5
5tud-erm	4iaq-2gm	0.5

Table S3: A comprehensive collection of the results we obtained applying CPG's workflow. The top 25 ligand/GPCR pairs have been gathered for each combination of profiling scheme and matching matrix. A) the top 25 results obtained with the combination 8DP-Blosum62; B) the top 25 results obtained with the combination 10DP-Blosum62; C) the top 25 results obtained with the combination 8DP- GPCRtm; D) the top 25 results obtained with the combination 10DP-GPCRtm.

SUPPLEMENTARY RESULTS TABLES

The following tables refer to the interactions described in the results section of the manuscript. Such tables have been produced in order to display the distances between the ligands and the surrounding residues, both for the original experimental structures and the outcomes of the docking calculations.

	6DRX	
	LISURIDE	ALPRENOLOL
TRP131	3.5 Å	3.9 Å
ASP135	1.7 Å, 2.0 Å	2.1 Å, 2.0 Å
VAL136	3.2 Å	2.6 Å
PHE217	3.2 Å	3.0 Å
PHE340	2.7 Å	2.7 Å
PHE341	3.3 Å	2.9 Å
ASN344	3.0 Å	3.5 Å
VAL366	2.9 Å	2.6 Å

	3NYA	
	ALPRENOLOL	LISURIDE
TRP109	3.0 Å	2.5 Å
ASP113	2.1 Å, 1.8 Å	1.9 Å. 1.6 Å
VAL114	2.8 Å	2.8 Å
TYR199	3.5 Å	3.1 Å
PHE289	3.5 Å	3.2 A
PHE290	2.8 Å	2.7 Å
ASN293	2.5 Å	3.5 Å
TYR308	//	2.2 Å
ASN312	1.8 Å, 2.2 Å	//

Table S4: Schematic depiction of the ligand/GPCR interactions portrayed in the PDB ID: 6DRX and 3NYA (5HT2B and ADRB2, respectively). On the left, a table reporting the binding distances between lisuride and alprenol with 6DRX's binding site, as extrapolated from the crystallographic structure and the docking pose, respectively. On the right, the binding distances between alprenol and lisuride with 3NYA's binding site, as extrapolated from the crystallographic structure and the docking pose, respectively.

	5TVN	
	LSD	TIMOLOL
TRP131	2.6 Å	4.0 Å
ASP135	1.4 Å	2.3 Å, 1.9 Å
VAL136	3.2 Å	2.3 Å
THR140	3.2 Å	2.2 Å
PHE217	3.1 Å	2.3 Å
PHE340	2.6 Å	2.6 Å
PHE341	3.2 Å	2.1 Å
ASN344	3.2 Å	3.0 Å
VAL366	2.8 Å	2.4 Å

	3D4S	
	TIMOLOL	LSD
TRP109	2.9 Å	3.3 Å
ASP113	1.8 Å, 2.2 Å	3.0 Å
VAL114	3.0 Å	2.4 Å
THR118	2.8 Å	2.8 Å
TYR199	2.1 Å	3.5 Å
PHE289	3.5 Å	2.9 Å
PHE290	2.9 Å	2.8 Å
ASN293	2.1 Å	4.2 Å
ASN312	1.9 Å, 1.6 Å	//

Table S5: Tables reporting the ligand/GPCR binding distances present in the PDB ID: 5TVN and 3D4S (5HT2B and ADRB2, respectively). On the left, a collection of the interactions between LSD and timolol with the binding site of 5TVN, as extrapolated from the crystallographic structure and the docking pose, respectively. On the right, timolol binding interactions in the PDB 3D4S compared to the outcome of the docking calculation that LSD underwent inside 3D4S's binding pocket.

	3UON	
	QNB	ICI-118,551
ASP103	2.6 Å	1.7 Å, 2.3 Å
TYR104	2.8 Å	2.1 Å
TRP155	3.1 Å	3.5 Å
PHE195	2.2 Å	2.8 Å
TRP400	3.0 Å	2.2 Å
TYR403	2.7 Å	2.8 Å
ASN404	1.7 Å, 2.3 Å	//
TYR426	3.3 Å	2.7 Å

	3NY8	
	ICI-118,551	QNB
ASP113	1.7 Å	2.0 Å
VAL114	3.5 Å	3.3 Å
TYR199	2.9 Å	3.1 Å
PHE289	3.2 Å	2.7 Å
PHE290	2.8 Å	1.8 A
ASN293	//	3.1 Å
TYR308	2.8 Å	2.2 Å
ASN312	1.8 Å, 2.1 Å	//

Table S6: Tables reporting the distances of the interaction network in the PDB ID: 3UON and 3NY8 (ACM2 and ADRB2, respectively). On the left, the collected data for the crystallographic structure of QNB/3UON and ICI-118,551/3UON's most significant docking output. On the right, the binding distances established by ICI-118,551 and QNB inside 3NY8's orthosteric cavity, extrapolated from the crystal structure and the docking pose, respectively.

CPG USER GUIDE

Installation of GUI

To be able to utilise the CPG tool, most importantly, you require VMD to be installed (please refer to the *readme.txt* file for further details about installation and use). Once VMD is installed and launched you can proceed with the following steps (for clarity please refer to Fig S12 and S13):

1. Click on "Tk Console" from the *Extensions* menu in the VMD main window (Fig S12)
2. Once in the VMD TkConsole window (Fig S13), type `cd CPG_1/`
3. Type `source CPG.tcl`
4. Type `ale_tk`

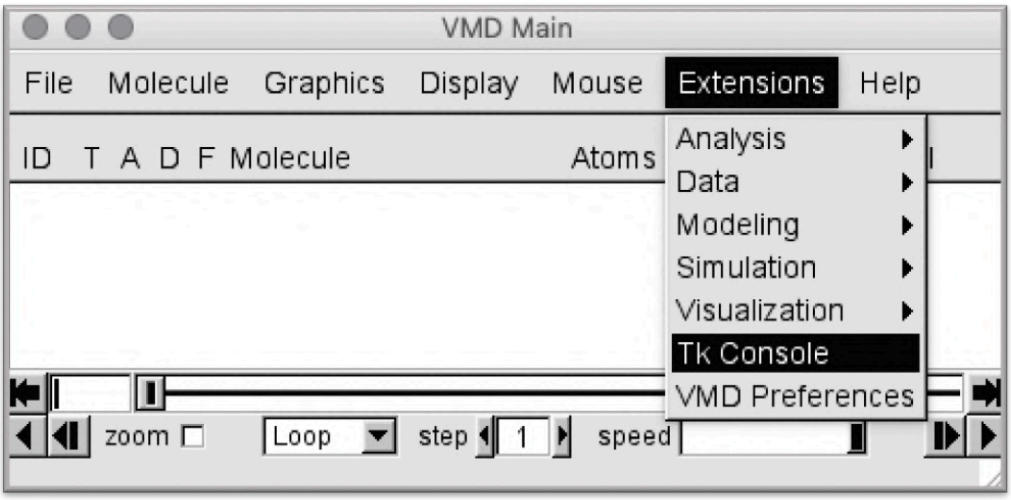

Figure S12: VMD Main window with Tk Console selected from the Extensions menu

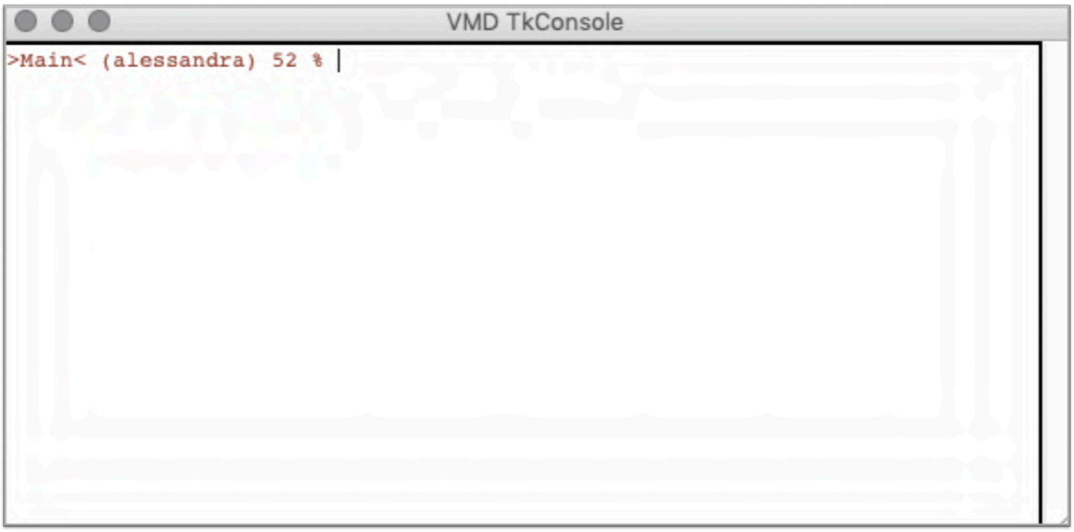

Figure S13: The VMD TkConsole window, needed for typing the installation commands of CPG

CPG tool tutorial

Fig S14 shows the CPG tool in its initial state. Integrated into this tool are a variety of widgets in the form of entries, buttons, and selections to make the alignment process of two proteins user-friendly. What will follow is a short tutorial with explanations of the functions of each of these widgets and the steps that should be executed to visualize the information of one or two GPCRs and how to perform the alignments.

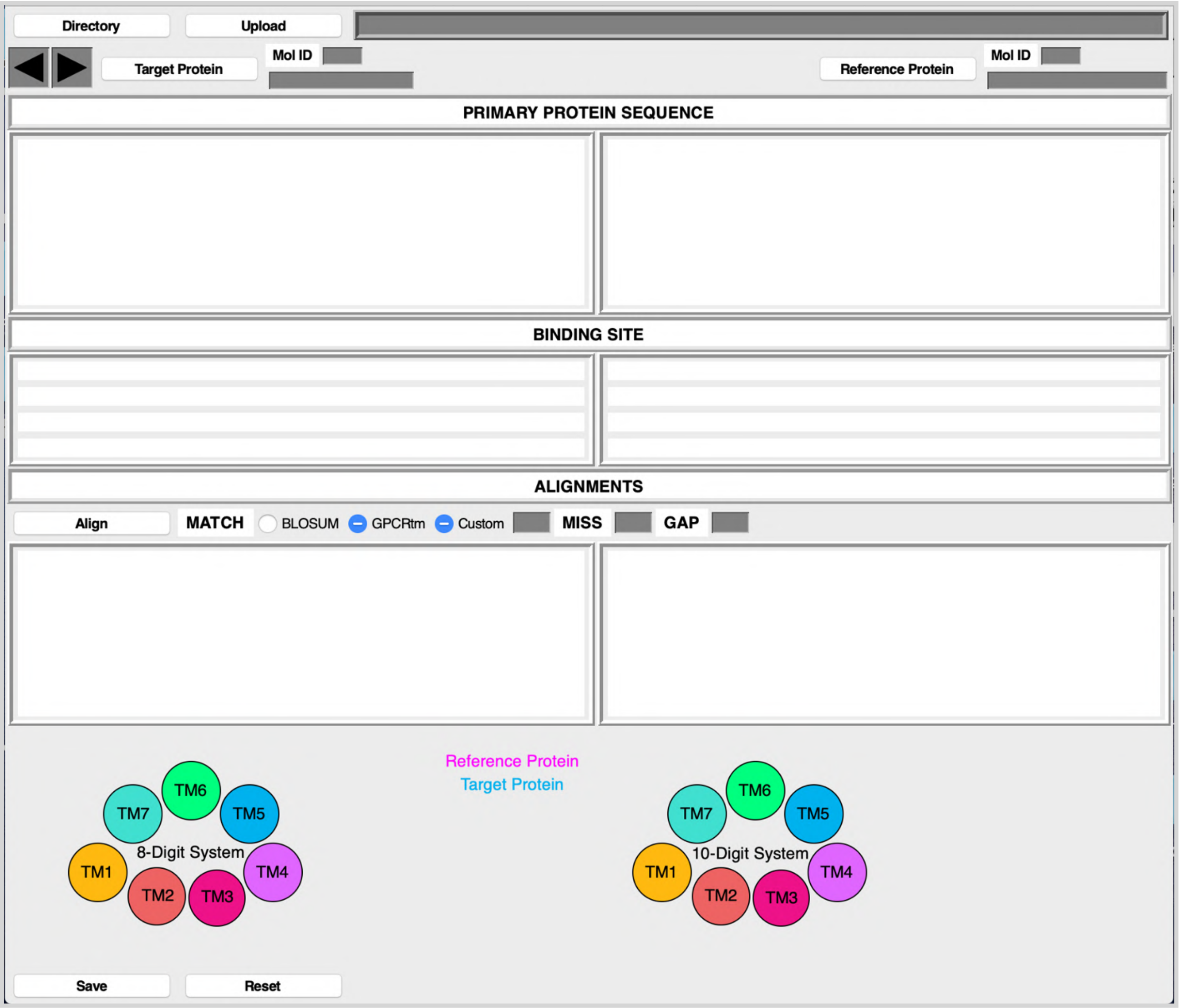

Figure S14: A depiction of the graphical user interface of CPG tool, as displayed in its initial state. The colour scheme of the scaffold may slightly vary between different releases of VMD.

We begin with the header of the CPG tool (Fig S15). This is where the PDB files to be profiled and aligned should be selected and uploaded, and where the PDBs for the "Target" and "Reference" protein molecules can be selected using their VMD PDB "ID" number (Mol ID).

The following steps use the annotations depicted in Fig S15 to describe how to select and upload protein PDB files for profiling, and how to choose target and reference proteins;

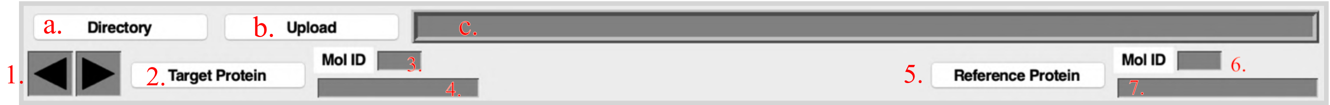

Figure S15: Header of the CPG tool with annotations of the buttons and entry boxes that are required for the uploading and profiling of GPCR PDB files. a) Directory button for selecting directory containing GPCR .pdb files. b) upload button to upload directory to VMD. c) Text box containing directory path. 1) Arrow buttons to select previous and next GPCR. 2) Target protein button to get information about target protein. 3) Text box containing molID number of target protein. 4) Text box containing name of target protein according to file name. 5) Reference protein info button. 6) Entry box for molID number of reference protein. 7) Text box containing name of reference protein according to file name.

1. Click the "Directory" button (a.) to select the directory containing all the PDB files for analysis.
    - The PDB files should be in a single folder to avoid numbering problems later on.
2. Once the directory has been chosen, click the "Upload" button (b.) to upload the files to VMD. All the uploaded pdb files will be reported in VMD Main display (Fig. S16)
    - In the entry box next to the upload button (c.) the directory path will be displayed.

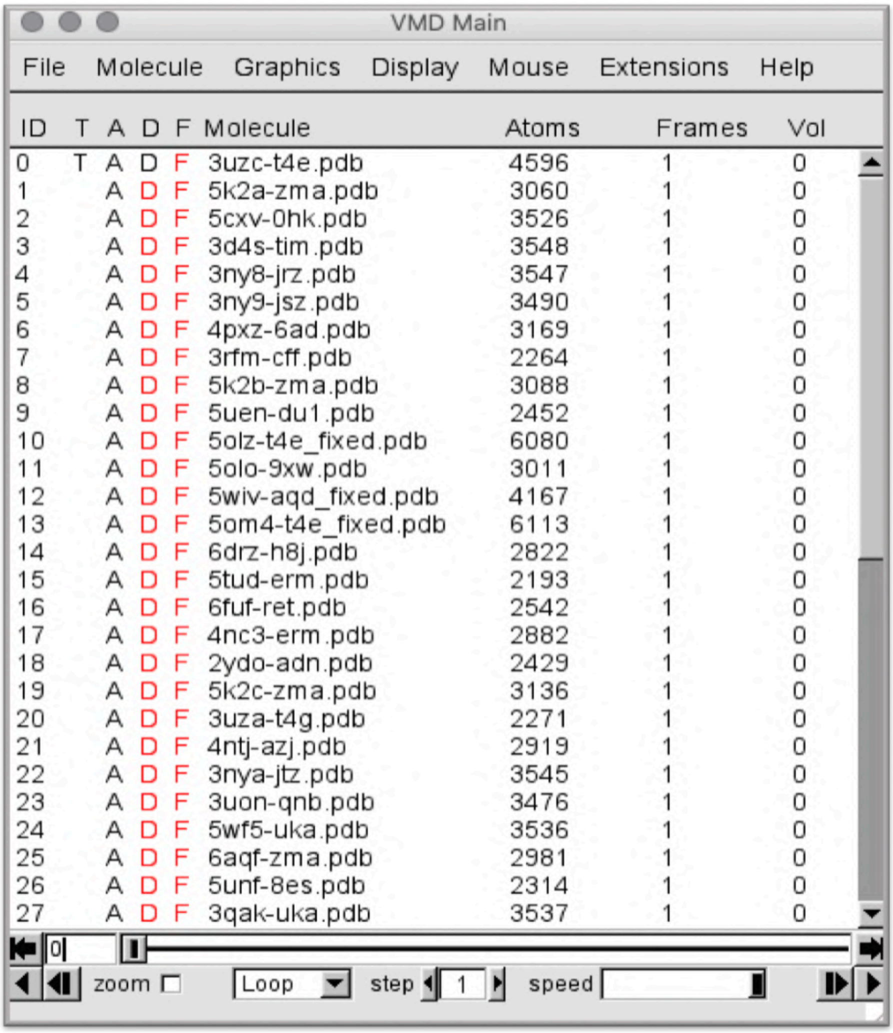

Figure S16: VMD Main display after uploading PDB files using the CPG tool

3. Move with the arrow buttons (1.) to choose the target protein. Then, click on the "Target Protein" button (2.).
    - The Mol ID of the selected protein will be depicted in 3., and its name will be shown in 4.
4. To choose a "Reference" protein, type a "Mol ID" a in the entry box 6. Then, press the “Reference protein” in 5., the name of the "Reference" protein will be shown in 7.

When the "Target Protein" and the “Reference protein” commands have been executed, the "PRIMARY PROTEIN SEQUENCE" frame will display the protein sequences, and the "BINDING SITE" frame will display info about their binding site, i.e. the "Indices" of the residues in the ligand’s surrounding, the "Primary Sequence" of such amino acids, and, lastly, the "8 Digit profile" and "10 Digit profile" of the binding site (see Fig S17).

- Please note that all the information related to the target protein will be depicted on the left, while the data about the reference protein will be shown on the right.

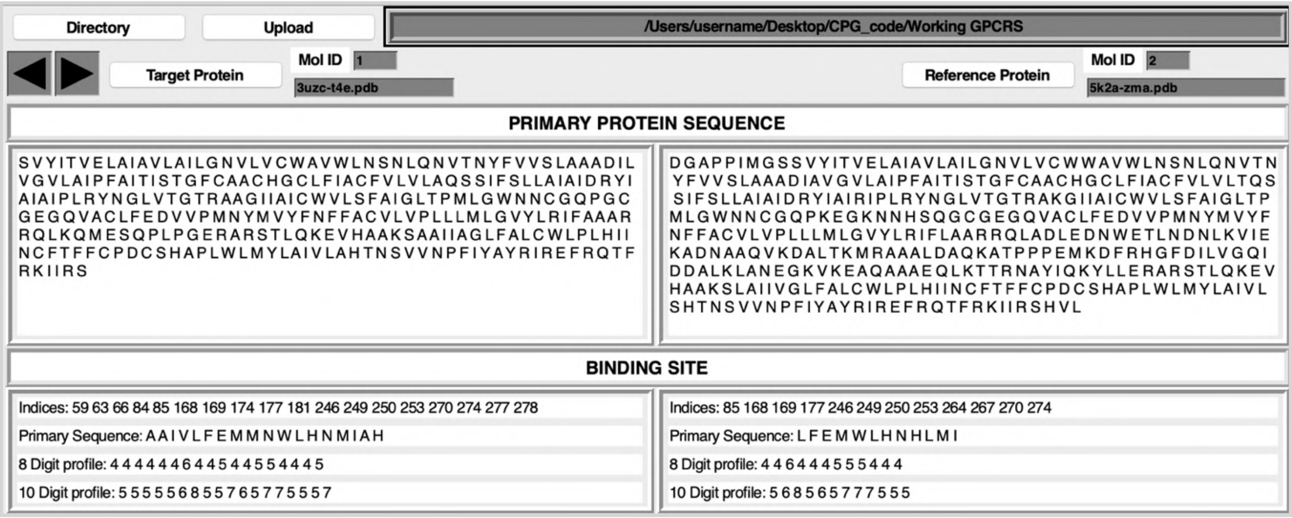

Figure S17: CPG Tool "PRIMARY PROTEIN SEQUENCE" and "BINDING SITE" result after uploading PDB files of GPCRs and selecting the "Target Protein" and "Reference Protein" buttons 2. and 5., respectively.

As well as information depicted in the aforementioned items, the 8DP and 10DP data the binding site are also placed next to their relative helices in a schematic representation of the 7TM helices of the GPCR (refer to Fig S18), where the target protein binding site profile is represented in cyan and the reference protein in magenta.

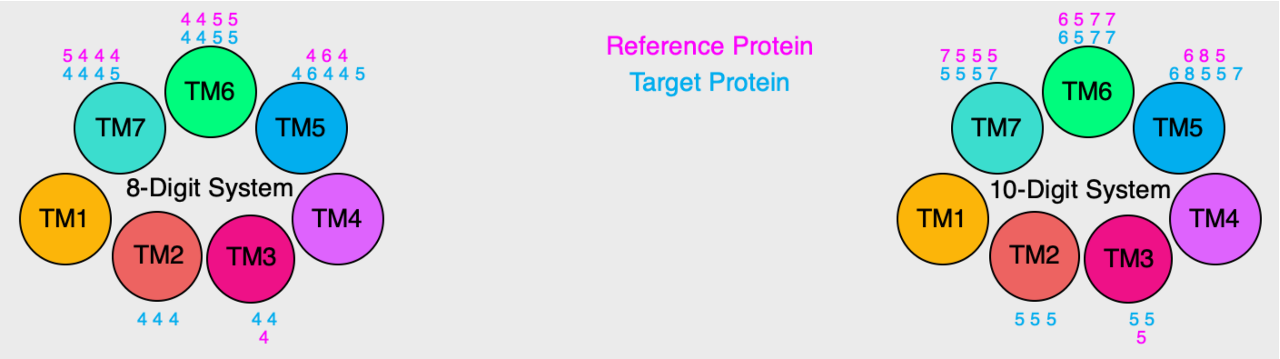

Figure S18: CPG Tool schematic representation of 8DP and 10DP binding site profile results, after selecting the "Target Protein" and "Reference Protein" buttons, 2. and 5. respectively.

Now that the target and reference proteins have been uploaded and their information displayed, it is now possible to perform the binding site profile alignment. For the following steps please refer to the annotated Fig S19.

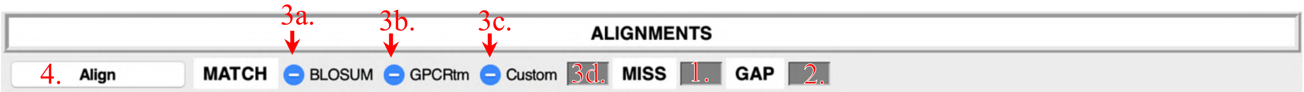

Figure S19: Annotated "ALIGNMENTS" section of the CPG tool depicting the entry boxes, selections, and buttons required to align a target and reference protein once steps 1-5 have been followed. Descriptions for the annotations are as follows; 1) MISS entry box. 2) GAP entry box. 3a) BLOSUM62 Match score selection button. 3b) GPCRtm Match score selection button. 3c) Custom Match score selection button. 3d) Custom Match score entry box. 4) Alignment button.

1. Type non-positive values in the entry boxes for "MISS" (1.) and "GAP" (2.) to select a penalty score when a miss-match or gap creation is required during the alignment.
2. Select a "MATCH" scoring method through one of the three radio buttons.
    A. If "BLOSUM" (3a.) is selected, the scoring method for the matching of aligned profiled residues will be based on BLOSUM62 scoring matrix
    B. If "GPCRtm (3b.) is selected, the scoring method for the matching of aligned profiled residues will be based on the GPCRtm scoring matrix
    C. If "Custom" (3c.) is selected, an integer positive value should be entered in the entry box (3d.).
3. Once all the parameters have been entered, press the "Align" button (4.) to align the 8DP and 10DP profiles (left and right frames, respectively). Refer to Fig S20 to see an example of such outcome.

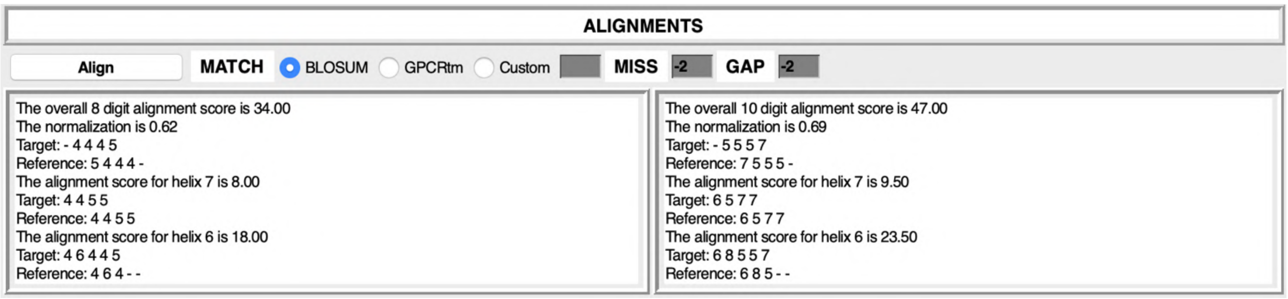

Figure S20: CPG tool result after executing the "Align" button where the left frame indicates the 8 digit profile alignment and the right frame depicts the 10 digit profile alignment. In this example, the BLOSUM 62 scoring has been chosen for the "MATCH" (hit) alignment score while a miss and gap score of -2 has been provided. The alignment results for the 8 and 10 digit alignment scores show the binding profiles for the reference and target.

If the aforementioned steps have been executed correctly, one could expect to generate results such as the example depicted in Fig S21

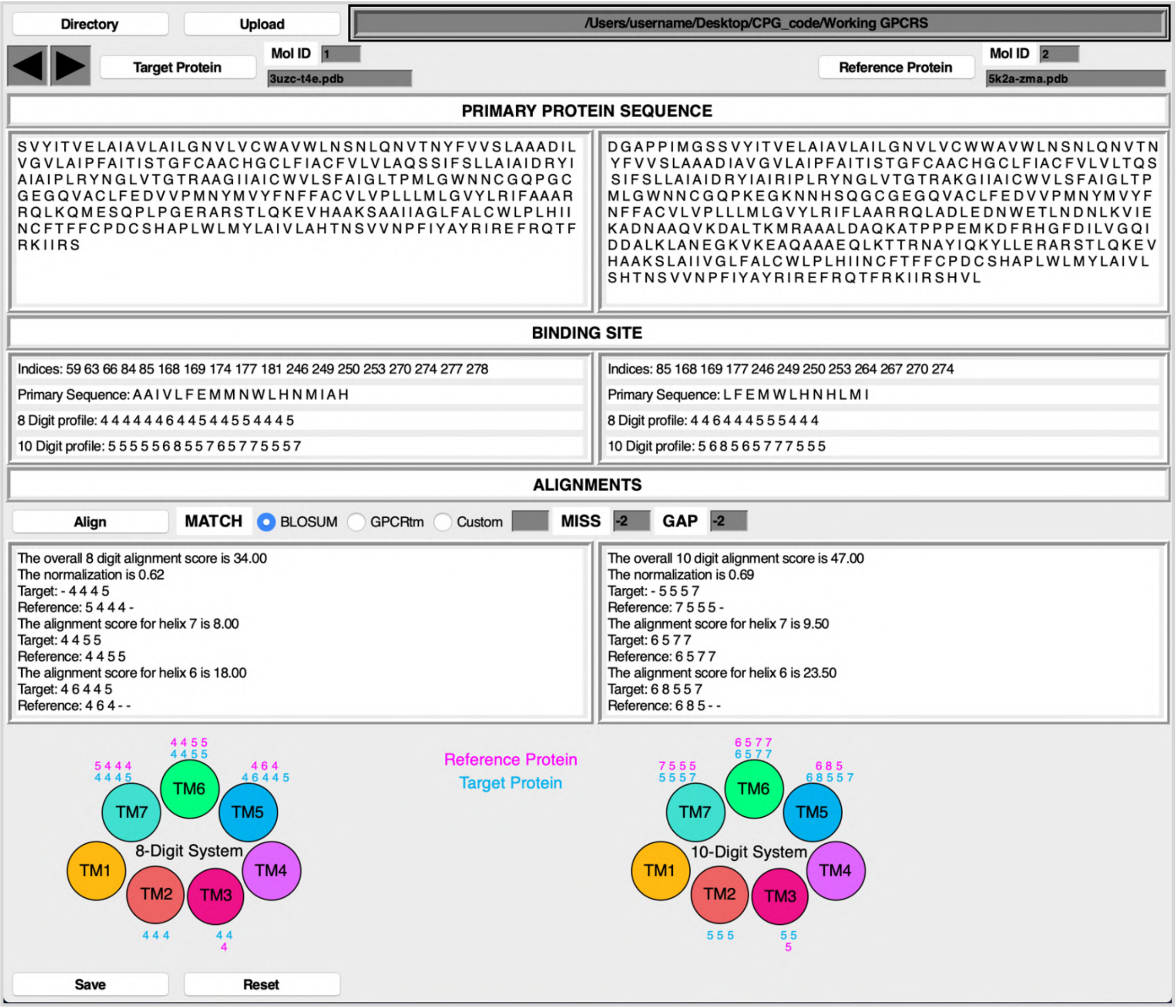

Figure S21: An example of overall results displayed in the CPG's graphical user interface tool after successful execution of all steps.

The alignment results can then be saved by selecting the "Save” button. If one desires to reset the CPG tool and remove all PDB files from VMD, press the “Reset” button (see Fig.S22).

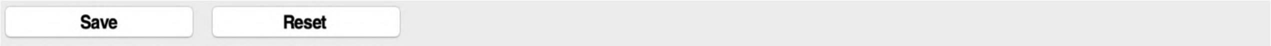

Figure S22: Save and Reset buttons of the CPG Tool

References

1. Choudhuri, S., 2014. Bioinformatiatics for Beginners, Sequence Alignment and Similarity Searching in Genomic Databases: BLAST and FASTA. *Bioinformatics for Beginners: Genes, Genomes, Molecular Evolution, Databases and Analytical Tools. Elsevier Academic Press. 1st edition. p*, pp.133-155.

2. Pietrokovski, S., Henikoff, J.G. and Henikoff, S., 1996. The Blocks database—a system for protein classification. *Nucleic acids research*, *24*(1), pp.197-200.

3. Rios, S., Fernandez, M.F., Caltabiano, G., Campillo, M., Pardo, L. and Gonzalez, A., 2015. GPCRtm: An amino acid substitution matrix for the transmembrane region of class AG Protein-Coupled Receptors. *BMC bioinformatics*, *16*(1), p.206.
